# Size of the Ovulatory Follicle Dictates Spatial Differences in the Oviductal Transcriptome in Cattle

**DOI:** 10.1371/journal.pone.0145321

**Published:** 2015-12-23

**Authors:** Angela María Gonella-Diaza, Sónia Cristina da Silva Andrade, Mariana Sponchiado, Guilherme Pugliesi, Fernando Silveira Mesquita, Veerle Van Hoeck, Ricardo de Francisco Strefezzi, Gustavo R. Gasparin, Luiz L. Coutinho, Mario Binelli

**Affiliations:** 1 Faculdade de Medicina Veterinária e Zootecnia, Universidade de São Paulo, Pirassununga, São Paulo, Brazil; 2 Laboratório de Biotecnologia Animal, Escola Superior de Agricultura Luiz de Queiroz, Universidade de São Paulo, Av Pádua Dias, 11, Piracicaba, SP, Brazil; 3 Faculdade de Medicina Veterinária, Universidade Federal do Pampa, Uruguaiana, Brazil; 4 Faculdade de Zootecnia e Engenharia de Alimentos, Universidade de São Paulo, Pirassununga, São Paulo, Brazil; Friedrich-Loeffler-Institute, GERMANY

## Abstract

In cattle, molecular control of oviduct receptivity to the embryo is poorly understood. Here, we used a bovine model for receptivity based on size of the pre-ovulatory follicle to compare oviductal global and candidate gene transcript abundance on day 4 of the estrous cycle. Growth of the pre-ovulatory follicle (POF) of Nelore (*Bos indicus*) cows was manipulated to produce two groups: large POF large corpus luteum (CL) group (LF-LCL; greater receptivity) and small POF-small CL group (SF-SCL). Oviductal samples were collected four days after GnRH-induced ovulation. Ampulla and isthmus transcriptome was obtained by RNA-seq, regional gene expression was assessed by qPCR, and PGR and ERa protein distribution was evaluated by immunohistochemistry. There was a greater abundance of PGR and ERa in the oviduct of LF-LCL animals thus indicating a greater availability of receptors and possibly sex steroids stimulated signaling in both regions. Transcriptomic profiles indicated a series of genes associated with functional characteristics of the oviduct that are regulated by the periovulatory sex steroid milieu and that potentially affect oviductal receptivity and early embryo development. They include tissue morphology changes (extra cellular matrix remodeling), cellular changes (proliferation), and secretion changes (growth factors, ions and metal transporters), and were enriched for the genes with increased expression in the LF-LCL group. In conclusion, differences in the periovulatory sex steroid milieu lead to different oviductal gene expression profiles that could modify the oviductal environment to affect embryo survival and development.

## Introduction

From the discovery of the oviduct by Gabriele Falloppio in 1561 until the late 1900s, it was believed that the oviduct was simply a conduit for the passage of sperm and oocyte without relevant metabolic or physiological functions [1]. However, it is now well accepted that the oviduct plays a major role in sperm storage and capacitation, fertilization and early embryo development [2–5]. Based on its macro-anatomical characteristics, the oviduct can be divided into three portions: the infundibulum which captures the oocyte after ovulation, the ampulla, which is site of fertilization, and the isthmus, which serves as a sperm reservoir in some species [6] and also transports the embryo to the uterine lumen [7]. Consistent with the functional specialization, cellular and molecular characteristics of the oviduct vary according to region [1, 8]. The oviduct epithelial lumen has two types of cells: ciliated and non-ciliated (secretory) cells [9]. In buffaloes [10] and cattle [8], the relative numbers of secretory cells are significantly greater in the ampulla than in the infundibulum and the isthmus. Additionally, the proportion of secretory cells increases during the follicular phase as compared to the luteal phase of the estrous cycle. Oviductal secretions are comprised of molecules originated from the peripheral circulation as well as molecules synthesized de novo by the luminal epithelial cells [11–13]. Oviductal secretions include nutrients, such as energy substrates, ions and amino acids, and lipids and proteins with structural, catalytic and regulatory functions [14–16].

The function of the female reproductive tract is under constant influence of sex steroidal hormones; they define morphology and physiology of tract components and, ultimately, the ability to support pregnancy. In ruminants, the coordinated and sequential changes of concentrations of ovarian steroids, estradiol (E2) and progesterone (P4) during the estrous cycle regulate oviductal secretory function [17–19]. Indeed, volume of oviductal secretions increases around ovulation [20] and decreases during the luteal phase and pregnancy [21]. More importantly, it has been shown that, in cattle, the proestrus-estrus concentrations of E2 and metaestrus-diestrus concentrations of P4 are positively associated with the probability of pregnancy success [22–24]. There are probably multiple targets of sex-steroid actions that affect fertility, such as the oocyte, oviduct, and endometrium. For example, P4 supplementation during early diestrus regulated the endometrial transcriptome, secretions, and elongation of the conceptus [25–27]. Furthermore, our recent work described a model in which ovulation of larger follicles, and consequent greater proestrus E2 concentrations and early diestrus P4 concentrations, modulated global and specific transcription of endometrial genes in *Bos taurus indicus* cattle [28–30]. However, the modulation of oviductal function by the periovulatory sex steroid milieu remains unstudied in cattle. Here, we hypothesize that distinctly different proestrus-estrus concentrations of E2 and metestrus concentrations of P4 specifically regulate expression of oviductal genes that support pregnancy. To test this hypothesis, we use an in vivo experiment that aimed to 1., produce groups of animals with distinctly different periovulatory endocrine milieus and to discover their effect on: 2., the oviductal regional regulation of gene and protein expression of sex-steroid receptors; 3., the transcriptome of the ampulla and the isthmus and, 4., the regional expression of candidate genes associated with oviductal function in Nelore cows four days after induction of ovulation.

## Materials and Methodology

### Animals

All animal procedures were approved by the Ethics and Animal Handling Committee of the School of Veterinary Medicine and Animal Science of the University of São Paulo. Experiment was carried out at the University of São Paulo, Pirassununga Campus (São Paulo, Brazil). Forty one multiparous and non-lactating Nelore (Bos indicus) cows with no gross reproductive abnormalities by gynecological examination, with a body condition score between 3 and 4 (0, emaciated; 5, obese), were kept in grazing conditions (*Brachiaria brizantha* pastures), supplemented with mineralized salt to fulfill their maintenance requirements, and had free access to fresh water.

### Reproductive management and experimental design

**Fig 1:**
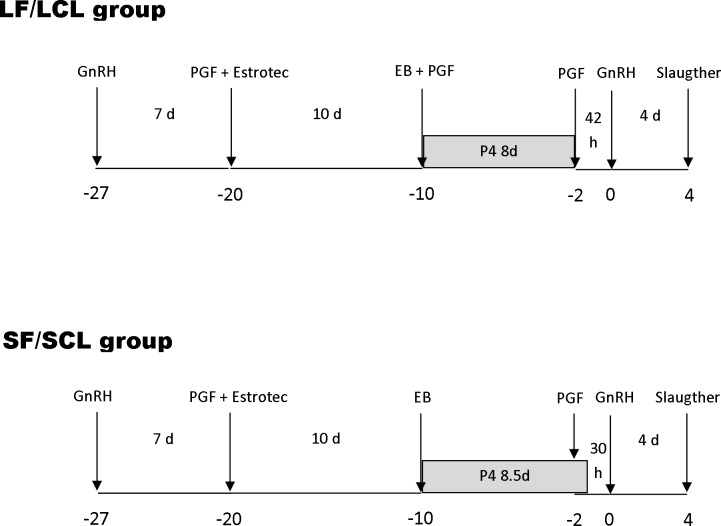
Schematic of the hormonal manipulation protocol used in the present study. Animals (n = 41) were pre-synchronized by intramuscular injection of GnRH agonist and, 7 days later an injection of Prostaglandin F2 alpha (PGF) analog. At this day [day -20 (D-20)] animals received an ESTROTECT Heat detector device and estrus detection was performed twice daily from D-20 to D-10. All animals received a new intravaginal P4-releasing device on D-10 along with an intramuscular injection of 2 mg estradiol benzoate. Simultaneously, cows in the LF-LCL received an intramuscular injection of PGF. The P4 devices were removed on day -2, 42 h and 30 h before the GnRH injection in the LF-LCL and the SF-SCL groups, respectively. All animals received a PGF injection at P4 device removal and a second PGF injection 6 h later. Ovulation was induced by an injection of GnRH agonist on D0.

Animal and reproductive management was performed as described previously [28, 29] to generate animals ovulating a larger follicle and presenting a subsequent larger CL (LF-LCL group, associated with greater receptivity and fertility) or smaller follicles (SF-SCL group; Fig 1). Briefly, animals were pre-synchronized by intramuscular injection of GnRH agonist (1 μg of buserelin acetate; Sincroforte, Ouro Fino, Cravinhos, Brazil) and, 7 days later an injection of Prostaglandin F2 alpha analog (PGF; 0.5 mg of sodium cloprostenol; Sincrocio, Ouro Fino, Cravinhos, Brazil). At this day [day -20 (D-20)] animals received an ESTROTECT Heat detector patch (Rockway, Inc. Spring Valley, WI, USA) and estrus detection was performed twice daily from D-20 to D-10. All animals received an intravaginal P4-releasing device (1g; Sincrogest) on D-10 along with an intramuscular injection of 2 mg estradiol benzoate (Sincrodiol, Ouro Fino). Simultaneously, cows in the LF-LCL received an intramuscular injection of PGF. The P4 devices were removed on day -2, 42 h and 30 h before the GnRH injection in the LF-LCL and the SF-SCL groups, respectively. All animals received a PGF injection at P4 device removal and a second PGF injection 6 h later. Ovulation was induced by an injection of GnRH agonist on D0. In order to assess growth and ovulation of the pre-ovulatory follicle (POF) and CL area, transrectal ultrasound examinations were carried out on D-10 and daily from D-2 to D4, and color Doppler mode was used to detect signals of CL blood flow. Ultrasonography was performed with the aid of a duplex B-mode (gray-scale) and pulsed-wave color Doppler ultrasound instrument (MyLab30 Vet Gold; Esaote Healthcare, São Paulo, São Paulo, Brazil) equipped with a multifrequency linear transducer. All Doppler scans were performed at a constant gain setting for color. Retrospective interpretation of changes in follicular diameters over time allowed the identification of the dominant follicle of the estradiol benzoate-induced wave, the determination of the POF diameter, and the day of ovulation. Ovulation was defined as the disappearance of the previously identified POF followed by the observation of a CL on the same approximate topographical location on the ovary. The diameter of follicles was calculated as the average between measurements of two perpendicular axes of each structure. The maximum CL area was determined using a B-mode still image and the tracing function [31]. For CL with an anechoic fluid-filled cavity, the area of the cavity was subtracted from the total area [32].

### Blood sampling and hormone measurements

Blood sampling for determination of P4 and E2 concentrations was performed once daily from D-2 to D4. Blood samples were collected by jugular venipuncture in tubes containing Heparin (BD, São Paulo, São Paulo, Brazil). Plasma was separated using centrifugation at 4°C, 1500x g for 30 minutes (min), and stored at −20°C. Plasma P4 concentrations were measured in all samples using a solid-phase radioimmunoassay (Coat-a-Count; Siemens, Los Angeles, CA, USA), as validated previously [33]. Plasma E2 concentrations were determined using a commercial RIA kit (Double Antibody Estradiol; Siemens) as reported previously [34]. The intra-assay CV and sensitivity for P4 and E2, respectively, were 0.8% and 0.05 ng/mL, and 1.7% and 0.13 pg/mL.

### Tissue collection and processing

On D4, cows were stunned using a captive bolt and euthanized by jugular exsanguination at the University slaughterhouse. Reproductive tracts were transported on ice and dissected within 15 min of slaughter. The oviduct ipsilateral to the ovary containing the CL was dissected and the ampulla and isthmus regions separated. The ipsilateral side was chosen because it is the side in which oocyte, sperm, zygote, and early embryos will transit during the initial days after estrus. Furthermore, there is evidence suggesting that the ipsilateral and the contralateral sides are controlled by different transcription mechanisms [12, 35, 36].

Tissue from ampulla and isthmus regions, covering all the oviductal thickness, were either frozen in liquid nitrogen or fixed in buffered formalin prior to paraffin embedding. Transcriptome of ampulla and isthmus were evaluated because previous evidence suggested that they were morphologically [8], physiologically [2], and molecularly [37, 38] different. Furthermore regions play distinct roles in the reproductive process.

The D4 was selected because at this point, the oocyte transport, sperm transport, and fertilization have already occurred, and, if successful, an early embryo is expected to be in transit between the ipsilateral ampulla and isthmus. Our interest was to gain understanding of how the differences in the E2 and P4 concentrations during the periovulatory period affect the ampulla and isthmus transcriptome.

### Immunohistochemistry

Paraffin-embedded ampulla and isthmus samples were sectioned into 4 μm sections and mounted onto an adhesive slide (StarFrost, Knittel Glass, Braunschweig, Germany). Sections were deparaffinized in xylene, and rehydrated in a series of increasing dilutions of ethanol. Sections were incubated for 5 min in 10nM citrate buffer (pH 6) at room temperature, and then processed for 4 cycles of 5 min at 750 watts in a microwave for antigen retrieval. Slides were cooled at room temperature for 20 min and washed three times for 5 min in PBS containing 0.3% Triton (PBS/Triton; pH 7.4). Endogenous peroxidase activity was blocked by incubation in 1.5% hydrogen peroxide in methanol for 30 min at room temperature. Sections were washed in PBS/Triton (3 x 5 min). To block nonspecific binding, protein block was carried out using 2% non-fat dry milk for 15 min at room temperature. Tissue sections were incubated in a humid chamber with a mouse monoclonal anti-Progesterone Receptor (PGR) primary antibody (Clone PR10A9; 1:75 dilution; Beckman Coulter PN IM1546; France) overnight at 4°C or anti-Estrogen Receptor alpha mouse monoclonal (ER alpha; clone 1D5, 1:50 dilution; Dako; Denmark) 60 min at room temperature. Technical negative control reactions contained normal mouse IgG instead of primary antibody. Tissue sections were then washed (3x5 min) in PBS/Triton. The Biotinylated Link Universal Solution (DK0690, Dako) was used as secondary antibody, and the incubation was conducted for 15 min at room temperature in a humid chamber. Next, slides were washed in PBS/Triton buffer (3x5 min), incubated with a streptavidin- peroxidase complex (Streptavidin-HRP, K0690, DAKO) for 15 min in a humid chamber, and washed again in PBS/Triton. Sections were then incubated in diaminobenzidine (DAB, K3468, DAKO) solution for 5 min and washed in distilled water (3x5 min). Finally, sections were counterstained with hematoxylin, washed in running water for 10 min, dehydrated in a series of increasing concentrations of ethanol, cleared in xylene, and mounted on coverslips. The immunostaining procedure for each primary antibody was performed in a single run. The slides were photographed using light microscopy and immunostaining was subjectively analyzed (strong, mild, weak or no staining) in the luminal epithelium (LE), stromal cells (SC), muscular layer (ML) and serous membrane (SM).

### RNA and protein isolation

Frozen ampulla and isthmus samples were ground in liquid nitrogen using a mortar and pestle and immediately mixed with buffer RLT from AllPrep® DNA/RNA/Protein Mini kit (No. 80004, Qiagen, São Paulo, São Paulo, Brazil), as recommended by manufacturer’s instructions. Tissue suspension was passed at least 5 times through a 21-ga needle, and centrifuged at 13,000 x g for 3 min for removal of debris, before supernatant loading and processing in silica columns. RNeasy columns were eluted with 30 μL of RNase-free water and both, RNA and protein, were kept at −80°C. Concentration of total RNA extracts was measured using the NanoVue spectrophotometer (GE Healthcare).

### RNA Sequencing (RNA-Seq): procedures and data analysis

Ampulla and isthmus samples of the three top-ranked animals in the LF-LCL group and the three bottom ranked animals in the SF-SCL group were selected for individual RNA-Seq (please see details in the Statistical Analyses section). RNA quality was assessed with the NanoVue spectrophotometer (GE Healthcare Europe, Munich, Germany; 260/280 and 260/230 nm ratios) and with the Agilent Bioanalyzer (Agilent Technologies, Palo Alto, USA; 28S/18S ratio and RNA integrity number (RIN) data). All samples had a RIN ≥8 and were considered suitable for RNAseq analysis. For the analysis of expression profiles in LF-LCL and SF-SCL animals, libraries were generated using a routine RNA library preparation TruSeq protocol developed by Illumina Technologies (San Diego, CA) using 1 μg of total RNA as input. Briefly, polyA selected RNA was cleaved as per Illumina protocol and the cleaved fragments were used to generate first strand cDNA using SuperScript II reverse transcriptase and random hexamers. Subsequently, second strand cDNA was synthesized with RNaseH and DNA polymerase enzyme. Adapter ligation and end repair steps followed second strand synthesis. Resulting products were amplified via PCR and cDNA libraries were then purified and validated using the Bioanalyzer 2100 (Agilent Technologies). Paired-end sequencing of 101bp reads was performed using the Illumina HiSeq 2000 (ampulla samples) and Illumina HiSeq 2500 (isthmus samples) platforms. The quality filtering was performed by seqyClean v1.3.12. (https://bitbucket.org/izhbannikov/seqyclean/get/stable.zip) using a minimum of 26 Phred quality vector, and adaptor sequences from the Univec database (https://www.ncbi.nlm.nih.gov/tools/vecscreen/univec/) were used as guide to remove possible contaminants and minimum read length of 65 bases. Only high quality paired-end sequences were kept for further analyses. The reads were mapped with Tophat v.2.0.8 [39] and Bowtie2 v2.1.0 [40] on the masked bovine genome assembly (Bos taurus UMD 3.1, NCBI). The isoforms were obtained with the package Cufflinks v.2.1.1 [41], with the annotation file (.gtf) as guide through the option -G (the RABT assembly) and specifically the PGR gene isoforms were searched.

The mapping file was sorted using SAMTools v 0.1.18 [42] and read counts were obtained using the script from HTSeq-count v0.5.4p2 (http://www-huber.embl.de/users/anders/HTSeq/doc/count.html). The differential expression analysis was performed with package DESEq v1.12.1 [43], from R/Bioconductor [44]. Using the function “estimate Size Factors”, the normalized counts were obtained (baseMean values, which are the number of reads divided by the size factor or normalization constant). The standard deviation along the baseMean values was also calculated for each transcript. In order to avoid artifacts caused by low expression profiles and high expression variance, only transcripts that had an average of baseMean > 5 and the mean greater than the standard variation were analyzed. The method to test for differential expression was the negative binomial distribution, through the nbinom Test function on DESeq. The threshold for evaluating significance was obtained by applying a p-value of 0.05 FDR- Benjamini-Hochberg [45]. The gene enrichment analysis was performed separately for each region, using the functional annotation tool of the Database for Annotation, Visualization, and Integrated Discovery (DAVID; [46] using as background the set of genes that passed through the differential expression analysis filter. In order to avoid overrepresentation of some GO similar categories, GO terms were grouped when having more than 3 common genes.

### Quantitative PCR

RNA quality and quantity was measured with the NanoVue spectrophotometer and 1 μg was reverse transcribed (High Capacity cDNA Reverse Transcription Kit, Life Technologies) according to manufacturer’s instructions; samples were incubated at 25°C for 10 min, followed by incubation at 37°C for 2 h and reverse transcriptase inactivation at 85°C for 5 min and storage at −20°C. The cDNA obtained was used for gene expression assays by qPCR.

Step-One Plus (Life Technologies, Carlsbad, CA) with SYBR Green Chemistry was used for the amplification analysis. Primers were designed based on GenBank Ref-Seq mRNA sequences of target genes. Sequences were masked to remove repetitive sequences with RepeatMasker (http://www.repeatmasker.org/) [47] and, then, the masked sequences were used to primer design using the PrimerQuest software (IDT1, http://www.idtdna.com/primerquest/Home/Index). The characteristics of the primers were checked in Oligo Analyzer 3.1 software (IDT1, http://www.idtdna.com/analyzer/Applications/OligoAnalyzer/), while the specificity was compared by BLAST1 (NCBI, http://blast.ncbi.nlm.nih.gov). qPCR products from reactions containing designed primers were submitted to agarose gel electrophoresis and sequencing and identities were confirmed. Details of primers are provided on Table 1.

**Table 1:** Primer sequences of target and reference genes analyzed using qPCR.

Target gene	Gene Bank Number	Forward primer sequence (5′–3′)	Reverse sequence (5′–3′)	Primer efficiency (%)	Amplicon length (bp)
*ACTB*	NM_173979.3	GGATGAGGCTCAGAGCAAGAGA	TCGTCCCAGTTGGTGACGAT	2.03	77
*ANGPT2*	NM_001098855.1	ACCCTTCAGGTGAACACTGG	CGTGAGGCCTTTAAGGTGAA	2.03	178
*ANGPT4*	NM_001076483.2	ACCCTCATTCAGCGCCGTGA	GCTGGGTTGCCAAAGCCCTGTT	2.06	83
*CADM3*	NM_001075946.1	AACCTCTCCCAGGACGACAGT	TCTGCTGGGCAGGGTTAGAC	1.93	133
*C-MET*	NM_001012999.2	AGGTCGATTCATGCAGGTTGT	TTTAGCGGGTGCTCCACAAT	1.98	114
*CTGF*	NM_174030.2	CGTGTGCACCGCTAAAGATG	TCCGCTCTGGTACACAGTTCCT	2.06	61
*CTSS*	NM_001033615.2	AGAAGCCGTGGCCAATAAA	CTTCCCGTCAAGGTTACCATAG	2.10	157
*CXCR4*	NM_174301.3	AAAGTGACCCTGAGGACTTGAGTAG	CCGGAAGCAGGGTTCCTT	2.03	153
*EDN1*	NM_181010.2	GAGTGTGTCTACTTCTGCCATC	CTAGCACACTGGCATCTCTTC	2.10	158
*ESR1*	XM_002690343.1	CAGGCACATGAGCAACAAAG	TCCAGCAGCAGGTCGTAGAG	2.05	82
*ESR2*	NM_174051.3	GTAGAGAGCCGCCATGAATAC	CAATGGATGGCTAAAGGAGAGA	1.96	161
*GAPDH*	NM_001034034.2	GCCATCAATGACCCCTTCAT	TGCCGTGGGTGGAATCA	1.93	69
*HISTONA 2*	AY835842.1	GAGGAGCTGAACAAGCTGTTG	TTGTGGTGGCTCTCAGTCTTC	1.93	103
*HPSE*	NM_174082.2	CGGATTGTTGAGAAGATCAGA	AAGGTGTTGGACAGGAAGGG	1.92	94
*HSPA1A*	NM_203322.2	CACCATCACCAACGACAA	CTTGTCCAGCACCTTCTTC	2.08	181
*OVGP1*	NM_001080216.1	CCGCTGGACCTTTGTCTTCT	GAAATCCAGGAGTCTGCCCA	1.93	166
*PCNA*	NM_001034494.1	TTGGCTCCCAAGATCGAGGATGAA	TGTGCTGGCATCTCAGAAGCAGTT	1.95	98
*PDGF*	NM_001083706.1	TCTCTGATCCCAATGCACCG	TCGGTACAAGTCATCTCGCC	1.99	147
*PGR*	NM_001205356.1	GCCGCAGGTCTACCAGCCCTA	GTTATGCTGTCCTTCCATTGCCCTT	1.97	199
*PGR1*	NM_001202474.3	ACTACCTGAGGCCGGATT	CCCTTCCATTGCCCTCTTAAA	2.03	163
*PGRMC2*	NM_001099060.1	CAGGGGAAGAACCGTCAGAA	ATGAAGCCCCACCAGACATT	1.98	168
*PPIA*	NM_178320.2	GCCATGGAGCGCTTTGG	CCACAGTCAGCAATGGTGATCT	2.02	64
*RGS20*	NM_001076327.1	CGTCTAGGACAGGGACTTTAGA	GAACACACTACTGCCACCATAA	2.09	198
*RPL15*	NM_001077866.1	TGGAGAGTATTGCGCCTTCTC	CACAAGTTCCACCACACTATTGG	1.99	64
*TGFB2*	NM_001113252.1	AATTTGGTGAAGGCCGAGTTC	GGTTTTCACGACTTTGCTCCA	1.95	149
*TGFB3*	NM_001101183.1	TTACTGCTTCCGCAATTTCA	TCTGAGCTGCGGAGGTATG	1.94	149
*TGFBR1*	NM_174621.2	GATTCGGCCACGGATACAA	GTCGAGCTACTTCCCAGAATAC	1.94	165
*TGFBR2*	NM_001159566.1	AAGTCGGTTAACAGCGATATGATG	TCCGGCTTCTCGCAGATG	2.04	154
*VCL*	NM_001191370.1	GCTGTTCAAGGGAGTAATAGG	TTCTGGCTTTGGGAAGAAATA	2.07	153

In order to select reference genes, the GeNorm Microsoft Excel applet was used. This applet provides a measure of gene expression stability (M) [48]. Histone 2, Glyceraldehyde-3-Phosphate Dehydrogenase (GAPDH), Actin Beta (ACTB), Cyclophilin A (PPIA), Ribosomal Protein L15 (RPL15) Ct values were converted to scale expression quantities using the delta-Ct method and entered into geNorm. Genes were ranked based on M values, where the genes with the most stable expression had the lowest values. Data was analyzed in geNorm initially using all five genes and then the two most stable genes (PPIA, GAPDH) were selected. Determination of qPCR efficiency and Cq (quantification cycle) values per sample were performed with LinRegPCR software (V2014.2; http://linregpcr.nl/). Quantification was obtained after normalization of the target genes expression values (Cq values) by the geometric mean of the endogenous control expression values PPIA and GAPDH. In order to validate RNAseq analysis results, qPCR was conducted on a subset of 23 genes. We selected genes from the following functional categories, relevant to oviductal biology: sex-steroid receptors [Estrogen Receptor 1 (ESR1) and 2 (ESR2), progesterone receptor (PGR), progesterone receptor membrane component 2 (PGRMC2), and Regulator Of G-Protein Signaling 20 (RGS20)], angiogenesis [angiopoietin 2 (ANGTP2) and 4 (ANGTP4), and endothelin 1 (EDN1)], cellular proliferation [connective tissue growth factor (CTGF), MET proto-oncogene receptor tyrosine kinase (C-MET), proliferating cell nuclear antigen (PCNA), platelet derived growth factor D (PDGFD), transforming growth factor beta 2 (TGFB2) and 3 (TGFB3), transforming growth factor beta receptor 1 (TGFBR1) and 2 (TGFBR1),], extracellular matrix [cell adhesion molecule 3 (CADM3), chemokine (C-X-C motif) receptor 4 (CXCR4), heparanase (HSPE), and vinculin (VCL)], synthesis of glycoproteins [cathepsin S (CTSS) and oviductal glycoprotein 1 (OVGP1)], and cellular chaperone [Heat Shock 70kDa Protein 1A (HSPA1A)].

### Statistical analyses

Prior to the statistical analyses, animals were evaluated according to the criteria we established in order to determine whether minimal premises were matched. Animals were removed from the experiment if: POF diameter on D0 was smaller than 8 mm, ovulation was detected at the D0 ultrasound examination or before (i.e. early ovulation), ovulation was detected at the D3 ultrasound examination (i.e., late ovulation), ovulation was not detected, and follicular or luteal cysts were detected at any moment during the experiment. According to these criteria, 13 animals of LF-LCL and 8 animals of SF-SCL that ovulated within 24–36 h of GnRH injection were used. In order to select the samples for the different laboratory analyses, animals in each group (LF-LCL and SF-SCL) were ranked according to the following ovarian and endocrine variables: maximum diameter of the POF and E2 concentration at D -1, CL area and P4 concentrations at D4. Then, ampulla and isthmus samples of the top-ranked animals in the LF-LCL group and the bottom ranked animals in the SF-SCL group were selected (RNAseq analyses: 3 animals per group; IHQ analyses: 5 animals per group; qPCR: 7 animals per group).

Data was tested for normality of residuals and homogeneity of variances. Ovarian and endocrine variables were analyzed for the main effect of group (LF-LCL vs. SF-SCL) by one-way ANOVA using the GLM procedure of SAS. The E2 and P4 concentration were analyzed for the main effect of group and time by repeated split-plot ANOVA using SAS PROC GLM. Transcript abundance was analyzed by two-way ANOVA, considering the main effects of group and region (isthmus and ampulla) and the interaction of group by region. All data will be shown as mean ± standard error of mean.

## Results

### Animal model

**Fig 2:**
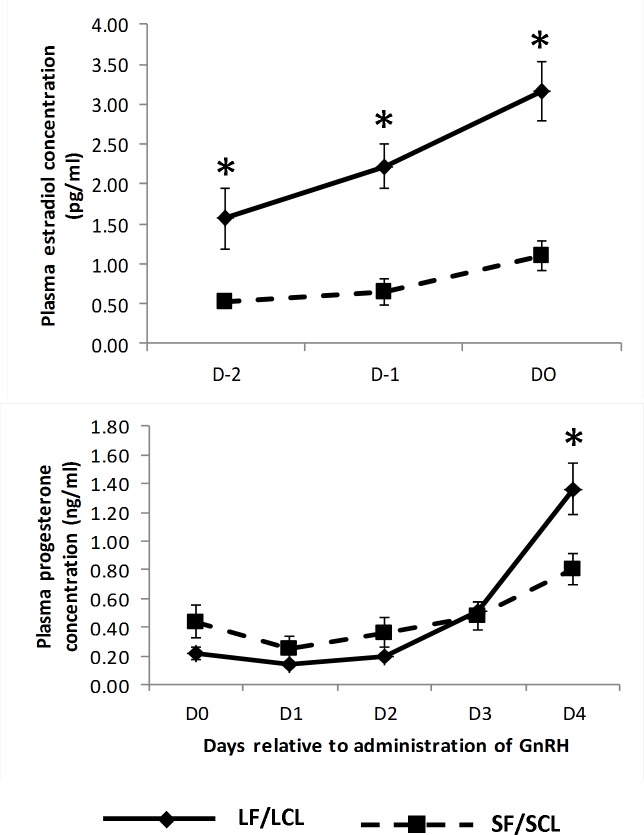
Plasma concentrations (mean ± SEM) of estradiol (top panel) and progesterone (bottom panel) of cows in the LF/LCL group (n = 13) and in the SF/SCL group (n = 8). Within a given Day, significantly different means (P < 0.05) were indicated by an asterisk (*). LF/LCL, large follicle-large CL; SF/SCL, small follicle-small CL.

Forty one animals were synchronized in order to obtain the two experimental groups. Only 21 remain in the experiment (13 animals of LF-LCL and 8 animals of SF-SCL) after fulfilling all the premises required. As planned, hormonal manipulations successfully generated distinctly different groups of animals regarding both ovarian and endocrine variables. Specifically, on D-1, diameter of the POF in LF-LCL (15.70 ± 0.43 mm) was greater than in the SF-SCL group (11.31 ± 0.23 mm; P < 0.01). In agreement with these findings, E2 plasma concentrations were affected by time (P < 0.01) and group (P = 0.02), and were greater in the LF-LCL than in the SF-SCL cows in D-2, D-1 and D0 (Fig 2). Regarding the CL values at D4, a group effect was found in CL area but not in CL blood flow. At D4, CL of LF-LCL animals had an area of 1.39 ± 0.08 mm^2^ and 43.75± 3.83% of blood flow. In comparison, SF-SCL animals achieved 1.02 ± 0.09 mm^2^ of area (P < 0.01) and 36.25± 4.12% blood flow (P >0.1). Plasma P4 concentration was also affected by time (P < 0.01) and group (P = 0.03) effects being greater for LF-LCL than SF-SCL group on day 4 (Fig 2).

Tissue responses to the endocrine changes elicited by the animal model are modulated by specific receptors. Thus, using 7 animals per group, abundance of transcripts of nuclear *ESR1*, *ESR2 and PGR* and membrane *PGRMC-2* were quantified in both oviduct regions.

**Fig 3:**
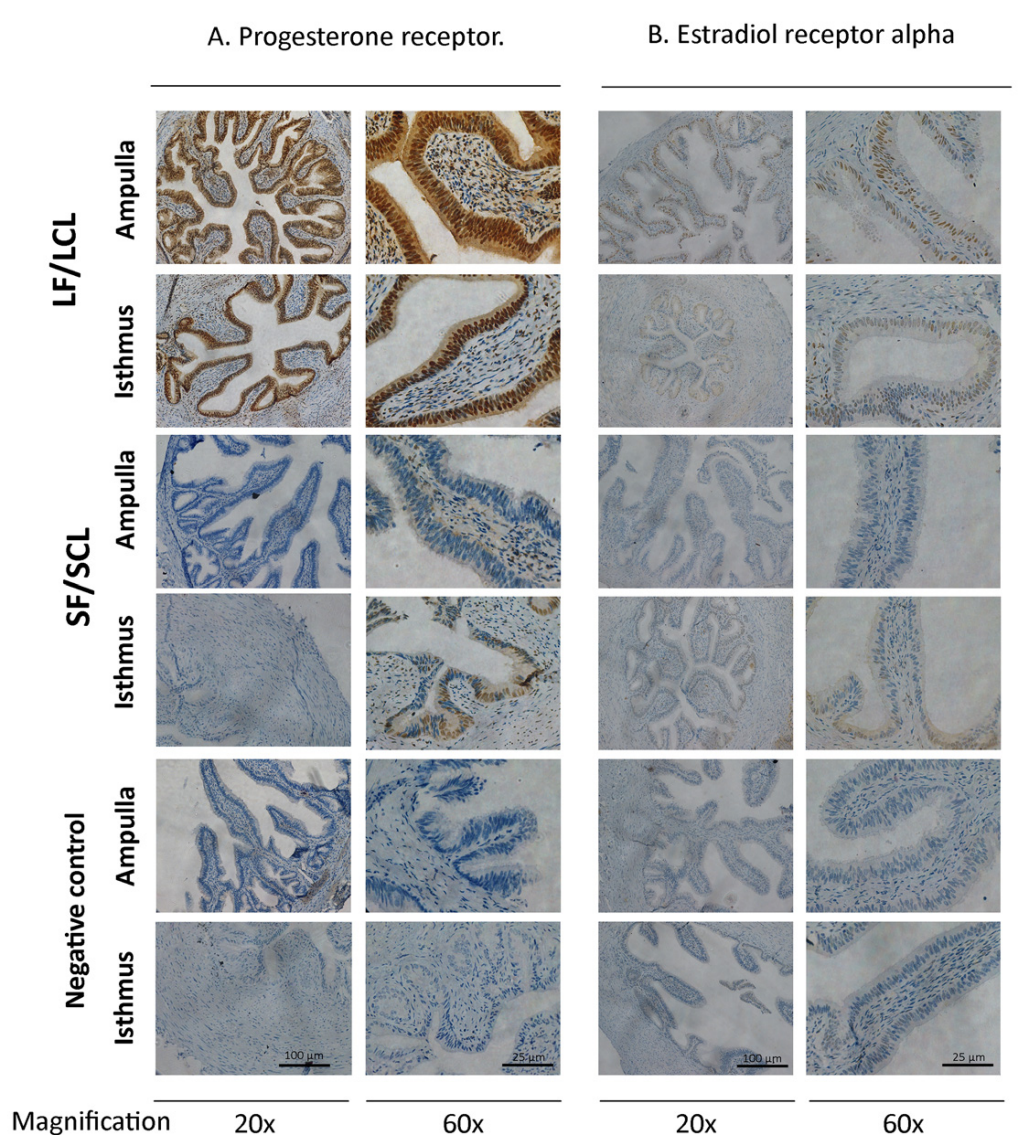
Localization of Progesterone receptor (PGR) and Estradiol receptor alpha (Er alpha) in the bovine oviduct by immunohistochemistry. Representative images of PGR and ER alpha immunohistochemical localization in the ampulla and the isthmus of LF/LCL and SF/SCL groups at Day 4 of the estrous cycle (n = 5 per group). Original magnification: 20x (scale bar: 100 μm) and 60x (scale bar: 25 μm). For an overview of PGR and ER alpha staining on samples of all animals see S1 and S2 Figs.

There was no region, treatment or interaction effect on the expression of *PGR* or *PGRMC-2* genes (Table 2). Using 5 animals per group, immunohistochemistry was performed using an ERa antibody and a PGR antibody that does not discriminate the PGR isoforms. The PGR immunostaining pattern was different between LF-LCL and SF-SCL animals (Fig 3; S1 Fig). Animals of the LF-LCL group showed similar strong immunostaining in the ampullar and isthmus LE, a weak staining in the SC, a mild staining on the ML, and no positive immunostaining in the SM (Table 3). In contrast, isthmus SF-SCL samples presented a mild immunostaining in LE, a weak staining in SC and ML, and no positive staining in the SM. However, SF-SCL ampullary samples had weak staining in SC and ML, and no staining in LE and SM (just one of five animals of the SF-SCL group showed moderate staining in the LE). *ESR1* expression was greater in the ampulla than in the isthmus (Fold Change: 1.65; P<0.001), but it was similar between treatments; no region or treatment effects were found for *ESR2* expression (Table 2). The immunostaining of the ERa showed also differences between groups and between regions (Table 3). There was a stronger immunostaining in the ampulla and isthmus in the LF-LCL group than the SF-SCL group (Fig 3; S2 Fig). Based on immunohistochemical data, greater abundance of PGR and ERa for the LF-LCL group indicates a greater availability of receptors and, possibly, sex steroids stimulated signaling, both in the ampulla and isthmus in this group of animals. Interestingly, protein staining intensities were not consistent with respective PGR and ESR1 transcript abundances.

**Table 2:** Relative Abundance of transcripts of different genes in the ampulla and isthmus of LF/LCL and SF/SCL Animals (n = 14).

Gene	LF/LCL		SF/SCL		*P* Value		
	Ampulla	Isthmus	Ampulla	Isthmus	Group	Region	Interaction
ANGPT2	0.225 ± 0.03	0.291 ± 0.092	0.230 ± 0.040	0.221 ± 0.069	0.61	0.87	0.77
ANGPT4	0.095 ± 0.016	0.238 ± 0.097	0.085 ± 0.017	0.221 ± 0.055	0.84	˂ 0.01	0.63
CADM3	0.496 ± 0.089	0.103 ± 0.055	0.257 ± 0.122	0.108 ± 0.027	0.96	0.45	0.57
C-MET	0.069 ± 0.007	0.083 ± 0.012	0.052 ± 0.016	0.064 ± 0.019	0.04	0.01	0.31
CTGF	0.192 ± 0.018	0.197± 0.104	0.164 ± 0.044	0.198 ± 0.048	0.58	0.41	0.83
CTSS	0.501 ± 0.061	0.305 ± 0.042	0.404 ± 0.074	0.456 ± 0.046	0.29	0.96	0.72
CXCR4	0.021 ± 0.000	0.016 ± 0.000	0.002 ± 0.000	0.001 ± 0.000	0.02	<0.001	0.28
EDN1	0.170 ± 0.013	0.211 ± 0.029	0.121 ± 0.008	0.444 ± 0.082	0.02	<0.001	0.72
ESR1	0.588 ± 0.057	0.326 ± 0.034	0.504 ± 0.040	0.335 ± 0.055	0.38	<0.001	0.48
ESR2	0.398 ± 0.07	0.294 ± 0.092	0.327 ± 0.076	0.22 ± 0.032	0.77	0.48	0.51
HPSE	0.338 ± 0.068	0.071 ± 0.050	0.256 ± 0.115	0.019 ± 0.003	0.03	<0.001	0.76
HSPA1A	0.290 ± 0.069	0.111 ± 0.036	0.367 ± 0.085	0.199 ± 0.128	0.88	0.01	0.96
OVGP1	0.118 ± 0.052	0.012 ± 0.010	0.233 ± 0.096	0.016 ± 0.010	0.62	<0.001	0.56
PCNA	0.949 ± 0.209	0.913 ± 0.174	0.622 ± 0.112	1.144 ± 0.144	0.30	0.13	0.33
PDGF	0.044 ± 0.003	0.032 ± 0.007	0.034 ± 0.007	0.028 ± 0.006	0.01	0.04	0.68
PGR	0.308 ± 0.030	0.311 ± 0.036	0.371 ± 0.064	0.317 ± 0.031	0.83	0.49	0.34
PGRMC2	0.479 ± 0.014	0.346 ± 0.078	0.450 ± 0.020	0.445 ± 0.043	0.26	0.24	0.46
RGS20	0.370 ± 0.101	0.220 ± 0.097	0.272 ± 0.077	0.291 ± 0.007	0.02	0.05	0.69
TGFB2	0.499 ± 0.060	0.520 ± 0.062	0.581 ± 0.032	0.331 ± 0.042	0.12	0.11	0.83
TGFB3	0.154 ± 0.02	0.572 ± 0.092	0.218 ± 0.046	0.36 ± 0.036	0.05	<0.001	0.98
TGFBR1	0.174 ± 0.052	0.291 ± 0.140	0.295 ± 0.107	0.181 ± 0.130	0.83	0.84	0.69
TGFBR2	0.163 ± 0.017	0.204 ± 0.104	0.071 ± 0.029	0.127 ± 0.044	0.60	0.01	0.66
VCL	0.086 ± 0.009	0.379 ± 0.060	0.016 ± 0.010	0.307 ± 0.042	0.03	0.01	0.12

Relative expression level normalized against Normalization factor for the most stable genes (GAPDH and PPIA) provided by geNorm.

**Table 3:** Subjective scores* of PGR staining in ampulla and isthmus on day 4 of the cycle of LF/LCL (n = 5) and SF/SCL (n = 5) animals.

Marked Protein	Region	Group	Mucous Membrane		Muscular Layer	Serous membrane
			Luminal epithelia	Stromal cells		
PGR	Ampulla	LF/LCL	+++	+	+	0
	Isthmus		+++	+	++	0
	Ampulla	SF/SCL	0	0	0	0
	Isthmus		++	+	+	0
ESR1	Ampulla	LF/LCL	++	++	+	0
	Isthmus		+++	+++	+	0
	Ampulla	SF/SCL	+	0	0	0
	Isthmus		++	+	0	0

* The staining intensity was evaluated using a four-point scoring scale: Strong (+++), mild (++), weak (+) or no staining (0).

### RNA-Seq analyses

#### Ampulla RNAseq

**Fig 4:**
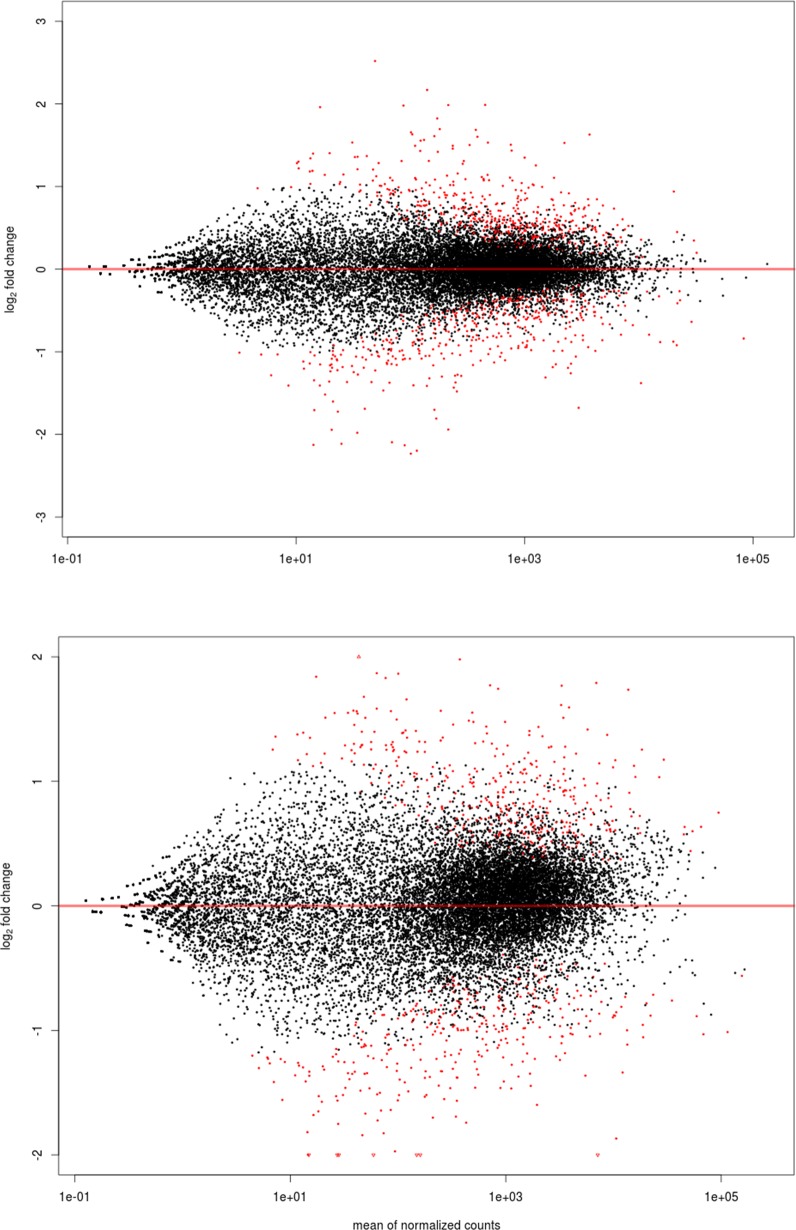
MA Plot showing ampulla (A; n = 5 samples) and isthmus (B; n = 6 samples) gene expression of LF/LCL and SF/SCL groups, in terms of the differentially expressed genes (p < 0.001).

**Fig 5:**
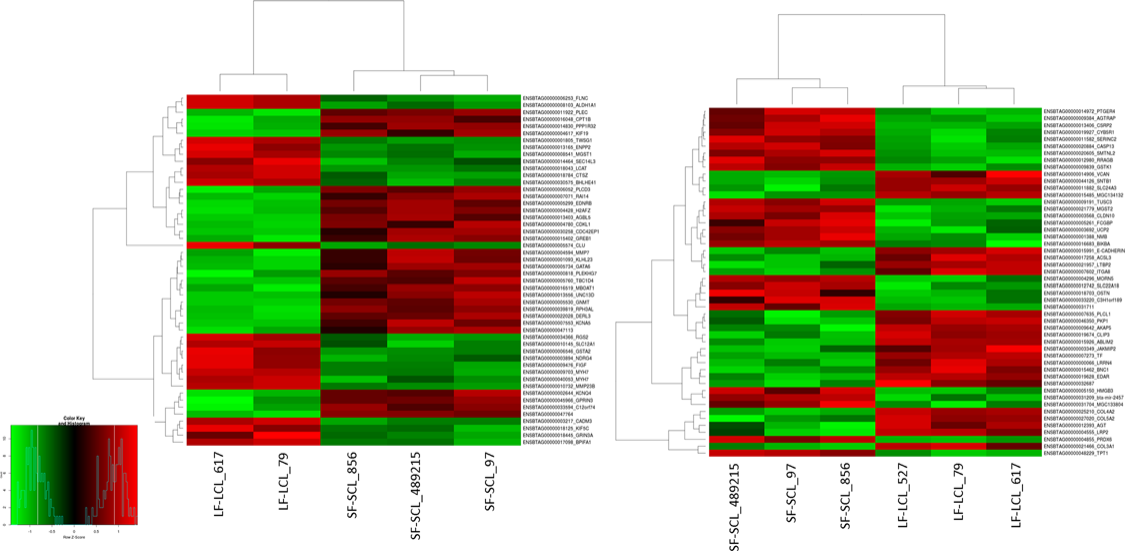
Heat map constructed by clustering of the 50 most differently expressed genes between LF/LCL and SF/SCL in the ampulla (n = 5) and the isthmus (n = 6). The colors in the map display the relative standing of the reads count data; GREEN indicates a count value that is lower than the mean value of the row while red indicates higher than the mean. The shades of the color indicate distance from each data point to the mean value of the row. Columns represent individual samples of LF/LCL and SF/SCL.

The ampulla RNAseq produced a total of ~157 million reads with an average of 30 million reads for each sample. Three biological replicates were analyzed for each group with the reads ranging from 29–36 million per sample after filtering (S1 Table). After using HTSeq-count, approximately ~60% of the total reads uniquely mapped to the UMD 3.1 reference genome, excluding also reads that aligned ambiguously. There were approximately 10% of not-uniquely mapped reads, 15% non-specifically mapped reads, and 15% unmapped reads. Only the uniquely mapped reads were considered in the analysis. One sample of the LF-LCL group was discarded from the RNA-Seq analyses, due to inadequate library quality resulting in few reads. In order to categorize the genes with different level of expression, a multiphasic graph was obtained by plotting the log2 transformed baseMean values versus all expressed genes from the *Bos taurus* genome. According to the phases in the graph, gene expression values were categorized into three groups: high (≥ 1000 reads normalized or baseMean), medium (≥ 15 to 1000 baseMean), and low (< 15 baseMean) expression genes. There were 3,379 (25.6%) highly expressed genes, 10,456 (67.4%) medium expressed genes, and 1,107 genes (7%) with low expression. There were 2,123 (13.6%) and 2,162 (13.9%) highly expressed genes in SF-SCL and LF-LCL, and 449 (2.8%) and 485 (3.1%) lowly expressed genes in SF-SCL and LF-LCL, respectively. After applying the variance and minimal value of baseMean filtering, a total of 15,815 genes were included on the differential expression analysis (see MAplot on Fig 4A). A total of 692 out of the 15,815 analyzed genes showed differential expression, of which 325 and 367 were up-regulated in the ampulla of LF-LCL and SF-SCL samples, respectively. Differentially expressed genes, their respective Log2fold-changes and phenotype expression profiles averages are listed on the (S2 Table). Clustering analysis clearly separated the overall transcriptome signatures of the two groups indicating distinct tissue-specific characteristics of the expression profiles (Fig 5A). All reads sequences were deposited in the Sequence Read Archive (SRA) of the NCBI (http://www.ncbi.nlm.nih.gov/sra/; S3 Table), and an overview of this data has been deposited in NCBI’s Gene Expression Omnibus (GEO) and is accessible through GEO Series accession number GSE65681.

Additionally, using an expanded number of individuals (n = 7 per group), we validated the differential expression data for 23 genes by qPCR (S4 Table). Validation showed agreement of the expression patterns of the RNA-seq results and the qPCR results.

#### Isthmus RNAseq

The isthmus RNAseq produced a total of ~451 million reads with an average of 75 million reads for each sample. Three biological replicates were analyzed for each group with the reads ranging from 63–80 million per sample after filtering (S1 Table). After using HTSeq-count, approximately ~60% of the total reads uniquely mapped to the UMD 3.1 reference genome and there were approximately 10% of not-uniquely mapped reads, 15% non-specifically mapped reads, and 15% unmapped reads. Only the uniquely mapped reads were considered in the analysis. As in the ampulla RNAseq, a multiphasic graph was obtained and gene expression values were categorized into three groups: high (≥ 1000 reads normalized or baseMean), medium (≥ 15 to 1000 baseMean), and low (< 15 baseMean) expressed genes. There were 6,212 (28%) highly expressed genes, 8,935 (35.8%) medium expressed genes and 9,759 genes (36.2%) with low expression. There were 2,059 (12.9%) and 413 (2.5%) highly expressed genes in SF-SCL and LF-LCL, and 10,268 (41.7%) and 11,700 (47.5) % lowly expressed genes in SF-SCL and LF-LCL, respectively.

After applying the variance and minimal value of baseMean filtering, a total of 15922 genes were included on the differential expression analysis (see MAplot on Fig 4B). A total of 590 out of the 15,922 analyzed genes showed differential expression, of which 274 and 316 were up-regulated in the isthmus of LF/LCL and SF/SCL samples, respectively. Differentially expressed genes, their respective Log2fold-changes and phenotype expression profiles averages are listed on the (S5 Table). Clustering analysis clearly separated the overall transcriptome signatures of the two groups indicating distinct tissue-specific characteristics of the expression profiles (Fig 5B). In addition, the reads sequences were deposited in the SRA (S3 Table), and the reads count was deposited in GEO under the same series accession number as that of the ampulla data. Validation of RNA-Seq data by qPCR was accomplished for 23 isthmus genes using an expanded number of animals (n = 7/group; S6 Table).

### Functional enrichment analysis of RNA-Seq results

Using the ampulla RNAseq data, 43 GO terms were enriched on the LF-LCL samples (S7 Table), while only 29 were enriched on SF-SCL samples (p-adjusted < 0.1; S8 Table). Likewise, using the isthmus RNAseq data, 96 and 26 GO terms were enriched in LF-LCL and SF-SCL groups, respectively (S9 and S10 Tables).

The clustering of GO terms with at least three common genes is presented in Table 4. Using these criteria, terms associated with immune cell activation, vacuole and lysosome, homeostasis, cytoskeleton and extracellular matrix (ECM), chemokine signaling, plasma lipoprotein, and GTP binding were presented in the LF-LCL ampulla. In the SF-SCL ampulla, terms associated with cell development, differentiation and motility, and voltage-gated channel activity were found. In the LF-LCL isthmus samples, terms related with cellular matrix, morphogenesis and cell proliferation, homeostasis, ion transport, and glycoprotein were founded; while in the SF-SCL isthmus samples, the enriched terms were protein kinase, apoptosis, protein folding, cellular metabolism, antigen processing and presentation, nucleotide biosynthetic process, and lipid biosynthetic process.

**Table 4:** Group of the repeated GO terms in enriched processes. Using the gene enrichment data, similar GO terms were grouped when having 3 or more common genes.

Region and Group	Enriched process	Genes
Ampulla LF/LCL	Immune cell activation	*BCL10*, *CD3E*, *CENPF*, *IL2RG*, *SASH3*
	Vacuole and lysosome	*ACP2*, *BCL10*, *GJA1*, *CTSS*, *PRDX6*, *C1orf85*, *FUCA1*, *CTSZ*, *GBA*
	Homeostasis	*MT3*, *GLRX5*, *PRDX6*, *NXN*, *NAB2*, *CAV1*, *SELT*, *PLN*, *SH3BGRL3*, *CD52*
	Cytoskeleton and extracellular matrix	*PFN1*, *MAPT*, *ARPC4*, *MYH7*, *PDLIM7*, *ACTB*, *PBXIP1*, *CTNNA1*, *ACTR2*, *CDC42SE1*, *ACTN1*, *S100A8*, *FGA*, *TWSG1*, *LOXL1*, *LAMA4*, Uncharacterized protein (ENSBTAG00000009703), *ELMO1*, *PLS3*, *CTNNA1*, *VCL*, *FIGF*, *MMP23B*, *LGALS1*, *SPOCK1*, *LCAT*, *LOXL1*, *ANGPTL1*
	Chemokine signaling	*VAV1*, *GNAI2*, *CLDN4*, *ACTB*, *CTNNA1*, *PLCG1*, *CXCR4*, *VCL*, *ACTN1*, *ELMO1*, *PAK1*, *GNG11*, *GNG10*, *CXCL10*, *CXCL14*
	Plasma lipoprotein	*LCAT*, *SAA3*, *SAA1*
	GTP binding	*GNAI2*, *RIT1*, *SEPT11*, *GEM*, *MRAS*, *RASEF*, Uncharacterized protein (ENSBTAG00000037510), *GIMAP7*, *GBP4*, *TUBA1B*, *RASL11B*
Ampulla SF/SCL	Cell development, differentiation and motility	*PAX6*, *ABI2*, *EDNRB*, *IL16*, *EFNA5*, *KDR*, *TGFB2*, *EFNB1*, *GATA6*, *VEZF1*
	Voltage-gated channel activity	*KCNRG*, *KCTD1*,*KCNJ11*, *CACNB2*, *KCNQ4*, *KCNA5*, *KCNAB1*, *SLC4A4*, *SLC38A11*, *SCO2*, *LASP1*
	Ephrin receptor binding	*EFNB1*, *TIAM1*
	Adherens junction	*CTNNA2*, *ABI2*, *CXADR*, *LASP1*
Isthmus LF/LCL	Cellular matrix	*H18*, *E-CADHERIN*, *CDH19*, *FN1*, *CCDC80*, *VCAN*, *BMP4*, *COL1A1*, *COL3A1*, *COL4A1*, *LUM*, *EPDR1*, *AGT*, *MGC142792*, *PI16*, *COLEC12*, *CHRNA1*, *MMP14*, *SV2A*, *LOX*, *ADAMTS2*, *ITGA11*, *DSG3*, *COL18A1*, *LAMC1*, *FBLN1*, *COL4A6*, *COL4A2*, *COL6A2*, *COL8A1*, *C1QTNF3*, *COL5A3*, *ASPN*, *ADAMTS4*, *IGF-I*, *SMOC2*, *MMP24*, *LTBP1*, *PCD*, *NGFR*, *ADAM23*
	Morphogenesis and cell proliferation	*MMP14*, *AGT*, *IGF-I*, *BMP4*, *ADAMTS2*,*LOX*, *HNF1A*, *COL1A1*, *NGFRCOL8A1*, *PDGFD*, *C-MET*,*BMI1*, *NGFR*, *F2R*, *IL1B*, *KCNMA1*, *E-CADHERIN*, *FOXL2*, *C-MET*
	Homeostasis	*F2R*, *SELV*, *HNF1A*, *CHRNA1*, *KCNMA1*, *TF*, *SV2A*, *IGF-I*, *CACNA1G*, *ITPR1*
	Ion transport	*CAPN11*, *LTBP2*, *EPDR1*, *LTBP1*, *NPNT*, *DSG3*, *MMP14*, *CDH19*, *MMP24*, *FKBP9*, *FKBP10*, *FBLN7*, *MYLK*, *E-CADHERIN*, *VCAN*, *ITPR1*, *FBLN1*, *CAPN1*, *PCDH18*, *KCNMA1*, *ACTN3*, *PLCB1*, *SMOC2*, *C1S*, *PLCL1*, *COLEC12*, *F2R*, *GRIN2D*, *P2RX3*, *ATP2B3*, *CACNA1G*, *CHRNA3*, *GABRA1*, *CHRNA1*, *CLCN4*, *CLIC4*, *GRIA3*, *KCNA4*, *TF*, *MOCOS*, *PLOD1*, *MGC142792*, *RGNEF*, *ADAMTS4*, *BNC1*, *ENPP1*, *BMI1*, *CLIC4*, *NMNAT1*, *TRIM9*, *JAZF1*, *PKLR*, *PPM1L*, *ADAM23*, *ADAMTS2*, *LOX*, *RORB*, *SLC12A2*
	Glycoprotein	*TFPI2*, *PLOD1*, *TF*, *ADAMTS4*, *LTBP2*, *KCNA4*, *PROCR*, *EPDR1*, *CHRNA3*, *GABRA1*, *GPM6A*, *PI16*, *SV2A*, *C7*, *FN1*, *LRRC8C*, *HYAL1*, *FKBP9*, *FKBP10*, *E-CADHERIN*, *VCAN*, *EDNRA*, *ASPN*, *COL3A1*, *C-MET*, *F2R*, *PCDH18*, *COL1A1*, *ACPP*, *CHRNA1*, *ADAMTS2*, *PTGDR*, *C1S*, *LOX*, *COLEC12*, *ANGPTL1*, *COL4A1*, *LUM*, *BMP4*, *MYOC*, *IL1B*, *CST9L*, *SRPX2*, *COL4A2*, *IGF-I*, *COL1A1*, *CHRNA3*, *KCNMA1*, *AGT*
Isthmus SF/SCL	Protein kinase	*ROPN1*, *SPA17*, *ROPN1L*, *CABYR*
	Apoptosis	*TMBIM6*, *BNIPL*, *WDR92*, *CLU*, *TNS4*, *ITM2B*, *CASP8*, *CASP13*, *IFI6*
	Protein folding	*HSPA8*, *DNAJB1*, *HSPH1OSGEP*, *MME*, *PRDX6*, *RNF114*, *CBLC*
	Cellular metabolism	*GGT6*, *GSTK1*, *MGST1*, *MGST2*, *VNN1*, *BLVRA*, *NADSYN1*, *HS3ST5*, *SLC1A3*, *PCYOX1L*, *QSOX1*
	Antigen processing and presentation	*HSPA8*, *BOLA*, *HSPA1A*, *HSP90AA1*, *IFI30*, *HLA-DMB*, *FCGRT*, *AZGP1*
	Nucleotide biosynthetic process	*GUCY1B1*, *HPRT1*, *ATP5F1*, *ATP5S*, *ADSS*, Uncharacterized protein (ENSBTAG00000005217), *GUCY2C*, *NADSYN1*
	Lipid biosynthetic process	*ALG10*, *THG1L*, *TPST2*, *MBOAT7*, *HPRT1*, *MGST1*, Uncharacterized protein (Uncharacterized protein (ENSBTAG00000005217), *CEPT1*, *OAT*, *MGST2*, *GGT6*, *GSTK1*, *CKMT1*, *PIGW*, *B3GNT8*, *DDR1*, *MAPK13*, *HS3ST5*, *ACAA2*, *CPT1B*, *PAK1*, *LPCAT4*, *AS3MT*, *ACAA2*

It is noteworthy that both regions in the LF-LCL group had enriched ECM and homeostasis GO terms. The ECM is composed of fibrous collagen and non-collagen proteins (glycoproteins and proteoglycans), which bind to proteins on cell surface promoting a variety of cellular responses including survival, proliferation, adhesion, and migration [49, 50]. In the present study, both oviduct regions of the LF-LCL group showed enrichment of the ECM pathways. This includes increased (P adjust ˂ 0.05; log2 fold change ranging from -1.64 to 0.375) expression of ECM constituent genes such as collagens (*COL1A1*, *COL3A1*, *COL4A1*, *COL4A6*, *COL4A2*, *COL6A2*, *COL8A1*, *COL5A3*), versican (*VCAN*), actin beta (*ACTB*), actinin alpha 1 (*ACTN1*), integrin alpha 11 (*ITGA11*), fibronectin 1 (*FN1*), and vinculin (*VCL*). It also includes remodeling enzymes such as Matrix metalloproteinases (*MMP14*, *MMP24*), ADAM Metallopeptidase Domain 23 (*ADAM23*), ADAM metallopeptidase with thrombospondin type 1 motif 2 (*ADAMTS2*) and motif 4 (*ADAMTS4*), desmoglein 3 (*DSG3*), lysyl oxidase (*LOX*), profilin 1 (*PFN1*), and fibrinogen alpha chain (*FGA*). This suggests that in those animals an active process of ECM remodeling was occurring [49].

Homeostasis is the process by which cells maintain the optimal internal conditions to support their correct function. Both regions of the LF-LCL group have enriched expression of genes associated with homeostatic processes (P adjust ˂ 0.05; log2 fold change ranging from -1.97 to 0.-7225). Enriched genes are involved in the control of important cell processes such as Redox systems {peroxiredoxin 6 (*PRDX6*), nucleoredoxin (*NXN*), selenoprotein V (*SELV*), and selenoprotein T (*SELT*)}, Ca and K transport {potassium large conductance calcium-activated channel subfamily M alpha member 1 (*KCNMA1*), calcium channel, voltage-dependent, T type, alpha 1G subunit (*CACNA1G*), and inositol 1,4,5-trisphosphate receptor, type 1 (*ITPR1*)}, iron binding, and transport {transferrin (*TF*)}. These findings could indicate that such homeostatic processes are more prevalent in animals manipulated to ovulate larger follicles.

Additionally, when comparing the two group’s isthmus region, it is noteworthy that the LF-LCL isthmus is apparently signaling for cellular proliferation, while the SF-SCL isthmus tissue is signaling for apoptosis. In the LF-LCL animals, important proliferating genes were up-regulated {insulin-like growth factor 1 (*IGF1*) and bone morphogenetic protein 4 (*BMP4*)}, while in the SF/SCL isthmus, pro-apoptotic genes were up-regulated [caspase 8 (*CASP8*) and 13 (*CASP13*)].

### Treatment and region effects on the expression of candidate genes

The effects of group and region on the expression of candidate genes were analyzed (Table 2) using 7 animals per group. Genes were selected from functional categories of interest. There was no interaction (Group * Region) effect on the expression of any of the 15 genes studied. CXCR4, HPSE, PDGF and RGS20 were more expressed in LF-LCL group and in the ampulla, while C-MET, TGFB3 and VCL were more expressed in the LF-LCL group and in the isthmus. EDN1 was more expressed in the SF-SCL group and in the isthmus.

*OVGP1* and *HSP1A* were more expressed in the ampulla while *ANGPT4 and TGFBR2* were more expressed in the isthmus.

Finally, there was no region or treatment effect on the expression of genes involved in cell proliferation pathway (*CTGF*, *TGFB2*, *TGFBR1* and *PCNA*), one extracellular matrix gene (*CADM3*), one angiogenic gene (*ANGPT2*) and the *CTSS* gene.

## Discussion

The oviduct provides the environment for oocyte transport, fertilization, and early embryonic development. Function of the oviduct is primarily regulated by periovulatory sex steroids hormones [17]. Timing and magnitude of changes in sex steroids concentrations around ovulation are associated with reproductive tract receptivity to the embryo and, ultimately, fertility. However, information about the cellular and molecular needs of the oviductal tissue in the early post ovulation period is lacking. Thus, the hypothesis was that the oviductal transcriptome around D4 post-ovulation is regulated by the endocrine profile in such a way that it fulfills the needs of the early developing embryo. We aimed to determine the influence of different periovulatory endocrine milieus on the ampulla and isthmus transcriptome. Using a P4/E2 based protocol to synchronize ovulations, we manipulated Nelore cows to ovulate small (SF-SCL) or large (LF-LCL) follicles that resulted in different proestrus E2 and metestrus P4 concentrations. On D4, animals were slaughtered and oviduct was collected and studied for the effect of different periovulatory endocrine milieus on gene expression. Our study was the first to identify molecular pathways using RNAseq technology in oviductal samples on D4 and in response to distinct periovulatory endocrine profiles.

In this study, we showed clearly that the oviductal transcriptome is under sex-steroid control. However, an added step of complexity is the auto-regulatory effects of E2 and P4 on their receptors. Regulation was apparently both at the transcript and protein levels, which were not coupled. The results presented herein are in line with previous data showing that PGR and ERa expression can be up-regulated in response to an E2 stimulus, which is mediated by complex transcription and translation mechanism [51, 52]. Using an in vitro rat endometrial cell culture system, it was demonstrated that after an initial E2 stimulus, *PGR* mRNA accumulation was detected after 6 h and continued to accumulate until 18 h. Remarkably, this slow and gradual *PGR* transcription rate did not parallel binding of E2 to ER, which was maximized within 30 min [51]. Furthermore, results from in vivo experiments indicated that oviductal *ESR1* and *PGR* mRNA levels increased around estrus, but increases in abundance of ERa and PGR protein were only detected in the early luteal phase [11]. An integrated interpretation of the steroid / steroid receptor protein changes reported here is that there was a prominent P4 signaling to the oviduct of cows in the LF-LCL group. It is tempting to speculate that at least part of the transcriptome changes associated with the treatment groups were due to such differences in signaling.

It is important to remember that our experimental model cannot assess the possible effects of the presence of sperm or a developing embryo on the oviductal gene expression. Others have reported that these structures could modulate the gene expression of oviductal epithelial cells [5, 53]. Therefore, future studies should be performed to assess whether cows ovulating follicles of different sizes, respond differently to the stimuli caused by sperm cells and embryos.

To gain insight on the functional role of molecular and cellular changes induced by treatments in the oviductal tissues, we took a two-pronged approach. First, we employed transcriptomics and bioinformatics tools to define the pathways and processes most significantly affected between groups and regions. Second, we studied the expression of genes selected from the literature because of their known roles on oviduct function. Regarding the first approach, enrichment analysis successfully separated the two treatment groups in the context of particular oviduct regions. Among the many enriched processes and pathways detected, we will highlight ECM remodeling and cellular homeostasis because they were commonly enriched processes between isthmus and ampulla of LF-LCL animals, and cell proliferation and apoptosis because they were opposite processes when comparing the isthmus between groups.

The ECM interacts with cells in order to both provide mechanical support and molecular signals necessary for the regulation of many processes vital to the cell, such as adhesion and migration [54], proliferation [55], angiogenesis [49], among others. The ECM comprised of collagen fibers, viscous proteoglycans and adhesive extracellular proteins, which bind proteoglycans, collagen fibers and receptors in the cell surface [50, 56]. The ECM is under constant remodeling, as cells constantly degrade and resynthesize the ECM components to promote rapid changes in the microenvironment [49]. All the ECM protein components are subject to degradation and modification, and there are two families of metalloproteinases, including matrix metalloproteinase (MMP) and a disintegrin and metalloproteinase with thrombospondin motifs (ADAMTS). Both are specialized in degrading the ECM [57, 58]. At the beginning of the remodeling process, there is an up-regulation in the expression of genes that code for both ECM constituents and ECM remodeling enzymes [57, 58]. In the present study, both oviduct regions of the LF-LCL showed enrichment in the expression of genes related to ECM remodeling pathways, including increased expression of matrix component genes (e.g., collagens) and remodeling enzymes (e.g., MMPs and ADAMs). Also, when the *HPSE* gene was evaluated by qPCR, it was more expressed in the LF-LCL groups. Matrix reorganization is closely associated with tissue morphogenesis and cell proliferation. Both of these processes are enriched in the LF-LCL isthmus based on the expression of *MMPs*, *ADAMS*, and *IGF1* for morphogenesis, and *BMP4*, *NGFR*, *BMI1*, and *C-MET* for cell proliferation. Regarding proliferation, it is noteworthy that remodeling uncouples a variety of growth factors linked to specific ECM proteins. For example, IGF1, FIGF, FGF, and VEGF are uncoupled from the ECM during tissue injury in order to promote healing [57, 59]. In addition to the potential of increasing available growth factors via increased remodeling, transcript abundance of *IGF1* and *FIGF* are both up-regulated in the LF-LCL ampulla and isthmus, respectively. In the oviduct, synthesis and secretion of these growth factors could result in increased availability in the lumen for stimulation of embryo growth and development [37]. Indeed, addition of these factors in vitro improved embryo production [60, 61].

We speculate that higher fertility associated with the LF-LCL cows could be at least partly explained by a favorable oviductal environment that results from a balanced array of growth factors. However, more studies must be conducted in order to establish if there is agreement between transcript and protein changes associate with the LF-LCL animals, and, if an active ECM remodeling process is going on, as indicated by transcript abundance changes.

The homeostatic processes enriched in the ampulla and isthmus of animals in the LF-LCL group include genes controlling cell-Redox systems (e.g., *PRDX6*, *NXN*, *SELV*, and *SELT*), Ca and K transport (*KCNMA*, *CACNA1G*, *ITPR1*), and iron binding and transport (*TF*). Proteins with antioxidant properties reduce cellular peroxides and protect cells from reactive oxygen species-mediated damage and death. For example, the overexpression of *PRDX6* gene in *HEPA1-6* cells was associated with resistance to peroxide-induced cell death [62]. Also, the presence of reactive oxygen species in vitro could decrease the fertilization and embryo development [63]. It is possible that a more adequate redox environment in the LF-LCL group prevents embryo cellular damage and cell death. Indeed, work from our group suggests that similar redox characteristics were associated with the uterine environment on D7, comparing LF-LCL and SF-SCL cows [64].

The net result of the up-regulation of ion transporter genes could be an increase in Ca^2+^ influx at the cell level. Ca^2+^ is a universal second messenger and is involved in many cellular processes including cell proliferation, migration, and secretion. It has been long known that the influx of external Ca^2+^ into the cell is needed to induce cell proliferation and cell cycle progression in mammalian cells [65]. These results are consistent with the effects expected for ECM remodeling mentioned above. Indeed, cellular process of apoptosis and proliferation are components of growth regulation in normal epithelia [66]. Here, we reported that cows in the LF-LCL showed increased abundance of genes related to proliferation, while, in contrast, genes related to apoptotic signaling were more abundant in tissues from SF-SCL group. Another important role of increased Ca^2+^ and K signaling is smooth muscle contraction and this may be important for embryo transport to the uterus on D4 after estrus.

One final aspect that deserves attention is oviductal secretions. In that regard, animals of the LF-LCL group showed up-regulation of TF. TF is a protein that binds iron for transport into the cells and also serves as a detoxifying agent, by sequestering metals from the medium. TF has been used in order to improve blastocyst yield of serum-free culture systems [67, 68] and has been directly associated with endometrial embryo nutrition because it functions as an iron transporter to the lumen and ultimately to the conceptus [69, 70].

In summary, our study identified, for the first time, a series of functional characteristics of the oviduct that are regulated by the periovulatory sex steroid milieu and that potentially affect early embryo development and, ultimately, fertility. Characteristics include tissue morphology changes (e.g., ECM remodeling), cellular changes (e.g., proliferation and apoptosis), and secretion changes (e.g., growth factors, ions, and metal transport). Future studies are warranted to investigate specific pathways in detail. In conclusion, differences in the periovulatory sex steroid milieu lead to different oviductal gene expression profiles that could modify the oviductal environment and affect embryo survival and development.

## Supporting Information

S1 Fig Localization of PGR in the bovine oviduct by immunohistochemistry: images of PGR immunohistochemical localization in the ampulla and the isthmus of LF/LCL and SF/SCL animals at Day 4 of the estrus cycle. In each column, ampulla and isthmus samples from individual animals of the LF-LCL and SF-SCL groups are shown. Original magnification 20x, Scale bar 100 μm (n = 5 per group).
(PDF)

S2 Fig Localization of ERalpha in the bovine oviduct by immunohistochemistry: images of ERalpha immunohistochemical localization in the ampulla and the isthmus of LF/LCL and SF/SCL animals at Day 4 of the estrus cycle. In each column, ampulla and isthmus samples from individual animals of the LF-LCL and SF-SCL groups are shown. Original magnification 20x, Scale bar 100 μm (n = 5 per group).
(PDF)

S1 Table N raw reads, N reads post filtering, Mapped reads, uniquely mapped reads and percentage of mapped reads obtained in the RNAseq of ampulla and isthmus samples. (DOCX)

S2 Table Differentially expressed genes in ampulla samples (n = 5; p-adjusted < 0.1), respective expression profiles and Log2fold-changes for both treatments, LF/LCL and SF/SCL. (DOCX)

S3 Table Bio-project, Bio-sample, Experiment and Run accession numbers of the Raw reads resulted from the RNAseq of ampulla and isthmus samples in the SRA data base. (DOCX)

S4 Table log2 Fold change and P value of ampulla gene expression in LF/LCL and SF/SCL animals. **Validation of RNAseq gene expression data by qPCR.** qPCR data was analyzed using the same RNAseq animals (n = 5) and using 7 animals for each group (n = 14).
(DOCX)

S5 Table Differentially expressed genes in isthmus samples (n = 6; p-adjusted < 0.1). Respective expression profiles and Log2fold-changes for both treatments LF/LCL and SF/SCL. (DOCX)

S6 Table log2 Fold change and P value of isthmus gene expression in LF/LCL and SF/SCL animals. Validation of RNAseq gene expression data by qPCR. qPCR data was analyzed using the same RNAseq animals (n = 6) and using 7 animals for each group (n = 14).
(DOCX)

S7 Table Gene ontologies (GO category) of mRNA transcripts differentially expressed in day 4 Ampulla samples of the LF/LCL group. Gene ontology analysis is performed with DAVID tools (http://david.abcc.ncifcrf.gov/tools.jsp). The enrichment p-values are corrected by Benjamini's methods. GO categories are presented according to their biological process, cellular component and molecular function.
(DOCX)

S8 Table Gene ontologies (GO category) of mRNA transcripts differentially expressed in day 4 Ampulla samples of the SF/SCL group. Gene ontology analysis is performed with DAVID tools (http://david.abcc.ncifcrf.gov/tools.jsp). The enrichment p-values are corrected by Benjamini's methods. GO categories are presented according to their biological process, cellular component and molecular function.
(DOCX)

S9 Table Gene ontologies (GO category) of mRNA transcripts differentially expressed in day 4 Isthmus samples of the LF/LCL group. Gene ontology analysis is performed with DAVID tools (http://david.abcc.ncifcrf.gov/tools.jsp). The enrichment p-values are corrected by Benjamini's methods. GO categories are presented according to their biological process, cellular component and molecular function.
(DOCX)

S10 Table Gene ontologies (GO category) of mRNA transcripts differentially expressed in day 4 Isthmus samples of the SF/SCL group. Gene ontology analysis is performed with DAVID tools (http://david.abcc.ncifcrf.gov/tools.jsp). The enrichment p-values are corrected by Benjamini's methods. GO categories are presented according to their biological process, cellular component and molecular function.
(DOCX)
