# Supplementary material for: Size of the Ovulatory Follicle Dictates Spatial Differences in the Oviductal Transcriptome in Cattle
Source: PLoS One. 2015 Dec 23;10(12):e0145321. doi: 10.1371/journal.pone.0145321 (PMC4689418; doi:10.1371/journal.pone.0145321)

S1 Fig: Localization of PGR in the bovine oviduct by immunohistochemistry. Images of PGR immunohistochemical localization in the ampulla and the isthmus of LF/LCL and SF/SCL animals at Day 4 of the estrus cycle. In each column, ampulla and isthmus samples from individual animals of the LF-LCL and SF-SCL groups are shown. Original magnification 20x, Scale bar 100 µm (n=5 per group).

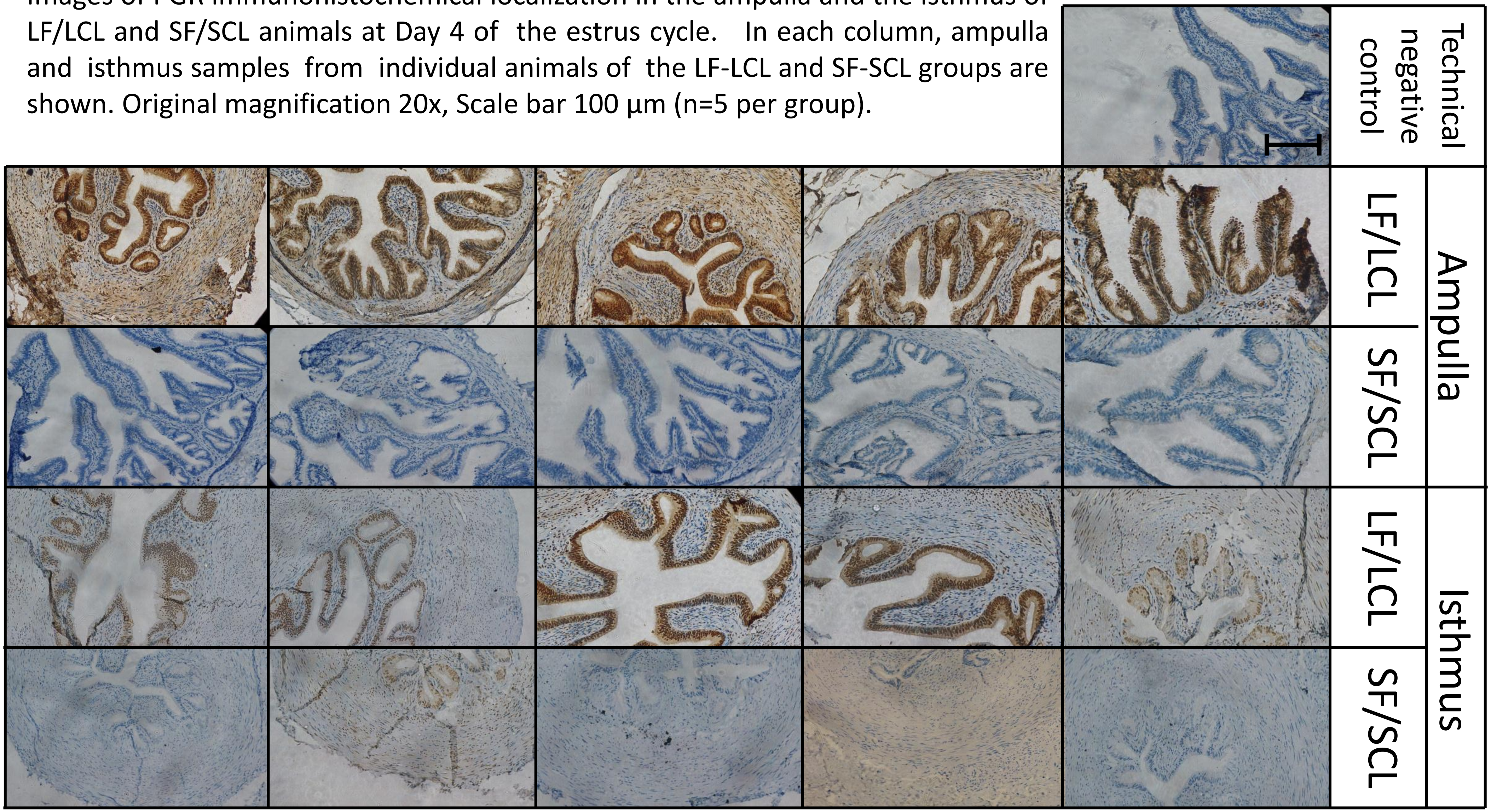

Supplement: S1 Fig — In each column, ampulla and isthmus samples from individual animals of the LF-LCL and SF-SCL groups are shown. Original magnification 20x, Scale bar 100 μm (n = 5 per group). (PDF) [file pone.0145321.s001.pdf]
