# Supplementary material for: Size of the Ovulatory Follicle Dictates Spatial Differences in the Oviductal Transcriptome in Cattle
Source: PLoS One. 2015 Dec 23;10(12):e0145321. doi: 10.1371/journal.pone.0145321 (PMC4689418; doi:10.1371/journal.pone.0145321)
Supplement: S1 Table — (DOCX) [file pone.0145321.s003.docx]

**S1 Table. N raw reads, N reads post filtering, Mapped reads, uniquely mapped reads and percentage of mapped reads obtained in the RNAseq of ampulla and isthmus samples.**

| **Tissue** | **RNAseq platform** | **Samples** | **N raw reads** | **N reads post filtering** | **Mapped reads** | **Uniquely mapped reads** | **% mapped** |
| --- | --- | --- | --- | --- | --- | --- | --- |
| Ampulla | Illumina HiScanSeq | SF/SCL 97 | 31291198 | 25264594 | 21474202 | 14277845 | 56.51 |
|  |  | SF/SCL 856 | 31307140 | 24957802 | 21306846 | 14785598 | 59.24 |
|  |  | SF/SCL 489215 | 36669350 | 29289446 | 24687466 | 19592174 | 66.89 |
|  |  | LF/LCL 79 | 29829960 | 23969368 | 20305090 | 16171541 | 67.47 |
|  |  | LF/LCL 617 | 28294892 | 22601260 | 19192220 | 13418278 | 59.37 |
| Isthmus | Illumina HiSeq 2500 | SF/SCL 97 | 63610666 | 52451000 | 40407382 | 23899117 | 77.04 |
|  |  | SF/SCL 856 | 85955492 | 71049396 | 56443006 | 34193164 | 79.44 |
|  |  | SF/SCL 489215 | 78573462 | 64863854 | 49734880 | 25472906 | 76.68 |
|  |  | LF/LCL 79 | 76198884 | 62433648 | 47935261 | 28659214 | 76.78 |
|  |  | LF/LCL 617 | 67260770 | 55345160 | 43161491 | 25652651 | 77.99 |
|  |  | LF/LCL 527 | 80395348 | 66081164 | 50931929 | 29836083 | 77.07 |
