# Supplementary material for: Size of the Ovulatory Follicle Dictates Spatial Differences in the Oviductal Transcriptome in Cattle
Source: PLoS One. 2015 Dec 23;10(12):e0145321. doi: 10.1371/journal.pone.0145321 (PMC4689418; doi:10.1371/journal.pone.0145321)
Supplement: S2 Table — (DOCX) [file pone.0145321.s004.docx]

**S2 Table. Differentially expressed genes in ampulla samples (p-adjusted < 0.1) detected by RNAseq, respective expression profiles and Log2fold-changes for both treatments, LF/LCL and SF/SCL.**

| **ENSemBLe ID** | **Gene Symbol** | **Base Mean** | **log2 Fold Change** | ***P* value** | ***P* adj** |
| --- | --- | --- | --- | --- | --- |
| Upregulated LF/LCL |  |  |  |  |  |
| ENSBTAG00000003217 | *CADM3* | 100.9931 | -2.2329 | 0.0000 | 0.0000 |
| ENSBTAG00000018125 | *KIF5C* | 114.0289 | -2.1970 | 0.0000 | 0.0000 |
| ENSBTAG00000000198 |  | 89.2411 | -2.1314 | 0.0000 | 0.0000 |
| ENSBTAG00000012640 | *S100A8* | 14.1851 | -2.1263 | 0.0000 | 0.0000 |
| ENSBTAG00000017098 | *BPIFA1* | 24.9676 | -2.1136 | 0.0000 | 0.0000 |
| ENSBTAG00000018445 | *GRIN3A* | 69.0830 | -2.0945 | 0.0000 | 0.0000 |
| ENSBTAG00000006961 | *NLRP13* | 34.3522 | -1.9797 | 0.0000 | 0.0000 |
| ENSBTAG00000021007 | *FOLR1* | 20.4935 | -1.9442 | 0.0000 | 0.0000 |
| ENSBTAG00000040053 | *MYH7* | 215.1802 | -1.9409 | 0.0000 | 0.0000 |
| ENSBTAG00000009476 | *FIGF* | 168.9003 | -1.8083 | 0.0000 | 0.0000 |
| ENSBTAG00000015293 | *NXPH3* | 23.1959 | -1.7247 | 0.0000 | 0.0001 |
| ENSBTAG00000047417 | *CYLC1* | 14.4398 | -1.7063 | 0.0000 | 0.0003 |
| ENSBTAG00000009703 | *MYH7* | 162.5399 | -1.7017 | 0.0000 | 0.0000 |
| ENSBTAG00000002779 | *GABRP* | 39.9459 | -1.6893 | 0.0000 | 0.0001 |
| ENSBTAG00000014464 | *SEC14L3* | 2977.1468 | -1.6780 | 0.0000 | 0.0000 |
| ENSBTAG00000003299 | *LPAR4* | 21.0405 | -1.6020 | 0.0000 | 0.0002 |
| ENSBTAG00000012682 | *UNC13A* | 17.9455 | -1.5174 | 0.0000 | 0.0021 |
| ENSBTAG00000010145 | *SLC12A1* | 255.7333 | -1.4801 | 0.0000 | 0.0000 |
| ENSBTAG00000033554 | *TRAPPC3L* | 58.0404 | -1.4684 | 0.0000 | 0.0004 |
| ENSBTAG00000003894 | *NDRG4* | 240.2141 | -1.4334 | 0.0000 | 0.0000 |
| ENSBTAG00000044148 | *UTS2B* | 139.6706 | -1.4130 | 0.0000 | 0.0021 |
| ENSBTAG00000010719 | *ANGPTL1* | 8.5716 | -1.4086 | 0.0001 | 0.0063 |
| ENSBTAG00000013100 | *SPAG5* | 16.1388 | -1.4083 | 0.0001 | 0.0056 |
| ENSBTAG00000016407 | *IRX6* | 106.6233 | -1.4061 | 0.0000 | 0.0023 |
| ENSBTAG00000010433 | *M-SAA3.2* | 10459.3308 | -1.3792 | 0.0000 | 0.0000 |
| ENSBTAG00000010856 | *SLC8A2* | 65.7667 | -1.3744 | 0.0000 | 0.0001 |
| ENSBTAG00000006546 | *GSTA2* | 248.8463 | -1.3716 | 0.0000 | 0.0000 |
| ENSBTAG00000044119 | *KSR2* | 45.7480 | -1.3668 | 0.0002 | 0.0088 |
| ENSBTAG00000037773 | *SERPINB7* | 27.9158 | -1.3663 | 0.0002 | 0.0089 |
| ENSBTAG00000007336 | *HCST* | 19.4957 | -1.3603 | 0.0001 | 0.0047 |
| ENSBTAG00000022971 |  | 14.3687 | -1.3450 | 0.0002 | 0.0083 |
| ENSBTAG00000012638 | *S100A12* | 146.9009 | -1.3218 | 0.0000 | 0.0000 |
| ENSBTAG00000001835 | *GJA1* | 1525.8816 | -1.3112 | 0.0000 | 0.0000 |
| ENSBTAG00000002555 | *PDZK1IP1* | 189.0674 | -1.3002 | 0.0000 | 0.0024 |
| ENSBTAG00000001176 | *LRRN1* | 279.1165 | -1.2923 | 0.0001 | 0.0071 |
| ENSBTAG00000037735 | *GPR77* | 246.3381 | -1.2893 | 0.0000 | 0.0015 |
| ENSBTAG00000046788 |  | 6.0521 | -1.2848 | 0.0002 | 0.0099 |
| ENSBTAG00000034366 | *RGS2* | 259.1098 | -1.2777 | 0.0000 | 0.0000 |
| ENSBTAG00000001975 | *DRP2* | 34.7468 | -1.2749 | 0.0004 | 0.0157 |
| ENSBTAG00000000820 | *GNG11* | 2539.7779 | -1.2606 | 0.0000 | 0.0000 |
| ENSBTAG00000004272 | *ISG12(B)* | 31.9434 | -1.2465 | 0.0007 | 0.0212 |
| ENSBTAG00000024903 | *KCNJ16* | 19.9892 | -1.2367 | 0.0008 | 0.0227 |
| ENSBTAG00000006326 | *ALDH1L2* | 49.3254 | -1.2264 | 0.0004 | 0.0151 |
| ENSBTAG00000002135 | *CD69* | 23.0122 | -1.2224 | 0.0004 | 0.0136 |
| ENSBTAG00000039462 | *PAF* | 11.7232 | -1.2040 | 0.0007 | 0.0212 |
| ENSBTAG00000034449 | *GNG11* | 2402.9254 | -1.1942 | 0.0000 | 0.0003 |
| ENSBTAG00000014435 | *TCF19* | 20.6439 | -1.1927 | 0.0012 | 0.0306 |
| ENSBTAG00000005762 | *LYNX1* | 39.1081 | -1.1926 | 0.0003 | 0.0109 |
| ENSBTAG00000037649 | *VIPR2* | 1765.7668 | -1.1856 | 0.0000 | 0.0009 |
| ENSBTAG00000034391 | *EQTN* | 25.5356 | -1.1846 | 0.0002 | 0.0095 |
| ENSBTAG00000020087 | *CAMK2A* | 30.8585 | -1.1719 | 0.0006 | 0.0189 |
| ENSBTAG00000039813 | *GZMB* | 19.8258 | -1.1641 | 0.0007 | 0.0224 |
| ENSBTAG00000008121 | *RSPO3* | 158.8542 | -1.1638 | 0.0013 | 0.0326 |
| ENSBTAG00000007047 | *FZD10* | 353.8585 | -1.1599 | 0.0005 | 0.0178 |
| ENSBTAG00000044066 | *C1orf101* | 75.9297 | -1.1590 | 0.0000 | 0.0009 |
| ENSBTAG00000007235 | *NTNG2* | 16.2447 | -1.1532 | 0.0009 | 0.0247 |
| ENSBTAG00000045734 | *LRRC26* | 20.3894 | -1.1527 | 0.0014 | 0.0353 |
| ENSBTAG00000015089 | *LGALS1* | 1880.0646 | -1.1511 | 0.0000 | 0.0002 |
| ENSBTAG00000045647 | *ARL4C* | 68.4481 | -1.1483 | 0.0001 | 0.0041 |
| ENSBTAG00000007444 | *C1H21ORF7* | 62.6573 | -1.1478 | 0.0002 | 0.0072 |
| ENSBTAG00000011324 | *EMILIN1* | 2554.3086 | -1.1442 | 0.0000 | 0.0007 |
| ENSBTAG00000003369 | *SYT12* | 13.1700 | -1.1413 | 0.0019 | 0.0430 |
| ENSBTAG00000022120 | *FGB* | 62.1679 | -1.1331 | 0.0004 | 0.0136 |
| ENSBTAG00000022394 | *SAA1* | 542.0259 | -1.1292 | 0.0000 | 0.0000 |
| ENSBTAG00000001638 | *FGA* | 11.5653 | -1.1179 | 0.0011 | 0.0303 |
| ENSBTAG00000010732 | *MMP23B* | 412.2786 | -1.1066 | 0.0000 | 0.0000 |
| ENSBTAG00000006694 | *CXCL14* | 64.0321 | -1.1058 | 0.0020 | 0.0444 |
| ENSBTAG00000018833 | *SVOP* | 22.8872 | -1.1042 | 0.0020 | 0.0442 |
| ENSBTAG00000023207 | *FAM3D* | 675.7821 | -1.1026 | 0.0005 | 0.0171 |
| ENSBTAG00000016819 | *FABP3* | 40.9112 | -1.0949 | 0.0016 | 0.0380 |
| ENSBTAG00000001032 | *PYGM* | 85.9926 | -1.0937 | 0.0000 | 0.0005 |
| ENSBTAG00000021347 | *CWH43* | 89.6306 | -1.0855 | 0.0003 | 0.0114 |
| ENSBTAG00000009943 |  | 20.5326 | -1.0842 | 0.0011 | 0.0296 |
| ENSBTAG00000015539 | *RAPGEF5* | 99.1450 | -1.0841 | 0.0002 | 0.0072 |
| ENSBTAG00000017375 | *C6H4orf32* | 74.4060 | -1.0841 | 0.0000 | 0.0019 |
| ENSBTAG00000011338 | *NREP* | 197.0573 | -1.0836 | 0.0000 | 0.0025 |
| ENSBTAG00000009586 | *CDC25A* | 125.4069 | -1.0828 | 0.0000 | 0.0001 |
| ENSBTAG00000003502 | *SPOCK1* | 611.7476 | -1.0592 | 0.0002 | 0.0072 |
| ENSBTAG00000008541 | *MGST1* | 2811.7080 | -1.0579 | 0.0000 | 0.0000 |
| ENSBTAG00000024449 | *CENPF* | 53.2717 | -1.0578 | 0.0012 | 0.0306 |
| ENSBTAG00000019585 | *MYOM1* | 70.1907 | -1.0550 | 0.0006 | 0.0191 |
| ENSBTAG00000000432 | *TRAC* | 136.0538 | -1.0508 | 0.0009 | 0.0264 |
| ENSBTAG00000001604 |  | 86.9538 | -1.0412 | 0.0003 | 0.0115 |
| ENSBTAG00000016444 | *FAM134B* | 272.8660 | -1.0382 | 0.0000 | 0.0015 |
| ENSBTAG00000014319 | *DNM3* | 482.2982 | -1.0281 | 0.0000 | 0.0000 |
| ENSBTAG00000007626 | *IL2RG* | 120.0740 | -1.0217 | 0.0000 | 0.0001 |
| ENSBTAG00000018644 | *PDZRN3* | 187.2278 | -1.0211 | 0.0007 | 0.0212 |
| ENSBTAG00000001408 | *CNRIP1* | 232.9001 | -1.0203 | 0.0001 | 0.0055 |
| ENSBTAG00000016886 | *MT3* | 628.2400 | -1.0132 | 0.0002 | 0.0090 |
| ENSBTAG00000047227 |  | 51.6482 | -1.0092 | 0.0001 | 0.0062 |
| ENSBTAG00000001725 | *CXCL10* | 339.8474 | -0.9923 | 0.0002 | 0.0091 |
| ENSBTAG00000021969 | *KLHL29* | 20.9705 | -0.9914 | 0.0018 | 0.0416 |
| ENSBTAG00000000251 | *ALAD* | 1801.3423 | -0.9899 | 0.0000 | 0.0023 |
| ENSBTAG00000017869 | *CAV1* | 4170.4921 | -0.9897 | 0.0001 | 0.0062 |
| ENSBTAG00000016345 | *IL2RB* | 120.1334 | -0.9889 | 0.0000 | 0.0020 |
| ENSBTAG00000008401 | *PFKFB3* | 2144.3195 | -0.9874 | 0.0001 | 0.0057 |
| ENSBTAG00000021751 | *RASEF* | 67.7379 | -0.9857 | 0.0006 | 0.0182 |
| ENSBTAG00000020028 | *RBP1* | 84.9403 | -0.9832 | 0.0001 | 0.0047 |
| ENSBTAG00000014809 | *ANXA6* | 1144.2443 | -0.9805 | 0.0000 | 0.0023 |
| ENSBTAG00000030259 | *RASGRF2* | 158.3298 | -0.9706 | 0.0003 | 0.0108 |
| ENSBTAG00000001324 | *SLCO2A1* | 42.9072 | -0.9695 | 0.0007 | 0.0218 |
| ENSBTAG00000009086 | *LOXL1* | 499.7588 | -0.9657 | 0.0000 | 0.0002 |
| ENSBTAG00000013347 | *DMPK* | 765.4799 | -0.9604 | 0.0005 | 0.0163 |
| ENSBTAG00000003490 | *ELMO1* | 880.6951 | -0.9550 | 0.0015 | 0.0362 |
| ENSBTAG00000004413 | *RHOBTB3* | 1018.6914 | -0.9459 | 0.0007 | 0.0216 |
| ENSBTAG00000030426 |  | 38.9237 | -0.9445 | 0.0005 | 0.0170 |
| ENSBTAG00000004552 | *PBXIP1* | 3608.3734 | -0.9435 | 0.0002 | 0.0080 |
| ENSBTAG00000018043 | *LCAT* | 1040.8453 | -0.9421 | 0.0000 | 0.0000 |
| ENSBTAG00000014813 | *TCF23* | 584.5636 | -0.9334 | 0.0018 | 0.0417 |
| ENSBTAG00000009381 | *LCP2* | 177.4528 | -0.9311 | 0.0002 | 0.0076 |
| ENSBTAG00000012252 | *MOCOS* | 884.4818 | -0.9305 | 0.0000 | 0.0001 |
| ENSBTAG00000008583 | *KIAA1274* | 339.9911 | -0.9292 | 0.0005 | 0.0176 |
| ENSBTAG00000004777 | *S100B* | 21509.3280 | -0.9205 | 0.0014 | 0.0341 |
| ENSBTAG00000034147 | *OLFML2B* | 204.5489 | -0.9200 | 0.0023 | 0.0484 |
| ENSBTAG00000014529 | *GBP4* | 1046.7027 | -0.9196 | 0.0015 | 0.0366 |
| ENSBTAG00000026278 | *CLDN4* | 205.7491 | -0.9107 | 0.0019 | 0.0438 |
| ENSBTAG00000009769 |  | 126.2228 | -0.9041 | 0.0011 | 0.0288 |
| ENSBTAG00000037510 |  | 68.8582 | -0.9008 | 0.0000 | 0.0021 |
| ENSBTAG00000030940 | *GIMAP7* | 181.6554 | -0.8979 | 0.0000 | 0.0010 |
| ENSBTAG00000008817 | *LAMA4* | 964.2990 | -0.8904 | 0.0001 | 0.0051 |
| ENSBTAG00000020647 | *RASL11B* | 65.9350 | -0.8870 | 0.0003 | 0.0123 |
| ENSBTAG00000026236 | *AZGP1* | 15402.7763 | -0.8826 | 0.0017 | 0.0403 |
| ENSBTAG00000030434 | *FUCA1* | 2567.1988 | -0.8814 | 0.0001 | 0.0058 |
| ENSBTAG00000002041 | *SASH3* | 107.9946 | -0.8813 | 0.0000 | 0.0013 |
| ENSBTAG00000008966 | *TSPAN7* | 1969.6108 | -0.8794 | 0.0000 | 0.0015 |
| ENSBTAG00000017882 | *LRRC3* | 57.9635 | -0.8707 | 0.0004 | 0.0146 |
| ENSBTAG00000007192 | *SIDT2* | 1057.1982 | -0.8699 | 0.0000 | 0.0009 |
| ENSBTAG00000010178 | *PTPRD* | 433.5133 | -0.8662 | 0.0002 | 0.0083 |
| ENSBTAG00000021905 | *DGKB* | 1468.0291 | -0.8615 | 0.0011 | 0.0302 |
| ENSBTAG00000008103 | *ALDH1A1* | 4800.0198 | -0.8603 | 0.0000 | 0.0000 |
| ENSBTAG00000038810 | *SFTA2* | 345.7271 | -0.8592 | 0.0000 | 0.0021 |
| ENSBTAG00000019386 | *BOLA-NC* | 3246.1371 | -0.8584 | 0.0001 | 0.0064 |
| ENSBTAG00000027854 | *ARHGDIG* | 68.0201 | -0.8549 | 0.0011 | 0.0291 |
| ENSBTAG00000007206 | *STRIP2* | 221.5506 | -0.8522 | 0.0000 | 0.0007 |
| ENSBTAG00000000369 | *EPS8* | 1217.5681 | -0.8520 | 0.0000 | 0.0002 |
| ENSBTAG00000011359 | *CD7* | 61.7952 | -0.8508 | 0.0004 | 0.0154 |
| ENSBTAG00000005628 | *CD52* | 127.5284 | -0.8435 | 0.0011 | 0.0301 |
| ENSBTAG00000022580 | *FAM212B* | 399.9455 | -0.8411 | 0.0000 | 0.0002 |
| ENSBTAG00000013165 | *ENPP2* | 2287.6094 | -0.8391 | 0.0000 | 0.0000 |
| ENSBTAG00000014713 | *RARRES1* | 82930.4875 | -0.8389 | 0.0001 | 0.0051 |
| ENSBTAG00000009423 | *NIPAL1* | 164.7718 | -0.8333 | 0.0000 | 0.0002 |
| ENSBTAG00000005355 | *BAG2* | 568.6082 | -0.8262 | 0.0003 | 0.0115 |
| ENSBTAG00000007343 | *PDLIM7* | 1205.0354 | -0.8261 | 0.0001 | 0.0047 |
| ENSBTAG00000045567 | *PRR15* | 472.7602 | -0.8247 | 0.0006 | 0.0184 |
| ENSBTAG00000001060 | *CXCR4* | 353.7246 | -0.8240 | 0.0000 | 0.0000 |
| ENSBTAG00000000804 | *MOB3B* | 626.9239 | -0.8129 | 0.0004 | 0.0146 |
| ENSBTAG00000030921 | *FAM3B* | 751.4025 | -0.8099 | 0.0000 | 0.0023 |
| ENSBTAG00000019754 | *PRKCDBP* | 531.6239 | -0.8089 | 0.0009 | 0.0252 |
| ENSBTAG00000024822 | *SETX* | 3239.8350 | -0.8027 | 0.0016 | 0.0376 |
| ENSBTAG00000020810 | *CYB561* | 2781.4428 | -0.8000 | 0.0000 | 0.0011 |
| ENSBTAG00000008346 | *CRYBG3* | 381.2212 | -0.7983 | 0.0004 | 0.0143 |
| ENSBTAG00000003746 | *SCP2* | 6987.9038 | -0.7937 | 0.0010 | 0.0267 |
| ENSBTAG00000022396 | *SAA3* | 12323.5288 | -0.7932 | 0.0015 | 0.0362 |
| ENSBTAG00000007657 | *SCARA3* | 1136.2678 | -0.7902 | 0.0010 | 0.0273 |
| ENSBTAG00000020381 | *FCF1* | 50.0961 | -0.7813 | 0.0022 | 0.0479 |
| ENSBTAG00000012931 | *PLN* | 596.1477 | -0.7758 | 0.0017 | 0.0393 |
| ENSBTAG00000033248 | *CDH3* | 718.3115 | -0.7695 | 0.0018 | 0.0412 |
| ENSBTAG00000015710 | *CD3E* | 216.7786 | -0.7578 | 0.0006 | 0.0197 |
| ENSBTAG00000019072 | *PSD4* | 205.5752 | -0.7472 | 0.0001 | 0.0034 |
| ENSBTAG00000010597 | *GGCT* | 75.4949 | -0.7432 | 0.0006 | 0.0180 |
| ENSBTAG00000045604 | *TTC9* | 1027.9169 | -0.7421 | 0.0000 | 0.0000 |
| ENSBTAG00000018088 | *SETBP1* | 262.7739 | -0.7407 | 0.0000 | 0.0018 |
| ENSBTAG00000011864 | *RGMB* | 198.3795 | -0.7254 | 0.0015 | 0.0366 |
| ENSBTAG00000044017 | *MSRB3* | 163.8602 | -0.7199 | 0.0014 | 0.0359 |
| ENSBTAG00000018364 | *TMEM132A* | 2295.9843 | -0.7197 | 0.0001 | 0.0036 |
| ENSBTAG00000019497 | *ILDR2* | 461.4322 | -0.7180 | 0.0013 | 0.0338 |
| ENSBTAG00000018784 | *CTSZ* | 915.9701 | -0.7074 | 0.0000 | 0.0000 |
| ENSBTAG00000002669 | *RASSF4* | 1010.2605 | -0.7028 | 0.0001 | 0.0066 |
| ENSBTAG00000009087 | *GNG10* | 2504.6145 | -0.6971 | 0.0000 | 0.0000 |
| ENSBTAG00000002326 | *LGALS3* | 874.8374 | -0.6959 | 0.0000 | 0.0025 |
| ENSBTAG00000008098 | *DCLK2* | 379.8794 | -0.6934 | 0.0008 | 0.0235 |
| ENSBTAG00000016574 | *RGS11* | 299.3069 | -0.6923 | 0.0001 | 0.0037 |
| ENSBTAG00000017512 | *MAPT* | 206.3006 | -0.6811 | 0.0012 | 0.0311 |
| ENSBTAG00000004855 | *PRDX6* | 8870.0391 | -0.6784 | 0.0004 | 0.0149 |
| ENSBTAG00000001886 | *FAM122B* | 179.3594 | -0.6778 | 0.0005 | 0.0163 |
| ENSBTAG00000030669 | *FGGY* | 378.5179 | -0.6710 | 0.0005 | 0.0163 |
| ENSBTAG00000016818 | *ZCCHC17* | 570.1056 | -0.6691 | 0.0000 | 0.0023 |
| ENSBTAG00000031464 |  | 5576.3218 | -0.6680 | 0.0004 | 0.0151 |
| ENSBTAG00000001022 | *AMDHD2* | 672.7623 | -0.6665 | 0.0000 | 0.0004 |
| ENSBTAG00000006253 | *FLNC* | 4832.1614 | -0.6626 | 0.0000 | 0.0000 |
| ENSBTAG00000023744 | *YPEL5* | 723.3888 | -0.6622 | 0.0001 | 0.0068 |
| ENSBTAG00000017451 | *TSPAN17* | 365.1927 | -0.6537 | 0.0004 | 0.0157 |
| ENSBTAG00000009692 |  | 254.4263 | -0.6530 | 0.0000 | 0.0025 |
| ENSBTAG00000017135 | *CTSS* | 2478.6109 | -0.6419 | 0.0009 | 0.0260 |
| ENSBTAG00000004403 | *CD320* | 1392.6928 | -0.6381 | 0.0000 | 0.0011 |
| ENSBTAG00000005574 | *CLU* | 28986.0824 | -0.6378 | 0.0000 | 0.0000 |
| ENSBTAG00000008329 | *CYTIP* | 352.0946 | -0.6268 | 0.0001 | 0.0068 |
| ENSBTAG00000030369 | *IFFO1* | 86.6149 | -0.6249 | 0.0016 | 0.0380 |
| ENSBTAG00000013368 | *ANKRD22* | 163.7130 | -0.6201 | 0.0008 | 0.0239 |
| ENSBTAG00000014057 | *SLC35A1* | 885.9225 | -0.6200 | 0.0001 | 0.0051 |
| ENSBTAG00000007648 | *NET1* | 3264.2319 | -0.6172 | 0.0001 | 0.0059 |
| ENSBTAG00000047710 |  | 227.1913 | -0.6116 | 0.0007 | 0.0218 |
| ENSBTAG00000007596 | *GEM* | 1028.2433 | -0.6073 | 0.0005 | 0.0178 |
| ENSBTAG00000009979 | *HOXB2* | 1045.3526 | -0.6067 | 0.0000 | 0.0000 |
| ENSBTAG00000002846 | *TRAF3IP3* | 125.6638 | -0.6011 | 0.0007 | 0.0204 |
| ENSBTAG00000032051 |  | 1215.5483 | -0.5984 | 0.0000 | 0.0021 |
| ENSBTAG00000006364 | *C1orf85* | 431.7591 | -0.5900 | 0.0001 | 0.0033 |
| ENSBTAG00000006945 | *CCDC50* | 1253.7930 | -0.5896 | 0.0002 | 0.0079 |
| ENSBTAG00000006039 | *ARHGDIB* | 835.9558 | -0.5888 | 0.0006 | 0.0184 |
| ENSBTAG00000008293 | *GPCPD1* | 1289.9777 | -0.5870 | 0.0017 | 0.0397 |
| ENSBTAG00000013926 | *FCGRT* | 4701.2858 | -0.5869 | 0.0000 | 0.0010 |
| ENSBTAG00000032132 | *ELL3* | 250.7155 | -0.5863 | 0.0002 | 0.0098 |
| ENSBTAG00000019045 | *COMMD9* | 1072.0781 | -0.5853 | 0.0000 | 0.0013 |
| ENSBTAG00000013943 | *ZNF703* | 379.0033 | -0.5835 | 0.0000 | 0.0011 |
| ENSBTAG00000019354 | *PAX8* | 984.0392 | -0.5777 | 0.0006 | 0.0191 |
| ENSBTAG00000001805 | *TWSG1* | 2339.1802 | -0.5776 | 0.0000 | 0.0000 |
| ENSBTAG00000016037 | *BCKDHA* | 2143.1697 | -0.5754 | 0.0001 | 0.0069 |
| ENSBTAG00000000641 | *SKAP1* | 1330.7338 | -0.5733 | 0.0001 | 0.0044 |
| ENSBTAG00000005246 | *DIP2B* | 813.9515 | -0.5716 | 0.0002 | 0.0101 |
| ENSBTAG00000013759 | *TSPAN9* | 395.1481 | -0.5708 | 0.0004 | 0.0145 |
| ENSBTAG00000021002 | *ACP2* | 498.4018 | -0.5669 | 0.0016 | 0.0386 |
| ENSBTAG00000021216 | *HECW1* | 1167.9625 | -0.5664 | 0.0002 | 0.0092 |
| ENSBTAG00000002175 | *SRPK2* | 4113.7721 | -0.5659 | 0.0013 | 0.0326 |
| ENSBTAG00000009151 | *PYGO1* | 326.9238 | -0.5621 | 0.0001 | 0.0063 |
| ENSBTAG00000020042 | *KLHL28* | 296.1924 | -0.5613 | 0.0023 | 0.0499 |
| ENSBTAG00000030575 | *BHLHE41* | 1613.3077 | -0.5604 | 0.0000 | 0.0000 |
| ENSBTAG00000002847 | *D2HGDH* | 735.9526 | -0.5538 | 0.0004 | 0.0146 |
| ENSBTAG00000011693 | *LENG9* | 134.3744 | -0.5473 | 0.0017 | 0.0403 |
| ENSBTAG00000020119 | *RNF43* | 383.3696 | -0.5471 | 0.0021 | 0.0457 |
| ENSBTAG00000004438 | *XPNPEP1* | 1604.3459 | -0.5405 | 0.0021 | 0.0461 |
| ENSBTAG00000016625 | *KIAA0922* | 515.1226 | -0.5398 | 0.0000 | 0.0000 |
| ENSBTAG00000009157 | *SLC35B3* | 357.2622 | -0.5348 | 0.0002 | 0.0100 |
| ENSBTAG00000006324 | *NAB2* | 1053.8072 | -0.5334 | 0.0000 | 0.0002 |
| ENSBTAG00000011613 | *PLS3* | 1671.7677 | -0.5294 | 0.0004 | 0.0152 |
| ENSBTAG00000021105 | *IRAK4* | 579.1665 | -0.5293 | 0.0009 | 0.0250 |
| ENSBTAG00000011340 | *NSL1* | 192.2485 | -0.5282 | 0.0017 | 0.0402 |
| ENSBTAG00000001839 | *OCIAD2* | 1332.9155 | -0.5282 | 0.0000 | 0.0001 |
| ENSBTAG00000004457 | *ORAI1* | 241.6674 | -0.5248 | 0.0013 | 0.0325 |
| ENSBTAG00000000252 | *POLE3* | 1250.3214 | -0.5187 | 0.0000 | 0.0000 |
| ENSBTAG00000033221 | *CCDC152* | 4144.4105 | -0.5183 | 0.0006 | 0.0184 |
| ENSBTAG00000007977 | *LAMP5* | 4055.4018 | -0.5183 | 0.0010 | 0.0275 |
| ENSBTAG00000000224 | *TSPAN15* | 507.8377 | -0.5182 | 0.0001 | 0.0055 |
| ENSBTAG00000004895 | *AFAP1L2* | 2335.6619 | -0.5177 | 0.0000 | 0.0030 |
| ENSBTAG00000021942 | *HPS6* | 236.8257 | -0.5114 | 0.0007 | 0.0212 |
| ENSBTAG00000013922 | *MOSPD1* | 212.5919 | -0.5054 | 0.0018 | 0.0416 |
| ENSBTAG00000001497 | *MRAS* | 309.6882 | -0.5048 | 0.0010 | 0.0267 |
| ENSBTAG00000012818 | *PDLIM5* | 1271.2840 | -0.5026 | 0.0000 | 0.0000 |
| ENSBTAG00000013387 | *CHMP4B* | 1551.2484 | -0.5016 | 0.0019 | 0.0424 |
| ENSBTAG00000024663 | *LAD1* | 1426.8975 | -0.5006 | 0.0002 | 0.0103 |
| ENSBTAG00000004191 | *JMJD4* | 521.5461 | -0.5002 | 0.0017 | 0.0393 |
| ENSBTAG00000000394 | *BACE2* | 705.5012 | -0.4983 | 0.0021 | 0.0458 |
| ENSBTAG00000007755 | *APOBEC3Z3* | 318.7965 | -0.4967 | 0.0008 | 0.0224 |
| ENSBTAG00000017540 | *FAM13C* | 423.8843 | -0.4938 | 0.0006 | 0.0183 |
| ENSBTAG00000021879 | *VCL* | 3505.2314 | -0.4909 | 0.0011 | 0.0302 |
| ENSBTAG00000007953 | *FBXW7* | 276.7993 | -0.4885 | 0.0002 | 0.0087 |
| ENSBTAG00000002083 | *AUTS2* | 240.7632 | -0.4868 | 0.0002 | 0.0094 |
| ENSBTAG00000007836 | *PPA1* | 449.4518 | -0.4819 | 0.0004 | 0.0152 |
| ENSBTAG00000010191 | *PAK1* | 1509.8510 | -0.4736 | 0.0000 | 0.0001 |
| ENSBTAG00000046862 | *MARCKSL1* | 1886.6494 | -0.4726 | 0.0000 | 0.0022 |
| ENSBTAG00000019810 | *AARS* | 3139.6673 | -0.4725 | 0.0021 | 0.0457 |
| ENSBTAG00000020382 | *RIN2* | 954.7944 | -0.4714 | 0.0012 | 0.0311 |
| ENSBTAG00000011582 | *SERINC2* | 1485.1421 | -0.4664 | 0.0000 | 0.0005 |
| ENSBTAG00000037531 |  | 571.3274 | -0.4652 | 0.0005 | 0.0178 |
| ENSBTAG00000005729 | *FBXL4* | 380.3403 | -0.4650 | 0.0005 | 0.0175 |
| ENSBTAG00000007163 | *SLC44A1* | 1084.7073 | -0.4620 | 0.0015 | 0.0361 |
| ENSBTAG00000013423 | *GLRX5* | 874.5970 | -0.4577 | 0.0000 | 0.0001 |
| ENSBTAG00000018255 | *ACTN1* | 4042.7997 | -0.4541 | 0.0021 | 0.0461 |
| ENSBTAG00000014278 | *TBX2* | 1043.6632 | -0.4483 | 0.0020 | 0.0445 |
| ENSBTAG00000005934 | *TTYH3* | 294.1536 | -0.4483 | 0.0014 | 0.0345 |
| ENSBTAG00000008721 | *MANSC1* | 1142.1765 | -0.4482 | 0.0009 | 0.0252 |
| ENSBTAG00000021372 | *SEPT11* | 423.0279 | -0.4452 | 0.0000 | 0.0018 |
| ENSBTAG00000018449 | *NUMA1* | 7586.0120 | -0.4435 | 0.0002 | 0.0074 |
| ENSBTAG00000020648 | *SCFD2* | 596.2616 | -0.4433 | 0.0010 | 0.0272 |
| ENSBTAG00000014841 | *GBA* | 489.0073 | -0.4355 | 0.0017 | 0.0393 |
| ENSBTAG00000005364 | *HMBS* | 423.4026 | -0.4300 | 0.0007 | 0.0206 |
| ENSBTAG00000013869 | *SH3BP4* | 2201.6897 | -0.4155 | 0.0003 | 0.0127 |
| ENSBTAG00000039160 | *VAV1* | 321.6521 | -0.4152 | 0.0008 | 0.0235 |
| ENSBTAG00000026199 | *ACTB* | 21792.4068 | -0.4126 | 0.0005 | 0.0175 |
| ENSBTAG00000000105 | *RIT1* | 1780.6374 | -0.4071 | 0.0018 | 0.0412 |
| ENSBTAG00000004173 | *UBXN8* | 383.2600 | -0.3998 | 0.0005 | 0.0172 |
| ENSBTAG00000013851 | *BCL10* | 547.2686 | -0.3989 | 0.0005 | 0.0178 |
| ENSBTAG00000033367 | *DDO* | 729.2037 | -0.3984 | 0.0018 | 0.0412 |
| ENSBTAG00000000855 | *NXN* | 1495.3678 | -0.3980 | 0.0002 | 0.0101 |
| ENSBTAG00000007330 | *STXBP3* | 1409.6909 | -0.3929 | 0.0007 | 0.0222 |
| ENSBTAG00000003907 | *TSPAN14* | 2611.5917 | -0.3921 | 0.0007 | 0.0212 |
| ENSBTAG00000009830 | *PLEKHB1* | 2690.9340 | -0.3919 | 0.0010 | 0.0269 |
| ENSBTAG00000019163 | *CYB561D2* | 454.2013 | -0.3910 | 0.0003 | 0.0125 |
| ENSBTAG00000017584 | *PLCG1* | 1307.8717 | -0.3881 | 0.0005 | 0.0173 |
| ENSBTAG00000014374 | *CARD6* | 846.0342 | -0.3839 | 0.0003 | 0.0117 |
| ENSBTAG00000016199 | *CLINT1* | 3406.5107 | -0.3826 | 0.0023 | 0.0497 |
| ENSBTAG00000011798 | *STK38L* | 767.2699 | -0.3820 | 0.0002 | 0.0083 |
| ENSBTAG00000003192 | *TBC1D16* | 887.7116 | -0.3814 | 0.0002 | 0.0099 |
| ENSBTAG00000021427 | *HOXB3* | 878.4775 | -0.3805 | 0.0020 | 0.0447 |
| ENSBTAG00000010341 | *PWWP2A* | 448.5089 | -0.3765 | 0.0024 | 0.0499 |
| ENSBTAG00000018588 | *TMBIM6* | 23005.8484 | -0.3764 | 0.0000 | 0.0001 |
| ENSBTAG00000004915 | *PFN1* | 1863.4296 | -0.3750 | 0.0012 | 0.0307 |
| ENSBTAG00000002586 | *TCF12* | 2351.4666 | -0.3730 | 0.0003 | 0.0110 |
| ENSBTAG00000010717 | *SSBP3* | 1978.0411 | -0.3715 | 0.0005 | 0.0170 |
| ENSBTAG00000003579 | *FBXW4* | 483.2441 | -0.3706 | 0.0020 | 0.0442 |
| ENSBTAG00000035572 |  | 742.5735 | -0.3703 | 0.0000 | 0.0023 |
| ENSBTAG00000031435 | *SELT* | 2213.0571 | -0.3678 | 0.0009 | 0.0248 |
| ENSBTAG00000009475 | *PLXDC2* | 1302.0587 | -0.3658 | 0.0000 | 0.0008 |
| ENSBTAG00000008438 | *ARFIP1* | 1942.4265 | -0.3644 | 0.0018 | 0.0412 |
| ENSBTAG00000032055 | *VPS39* | 1139.8541 | -0.3623 | 0.0010 | 0.0273 |
| ENSBTAG00000022167 | *DERA* | 925.1630 | -0.3565 | 0.0010 | 0.0279 |
| ENSBTAG00000039591 | *TMEM167A* | 751.7001 | -0.3538 | 0.0013 | 0.0330 |
| ENSBTAG00000015363 | *CDC42SE1* | 1685.2978 | -0.3498 | 0.0002 | 0.0076 |
| ENSBTAG00000009761 | *ACTR2* | 3025.2809 | -0.3459 | 0.0012 | 0.0318 |
| ENSBTAG00000015155 | *PTPLAD1* | 1575.5405 | -0.3447 | 0.0006 | 0.0197 |
| ENSBTAG00000020645 | *GNAI2* | 4741.9862 | -0.3445 | 0.0000 | 0.0002 |
| ENSBTAG00000014700 | *DYNC1LI1* | 664.3845 | -0.3416 | 0.0002 | 0.0078 |
| ENSBTAG00000003276 | *PRKCH* | 954.2119 | -0.3415 | 0.0004 | 0.0146 |
| ENSBTAG00000011709 | *IMPA1* | 883.5939 | -0.3302 | 0.0002 | 0.0080 |
| ENSBTAG00000032436 | *UBC* | 15035.8431 | -0.3292 | 0.0003 | 0.0123 |
| ENSBTAG00000008977 | *TOMM6* | 506.8548 | -0.3134 | 0.0014 | 0.0341 |
| ENSBTAG00000016882 | *VPS26A* | 1929.6340 | -0.3104 | 0.0001 | 0.0043 |
| ENSBTAG00000009580 | *SH3BGRL3* | 3304.1442 | -0.3056 | 0.0001 | 0.0040 |
| ENSBTAG00000000646 | *ANAPC4* | 936.4344 | -0.3052 | 0.0009 | 0.0245 |
| ENSBTAG00000006985 | *TM7SF3* | 1533.6958 | -0.3048 | 0.0009 | 0.0260 |
| ENSBTAG00000008448 | *MEIS1* | 3185.3458 | -0.3046 | 0.0009 | 0.0252 |
| ENSBTAG00000004371 | *CS* | 795.3934 | -0.3035 | 0.0013 | 0.0326 |
| ENSBTAG00000007964 | *ARPC4* | 1308.8566 | -0.3018 | 0.0007 | 0.0212 |
| ENSBTAG00000004334 | *NCSTN* | 1463.0486 | -0.2999 | 0.0003 | 0.0108 |
| ENSBTAG00000001854 | *CIAPIN1* | 630.9220 | -0.2968 | 0.0019 | 0.0429 |
| ENSBTAG00000005627 | *PAFAH1B2* | 1672.0825 | -0.2912 | 0.0005 | 0.0178 |
| ENSBTAG00000025450 | *SYNE2* | 7786.0190 | -0.2898 | 0.0020 | 0.0442 |
| ENSBTAG00000003072 | *ACADVL* | 1898.8168 | -0.2892 | 0.0002 | 0.0099 |
| ENSBTAG00000014182 | *CTNNA1* | 11831.9494 | -0.2718 | 0.0004 | 0.0140 |
| ENSBTAG00000012497 | *KHDRBS1* | 2566.4634 | -0.2693 | 0.0000 | 0.0025 |
| ENSBTAG00000012244 | *TUBA1B* | 5487.0690 | -0.2692 | 0.0000 | 0.0004 |
| ENSBTAG00000003652 | *TMEM128* | 1193.1369 | -0.2686 | 0.0014 | 0.0351 |
| ENSBTAG00000004349 | *DAZAP2* | 6671.0437 | -0.2638 | 0.0001 | 0.0053 |
| ENSBTAG00000005757 | *FUS* | 4884.2716 | -0.2610 | 0.0022 | 0.0483 |
| ENSBTAG00000003935 | *RECS1* | 3659.1437 | -0.2510 | 0.0020 | 0.0442 |
| ENSBTAG00000040116 | *H1FX* | 2661.4734 | -0.2262 | 0.0003 | 0.0125 |
|  |  |  |  |  |  |
| Upregulated SF/SCL |  |  |  |  |  |
| ENSBTAG00000005299 | *EDNRB* | 1002.5413 | 1.3492 | 0.0000 | 0.0000 |
| ENSBTAG00000047764 |  | 49.1286 | 2.5188 | 0.0000 | 0.0000 |
| ENSBTAG00000004617 | *KIF19* | 3709.0874 | 1.6290 | 0.0000 | 0.0000 |
| ENSBTAG00000022028 | *DERL3* | 293.9064 | 1.3082 | 0.0000 | 0.0000 |
| ENSBTAG00000000818 | *PLEKHG7* | 452.2814 | 1.9880 | 0.0000 | 0.0000 |
| ENSBTAG00000033594 | *C12orf74* | 140.4612 | 2.1682 | 0.0000 | 0.0000 |
| ENSBTAG00000005734 | *GATA6* | 387.2165 | 1.6021 | 0.0000 | 0.0000 |
| ENSBTAG00000030258 | *CDC42EP1* | 2234.9737 | 1.5276 | 0.0000 | 0.0000 |
| ENSBTAG00000014830 | *PPP1R32* | 2775.4381 | 0.8025 | 0.0000 | 0.0000 |
| ENSBTAG00000004428 | *H2AFZ* | 1510.8515 | 0.6098 | 0.0000 | 0.0000 |
| ENSBTAG00000016519 | *MBOAT1* | 437.9822 | 1.0909 | 0.0000 | 0.0000 |
| ENSBTAG00000007553 | *KCNA5* | 235.3287 | 1.4584 | 0.0000 | 0.0000 |
| ENSBTAG00000004780 | *CDKL1* | 1256.3175 | 1.2553 | 0.0000 | 0.0000 |
| ENSBTAG00000002644 | *KCNQ4* | 101.4067 | 1.6564 | 0.0000 | 0.0000 |
| ENSBTAG00000016048 | *CPT1B* | 2772.0460 | 0.7200 | 0.0000 | 0.0000 |
| ENSBTAG00000015402 | *GREB1* | 1812.8741 | 1.1053 | 0.0000 | 0.0000 |
| ENSBTAG00000007071 | *RAI14* | 783.4505 | 1.0726 | 0.0000 | 0.0000 |
| ENSBTAG00000001093 | *KLHL23* | 330.3909 | 1.5027 | 0.0000 | 0.0000 |
| ENSBTAG00000005760 | *TBC1D4* | 474.7201 | 1.1408 | 0.0000 | 0.0000 |
| ENSBTAG00000039819 | *RPH3AL* | 244.7608 | 0.9152 | 0.0000 | 0.0000 |
| ENSBTAG00000013556 | *UNC13D* | 477.9077 | 1.5319 | 0.0000 | 0.0000 |
| ENSBTAG00000004594 | *MMP7* | 374.3747 | 1.6857 | 0.0000 | 0.0000 |
| ENSBTAG00000006052 | *PLCD3* | 862.3910 | 0.6715 | 0.0000 | 0.0000 |
| ENSBTAG00000005530 | *GNMT* | 285.3607 | 0.8421 | 0.0000 | 0.0000 |
| ENSBTAG00000011922 | *PLEC* | 5391.3746 | 0.3841 | 0.0000 | 0.0000 |
| ENSBTAG00000013403 | *AGBL5* | 1382.5519 | 0.5469 | 0.0000 | 0.0000 |
| ENSBTAG00000045966 | *GPRIN3* | 87.0497 | 1.9797 | 0.0000 | 0.0000 |
| ENSBTAG00000047113 |  | 180.9397 | 1.6948 | 0.0000 | 0.0000 |
| ENSBTAG00000015562 | *PLBD1* | 3408.9823 | 0.6453 | 0.0000 | 0.0000 |
| ENSBTAG00000010119 | *ALDH1A2* | 3599.5008 | 0.8463 | 0.0000 | 0.0000 |
| ENSBTAG00000005630 | *VWA7* | 1824.4095 | 0.7200 | 0.0000 | 0.0000 |
| ENSBTAG00000016244 | *VWA3A* | 2831.0939 | 0.5268 | 0.0000 | 0.0000 |
| ENSBTAG00000004635 | *LLGL1* | 2294.3365 | 0.5832 | 0.0000 | 0.0000 |
| ENSBTAG00000046266 | *TNFSF9* | 131.8527 | 1.5642 | 0.0000 | 0.0000 |
| ENSBTAG00000017722 | *F5* | 215.0887 | 1.9857 | 0.0000 | 0.0000 |
| ENSBTAG00000003829 | *HPN* | 173.9883 | 1.4199 | 0.0000 | 0.0000 |
| ENSBTAG00000018297 | *PLEKHH1* | 296.9155 | 0.9170 | 0.0000 | 0.0000 |
| ENSBTAG00000012120 | *TIPARP* | 2444.9402 | 0.9174 | 0.0000 | 0.0000 |
| ENSBTAG00000005745 | *HPSE* | 771.4936 | 1.4350 | 0.0000 | 0.0000 |
| ENSBTAG00000015106 | *DSP* | 21679.5418 | 0.4488 | 0.0000 | 0.0000 |
| ENSBTAG00000011713 |  | 937.0604 | 0.9512 | 0.0000 | 0.0000 |
| ENSBTAG00000008323 | *SNAP25* | 104.0391 | 1.6309 | 0.0000 | 0.0000 |
| ENSBTAG00000007496 | *P2RX2* | 3356.5453 | 0.7471 | 0.0000 | 0.0000 |
| ENSBTAG00000014885 | *MYOM3* | 758.0550 | 1.5052 | 0.0000 | 0.0000 |
| ENSBTAG00000018986 | *ACSL4* | 1010.1534 | 0.8397 | 0.0000 | 0.0000 |
| ENSBTAG00000047543 | *NTRK3* | 235.4368 | 1.4931 | 0.0000 | 0.0000 |
| ENSBTAG00000020887 | *CAV2.3* | 16.2268 | 1.9607 | 0.0000 | 0.0000 |
| ENSBTAG00000040298 | *GSTT1* | 2173.3830 | 0.3806 | 0.0000 | 0.0000 |
| ENSBTAG00000032544 | *SPAG1* | 1939.3245 | 0.5920 | 0.0000 | 0.0000 |
| ENSBTAG00000019806 | *YPEL2* | 198.7085 | 0.9276 | 0.0000 | 0.0000 |
| ENSBTAG00000021497 | *CDH23* | 302.9931 | 0.7482 | 0.0000 | 0.0000 |
| ENSBTAG00000008320 | *SEPT5* | 478.8025 | 0.6422 | 0.0000 | 0.0000 |
| ENSBTAG00000017839 | *TIAM1* | 717.1326 | 0.6486 | 0.0000 | 0.0000 |
| ENSBTAG00000020996 | *CLDND1* | 1962.8684 | 0.5962 | 0.0000 | 0.0000 |
| ENSBTAG00000004723 | *TEX2* | 2549.0077 | 0.5741 | 0.0000 | 0.0000 |
| ENSBTAG00000025441 | *HSPA1A* | 20268.6850 | 0.9394 | 0.0000 | 0.0000 |
| ENSBTAG00000016936 |  | 204.3163 | 0.8652 | 0.0000 | 0.0000 |
| ENSBTAG00000047167 |  | 287.5537 | 1.2745 | 0.0000 | 0.0000 |
| ENSBTAG00000025942 | *HPGD* | 172.1768 | 1.8237 | 0.0000 | 0.0000 |
| ENSBTAG00000003682 | *DNAJB13* | 1024.1147 | 0.6372 | 0.0000 | 0.0001 |
| ENSBTAG00000033565 |  | 2210.3884 | 0.7724 | 0.0000 | 0.0001 |
| ENSBTAG00000008250 | *SPRY4* | 1055.5355 | 0.5504 | 0.0000 | 0.0001 |
| ENSBTAG00000004597 | *PIM2* | 1267.6415 | 0.4543 | 0.0000 | 0.0001 |
| ENSBTAG00000000854 | *SEC16B* | 238.0390 | 1.0584 | 0.0000 | 0.0001 |
| ENSBTAG00000000088 | *ALCAM* | 468.1878 | 0.6906 | 0.0000 | 0.0001 |
| ENSBTAG00000006951 | *LMO2* | 2274.3660 | 0.5164 | 0.0000 | 0.0001 |
| ENSBTAG00000011586 | *HABP4* | 183.6383 | 0.9822 | 0.0000 | 0.0001 |
| ENSBTAG00000022991 | *NBEA* | 795.6087 | 0.5325 | 0.0000 | 0.0001 |
| ENSBTAG00000014391 | *CRB2* | 663.7366 | 0.8002 | 0.0000 | 0.0001 |
| ENSBTAG00000003455 | *ANKRD6* | 928.7230 | 0.8358 | 0.0000 | 0.0001 |
| ENSBTAG00000015097 | *MGC139698* | 1178.8812 | 1.0252 | 0.0000 | 0.0002 |
| ENSBTAG00000047410 | *CHDC2* | 554.0219 | 0.6069 | 0.0000 | 0.0002 |
| ENSBTAG00000016192 | *MDH1B* | 505.8925 | 0.5167 | 0.0000 | 0.0002 |
| ENSBTAG00000014409 | *MAP3K19* | 2641.3883 | 0.5873 | 0.0000 | 0.0002 |
| ENSBTAG00000019734 | *CD276* | 367.2271 | 0.6058 | 0.0000 | 0.0002 |
| ENSBTAG00000009125 | *ALDH1A3* | 123.0062 | 1.5549 | 0.0000 | 0.0003 |
| ENSBTAG00000001903 | *C11H2orf50* | 871.8147 | 0.5101 | 0.0000 | 0.0003 |
| ENSBTAG00000002721 | *ZCCHC11* | 1854.2176 | 0.6075 | 0.0000 | 0.0003 |
| ENSBTAG00000007933 | *C1H3orf58* | 943.3815 | 1.0418 | 0.0000 | 0.0003 |
| ENSBTAG00000004905 | *KRT19* | 4860.2979 | 0.7702 | 0.0000 | 0.0003 |
| ENSBTAG00000003601 | *ZMAT1* | 243.5060 | 0.7427 | 0.0000 | 0.0004 |
| ENSBTAG00000004330 | *ENO4* | 1278.6284 | 0.5807 | 0.0000 | 0.0004 |
| ENSBTAG00000000582 | *LY6G6E* | 745.8872 | 1.1081 | 0.0000 | 0.0004 |
| ENSBTAG00000007148 | *F2* | 1313.4759 | 0.7518 | 0.0000 | 0.0004 |
| ENSBTAG00000011330 |  | 162.3827 | 1.6090 | 0.0000 | 0.0004 |
| ENSBTAG00000030966 | *TAF6* | 1919.5172 | 0.6540 | 0.0000 | 0.0004 |
| ENSBTAG00000009471 | *CEP135* | 422.3201 | 1.1152 | 0.0000 | 0.0004 |
| ENSBTAG00000006396 | *GPI* | 2651.2502 | 0.4440 | 0.0000 | 0.0004 |
| ENSBTAG00000021636 | *GPR155* | 1724.9389 | 0.8466 | 0.0000 | 0.0005 |
| ENSBTAG00000008144 | *PPIL6* | 604.9161 | 0.5348 | 0.0000 | 0.0005 |
| ENSBTAG00000008313 | *KLF15* | 617.6796 | 0.7060 | 0.0000 | 0.0006 |
| ENSBTAG00000009239 | *SLC16A10* | 136.4094 | 0.8257 | 0.0000 | 0.0006 |
| ENSBTAG00000008176 | *KCNRG* | 879.4468 | 0.8144 | 0.0000 | 0.0006 |
| ENSBTAG00000014267 | *ZCCHC14* | 912.3495 | 0.4711 | 0.0000 | 0.0007 |
| ENSBTAG00000038823 |  | 124.8565 | 0.8225 | 0.0000 | 0.0008 |
| ENSBTAG00000019738 | *MDM1* | 607.1014 | 0.6408 | 0.0000 | 0.0008 |
| ENSBTAG00000000140 | *EPHX1* | 4504.5617 | 0.6731 | 0.0000 | 0.0008 |
| ENSBTAG00000013026 | *SNTN* | 576.0558 | 0.7961 | 0.0000 | 0.0008 |
| ENSBTAG00000015316 | *NPM1* | 735.5878 | 0.4168 | 0.0000 | 0.0009 |
| ENSBTAG00000001107 | *CACYBP* | 2260.4221 | 0.4059 | 0.0000 | 0.0009 |
| ENSBTAG00000009749 | *USP2* | 887.2208 | 0.5955 | 0.0000 | 0.0009 |
| ENSBTAG00000001279 | *MPP2* | 34.5576 | 1.3599 | 0.0000 | 0.0009 |
| ENSBTAG00000016615 | *ABHD6* | 679.0558 | 0.5091 | 0.0000 | 0.0010 |
| ENSBTAG00000010641 | *APH1B* | 41.5693 | 1.3689 | 0.0000 | 0.0010 |
| ENSBTAG00000011322 | *HIPK1* | 4902.3071 | 0.4107 | 0.0000 | 0.0010 |
| ENSBTAG00000004921 |  | 1052.7282 | 0.8564 | 0.0000 | 0.0011 |
| ENSBTAG00000020073 | *NCEH1* | 397.4106 | 0.6950 | 0.0000 | 0.0011 |
| ENSBTAG00000021284 | *RSPH1* | 2539.1019 | 0.6770 | 0.0000 | 0.0011 |
| ENSBTAG00000009161 | *TIGD4* | 223.3128 | 1.0179 | 0.0000 | 0.0012 |
| ENSBTAG00000004869 | *CXADR* | 522.9702 | 0.4799 | 0.0000 | 0.0012 |
| ENSBTAG00000017310 | *EVA1C* | 591.9484 | 0.7079 | 0.0000 | 0.0014 |
| ENSBTAG00000006528 | *BBS9* | 958.0559 | 0.5368 | 0.0000 | 0.0014 |
| ENSBTAG00000020335 | *RNF32* | 381.1082 | 0.7558 | 0.0000 | 0.0014 |
| ENSBTAG00000014259 | *KCTD1* | 1231.0594 | 0.4027 | 0.0000 | 0.0016 |
| ENSBTAG00000012158 | *C16H1orf158* | 829.6030 | 0.5170 | 0.0000 | 0.0016 |
| ENSBTAG00000020929 | *ARMC3* | 3300.6477 | 0.7276 | 0.0000 | 0.0017 |
| ENSBTAG00000012693 |  | 69.7860 | 1.0740 | 0.0000 | 0.0018 |
| ENSBTAG00000003120 | *MGC139239* | 31.0458 | 1.5327 | 0.0000 | 0.0018 |
| ENSBTAG00000035776 | *C4orf22* | 428.1495 | 0.8817 | 0.0000 | 0.0020 |
| ENSBTAG00000032509 | *FAM166B* | 2441.5569 | 0.4547 | 0.0000 | 0.0021 |
| ENSBTAG00000002972 | *CASC1* | 2151.9164 | 0.5361 | 0.0000 | 0.0021 |
| ENSBTAG00000003059 | *C1H21orf59* | 4130.6992 | 0.3208 | 0.0000 | 0.0022 |
| ENSBTAG00000018989 | *GRM3* | 110.2320 | 1.4928 | 0.0000 | 0.0022 |
| ENSBTAG00000048246 |  | 83.0930 | 0.9273 | 0.0000 | 0.0023 |
| ENSBTAG00000020379 | *AREL1* | 929.6589 | 0.3641 | 0.0000 | 0.0023 |
| ENSBTAG00000001675 | *ITPKA* | 489.3019 | 1.0764 | 0.0000 | 0.0024 |
| ENSBTAG00000022382 | *TMEM218* | 959.4604 | 0.4366 | 0.0000 | 0.0024 |
| ENSBTAG00000012558 | *ADAMTS12* | 209.5866 | 1.1313 | 0.0000 | 0.0025 |
| ENSBTAG00000023765 | *C16orf46* | 276.2388 | 0.5703 | 0.0000 | 0.0027 |
| ENSBTAG00000006021 | *CEP250* | 827.7596 | 0.5559 | 0.0000 | 0.0029 |
| ENSBTAG00000048173 | *SMIM10* | 70.5977 | 0.9514 | 0.0000 | 0.0030 |
| ENSBTAG00000014135 | *WDR17* | 751.5206 | 0.6124 | 0.0000 | 0.0031 |
| ENSBTAG00000007223 | *FBXO2* | 856.7688 | 0.7762 | 0.0000 | 0.0031 |
| ENSBTAG00000019164 | *RHOBTB1* | 802.8906 | 0.7391 | 0.0001 | 0.0032 |
| ENSBTAG00000011268 | *TMEM64* | 116.9288 | 0.8155 | 0.0001 | 0.0034 |
| ENSBTAG00000005908 |  | 2134.0963 | 0.4301 | 0.0001 | 0.0035 |
| ENSBTAG00000040361 | *LFNG* | 1453.4556 | 0.5167 | 0.0001 | 0.0037 |
| ENSBTAG00000020696 | *HECW2* | 122.2592 | 1.4307 | 0.0001 | 0.0037 |
| ENSBTAG00000030587 | *LASP1* | 7082.3197 | 0.3250 | 0.0001 | 0.0037 |
| ENSBTAG00000032862 | *DYDC2* | 1832.9110 | 0.5585 | 0.0001 | 0.0038 |
| ENSBTAG00000013320 | *TSPAN1* | 4125.8085 | 0.5015 | 0.0001 | 0.0040 |
| ENSBTAG00000020048 | *MAPK10* | 597.0986 | 0.4623 | 0.0001 | 0.0040 |
| ENSBTAG00000010008 | *TTC12* | 2077.8706 | 0.6344 | 0.0001 | 0.0041 |
| ENSBTAG00000026637 |  | 562.5640 | 0.7846 | 0.0001 | 0.0043 |
| ENSBTAG00000005012 | *HSPH1* | 7213.7420 | 0.6032 | 0.0001 | 0.0043 |
| ENSBTAG00000017661 | *RFX2* | 1777.0663 | 0.4843 | 0.0001 | 0.0043 |
| ENSBTAG00000009208 | *TCTN1* | 2001.0446 | 0.5370 | 0.0001 | 0.0045 |
| ENSBTAG00000014512 | *WDR19* | 2820.8565 | 0.4508 | 0.0001 | 0.0046 |
| ENSBTAG00000010841 | *FMO5* | 2042.4217 | 0.4216 | 0.0001 | 0.0050 |
| ENSBTAG00000000782 | *KDR* | 2736.6361 | 0.7048 | 0.0001 | 0.0051 |
| ENSBTAG00000003371 | *JAMP* | 993.6157 | 0.3754 | 0.0001 | 0.0051 |
| ENSBTAG00000021365 | *CDHR3* | 2088.2595 | 0.9297 | 0.0001 | 0.0051 |
| ENSBTAG00000005787 | *IFT57* | 5197.2293 | 0.3283 | 0.0001 | 0.0051 |
| ENSBTAG00000005675 | *SLC44A4* | 2583.0183 | 0.7618 | 0.0001 | 0.0051 |
| ENSBTAG00000001754 | *AHCYL2* | 597.8913 | 0.6019 | 0.0001 | 0.0052 |
| ENSBTAG00000034680 | *FAM131C* | 59.8110 | 1.0297 | 0.0001 | 0.0053 |
| ENSBTAG00000010978 | *PROX1* | 73.5040 | 0.9319 | 0.0001 | 0.0055 |
| ENSBTAG00000007271 | *TCTN2* | 1052.1030 | 0.5263 | 0.0001 | 0.0055 |
| ENSBTAG00000014689 | *CSPP1* | 1857.2252 | 0.4583 | 0.0001 | 0.0055 |
| ENSBTAG00000019062 | *FCHSD2* | 1290.1171 | 0.3840 | 0.0001 | 0.0058 |
| ENSBTAG00000027642 | *C11orf42* | 66.3938 | 0.9027 | 0.0001 | 0.0059 |
| ENSBTAG00000018908 | *GFOD2* | 566.0939 | 0.4630 | 0.0001 | 0.0060 |
| ENSBTAG00000021287 | *SLC16A7* | 115.3460 | 0.7725 | 0.0001 | 0.0060 |
| ENSBTAG00000046173 | *ALG12* | 724.5950 | 0.4593 | 0.0001 | 0.0060 |
| ENSBTAG00000019002 | *SLC2A12* | 1234.9000 | 0.3889 | 0.0001 | 0.0060 |
| ENSBTAG00000015825 | *CCDC65* | 4182.5475 | 0.4768 | 0.0001 | 0.0060 |
| ENSBTAG00000030667 | *CCDC114* | 1451.5928 | 0.4809 | 0.0001 | 0.0062 |
| ENSBTAG00000000792 | *AJAP1* | 14.0778 | 1.3982 | 0.0001 | 0.0062 |
| ENSBTAG00000007650 | *SLC38A11* | 32.7044 | 1.3566 | 0.0001 | 0.0066 |
| ENSBTAG00000025522 | *UNC13C* | 19.6737 | 1.4036 | 0.0001 | 0.0066 |
| ENSBTAG00000002615 | *LONRF3* | 1426.4839 | 0.9953 | 0.0001 | 0.0068 |
| ENSBTAG00000018638 | *CC2D2A* | 1455.1529 | 0.5714 | 0.0001 | 0.0068 |
| ENSBTAG00000031669 | *CTNNA2* | 303.9450 | 1.0890 | 0.0001 | 0.0070 |
| ENSBTAG00000031631 | *FAM132A* | 55.6828 | 1.0074 | 0.0001 | 0.0071 |
| ENSBTAG00000018062 | *KIAA1522* | 1169.4240 | 0.3993 | 0.0002 | 0.0072 |
| ENSBTAG00000000943 | *ZNF286A* | 944.9165 | 0.6042 | 0.0002 | 0.0073 |
| ENSBTAG00000003172 | *MEIS2* | 2517.5716 | 0.4855 | 0.0002 | 0.0073 |
| ENSBTAG00000000057 | *THBS3* | 497.1488 | 0.8979 | 0.0002 | 0.0074 |
| ENSBTAG00000014430 | *CDC26* | 371.0470 | 0.6388 | 0.0002 | 0.0074 |
| ENSBTAG00000047461 |  | 1166.5936 | 0.5433 | 0.0002 | 0.0077 |
| ENSBTAG00000004168 |  | 466.6729 | 0.7594 | 0.0002 | 0.0077 |
| ENSBTAG00000016147 | *CETN4* | 3721.1540 | 0.5281 | 0.0002 | 0.0079 |
| ENSBTAG00000015280 | *KIF2C* | 145.2450 | 1.3097 | 0.0002 | 0.0080 |
| ENSBTAG00000015792 |  | 2586.6981 | 0.6605 | 0.0002 | 0.0085 |
| ENSBTAG00000015942 | *DNAJA4* | 2877.1349 | 0.2580 | 0.0002 | 0.0087 |
| ENSBTAG00000007483 | *UNC119B* | 820.0872 | 0.3359 | 0.0002 | 0.0087 |
| ENSBTAG00000010963 | *IL16* | 413.6131 | 0.5759 | 0.0002 | 0.0090 |
| ENSBTAG00000046017 | *POPDC3* | 1293.9909 | 0.6911 | 0.0002 | 0.0095 |
| ENSBTAG00000007186 | *ARHGAP39* | 1293.6724 | 0.4008 | 0.0002 | 0.0095 |
| ENSBTAG00000006234 | *NPR1* | 1202.8561 | 1.0168 | 0.0002 | 0.0095 |
| ENSBTAG00000048263 | *LRRC56* | 590.5806 | 0.5152 | 0.0002 | 0.0099 |
| ENSBTAG00000021183 |  | 591.1854 | 0.4426 | 0.0002 | 0.0101 |
| ENSBTAG00000012434 | *ENOX1* | 322.3096 | 0.7269 | 0.0002 | 0.0101 |
| ENSBTAG00000006132 | *DENND3* | 463.7083 | 0.5665 | 0.0002 | 0.0102 |
| ENSBTAG00000009638 | *PRMT7* | 726.5123 | 0.5096 | 0.0003 | 0.0105 |
| ENSBTAG00000004561 | *PAX6* | 13.3465 | 1.3406 | 0.0003 | 0.0109 |
| ENSBTAG00000018157 | *IFT172* | 3422.7932 | 0.4934 | 0.0003 | 0.0110 |
| ENSBTAG00000012135 | *LARP1B* | 548.5944 | 0.5854 | 0.0003 | 0.0110 |
| ENSBTAG00000000770 | *PGM2L1* | 259.8800 | 1.1582 | 0.0003 | 0.0110 |
| ENSBTAG00000001105 | *ANXA4* | 6070.1914 | 0.7335 | 0.0003 | 0.0110 |
| ENSBTAG00000013420 | *FSD1L* | 439.4606 | 0.6543 | 0.0003 | 0.0110 |
| ENSBTAG00000013042 | *EXOC5* | 1904.0287 | 0.2600 | 0.0003 | 0.0112 |
| ENSBTAG00000018487 | *CCDC19* | 1885.4383 | 0.3777 | 0.0003 | 0.0113 |
| ENSBTAG00000002522 | *SYT5* | 3000.3711 | 0.4933 | 0.0003 | 0.0119 |
| ENSBTAG00000003089 | *RHPN2* | 1617.1790 | 0.5149 | 0.0003 | 0.0120 |
| ENSBTAG00000009812 | *CXCL6* | 83.8137 | 1.3084 | 0.0003 | 0.0123 |
| ENSBTAG00000014982 | *C7H19orf71* | 124.0419 | 0.7625 | 0.0003 | 0.0123 |
| ENSBTAG00000040380 |  | 1008.2821 | 0.3257 | 0.0003 | 0.0130 |
| ENSBTAG00000046160 | *SMIM13* | 1083.5340 | 0.9852 | 0.0003 | 0.0131 |
| ENSBTAG00000001470 | *DTX2* | 570.4924 | 0.3831 | 0.0004 | 0.0136 |
| ENSBTAG00000047336 |  | 10.4842 | 1.3010 | 0.0004 | 0.0140 |
| ENSBTAG00000004876 | *RABL2B* | 5490.7420 | 0.2412 | 0.0004 | 0.0140 |
| ENSBTAG00000025535 | *GLYATL3* | 10.1783 | 1.2831 | 0.0004 | 0.0140 |
| ENSBTAG00000009833 | *DTHD1* | 595.5457 | 0.4470 | 0.0004 | 0.0142 |
| ENSBTAG00000032034 | *DYNLL1* | 1933.6320 | 0.2413 | 0.0004 | 0.0144 |
| ENSBTAG00000007415 | *SLC7A8* | 209.2404 | 0.8943 | 0.0004 | 0.0144 |
| ENSBTAG00000015321 | *TRERF1* | 264.7501 | 0.7285 | 0.0004 | 0.0144 |
| ENSBTAG00000027321 | *CCDC18* | 131.3765 | 0.7696 | 0.0004 | 0.0146 |
| ENSBTAG00000020070 | *ABCC3* | 1630.6924 | 0.4724 | 0.0004 | 0.0149 |
| ENSBTAG00000019107 | *GAS7* | 475.8920 | 0.3988 | 0.0004 | 0.0151 |
| ENSBTAG00000014354 | *FXYD6* | 3938.3223 | 0.4890 | 0.0004 | 0.0151 |
| ENSBTAG00000012718 | *XK* | 516.6085 | 0.5998 | 0.0004 | 0.0151 |
| ENSBTAG00000008464 | *ABCB9* | 63.0541 | 0.9689 | 0.0004 | 0.0151 |
| ENSBTAG00000021026 |  | 176.3484 | 0.5852 | 0.0004 | 0.0152 |
| ENSBTAG00000018682 | *SETD4* | 556.9418 | 0.4390 | 0.0004 | 0.0157 |
| ENSBTAG00000011397 | *UNC13B* | 897.0876 | 0.4958 | 0.0004 | 0.0157 |
| ENSBTAG00000007256 | *DYRK2* | 920.5165 | 0.5209 | 0.0005 | 0.0165 |
| ENSBTAG00000007962 | *ATP9A* | 2003.6782 | 0.3945 | 0.0005 | 0.0165 |
| ENSBTAG00000020839 | *MEGF6* | 52.7718 | 1.2844 | 0.0005 | 0.0165 |
| ENSBTAG00000017010 | *C1H21ORF70* | 303.4984 | 0.5274 | 0.0005 | 0.0167 |
| ENSBTAG00000012194 | *DENND2C* | 146.2137 | 0.8164 | 0.0005 | 0.0167 |
| ENSBTAG00000047416 | *HEPH* | 470.8949 | 0.5791 | 0.0005 | 0.0167 |
| ENSBTAG00000003322 | *TTLL7* | 186.8465 | 0.6656 | 0.0005 | 0.0170 |
| ENSBTAG00000018869 | *IGSF6* | 112.0629 | 1.0854 | 0.0005 | 0.0172 |
| ENSBTAG00000002020 | *CREBRF* | 881.4903 | 0.4226 | 0.0005 | 0.0173 |
| ENSBTAG00000008028 | *CHN1* | 1009.2720 | 0.4766 | 0.0005 | 0.0173 |
| ENSBTAG00000019028 | *STOX1* | 501.5480 | 0.7014 | 0.0005 | 0.0173 |
| ENSBTAG00000001156 | *ST3GAL1* | 519.3117 | 0.6344 | 0.0005 | 0.0175 |
| ENSBTAG00000009994 | *EML5* | 281.9428 | 0.7374 | 0.0005 | 0.0175 |
| ENSBTAG00000012040 | *GIT1* | 1473.6293 | 0.3131 | 0.0005 | 0.0178 |
| ENSBTAG00000045937 |  | 1766.9746 | 0.3615 | 0.0005 | 0.0178 |
| ENSBTAG00000032031 | *ZBTB12* | 343.2615 | 0.6614 | 0.0005 | 0.0178 |
| ENSBTAG00000009314 | *C1orf168* | 1305.9352 | 0.6823 | 0.0005 | 0.0178 |
| ENSBTAG00000011017 | *ABI2* | 416.4456 | 0.3903 | 0.0006 | 0.0179 |
| ENSBTAG00000001331 | *FAM194A* | 503.0589 | 0.5544 | 0.0006 | 0.0183 |
| ENSBTAG00000020264 | *CCDC181* | 474.7831 | 0.5116 | 0.0006 | 0.0187 |
| ENSBTAG00000033386 | *C12H13ORF26* | 398.4372 | 0.6082 | 0.0006 | 0.0189 |
| ENSBTAG00000012708 | *SERPINI1* | 1274.2710 | 0.6137 | 0.0006 | 0.0190 |
| ENSBTAG00000001124 | *GALC* | 3245.5302 | 0.6326 | 0.0006 | 0.0191 |
| ENSBTAG00000011731 | *PNMT* | 36.2971 | 1.0232 | 0.0006 | 0.0197 |
| ENSBTAG00000008288 | *ANKRD26* | 1170.4166 | 0.5513 | 0.0006 | 0.0200 |
| ENSBTAG00000006280 | *RBFOX3* | 238.4440 | 1.0168 | 0.0006 | 0.0201 |
| ENSBTAG00000014038 | *CDKL2* | 300.2383 | 0.5154 | 0.0006 | 0.0202 |
| ENSBTAG00000023384 |  | 244.8607 | 0.5057 | 0.0007 | 0.0204 |
| ENSBTAG00000017644 | *KIF17* | 693.1174 | 0.5648 | 0.0007 | 0.0204 |
| ENSBTAG00000018465 | *KCNAB1* | 47.1775 | 1.2053 | 0.0007 | 0.0204 |
| ENSBTAG00000020991 | *COL19A1* | 34.4875 | 1.2093 | 0.0007 | 0.0207 |
| ENSBTAG00000005359 | *TGFB2* | 292.1281 | 0.8302 | 0.0007 | 0.0212 |
| ENSBTAG00000015801 | *EFNB1* | 869.2725 | 0.8565 | 0.0007 | 0.0214 |
| ENSBTAG00000009140 | *CXorf22* | 749.7565 | 0.5845 | 0.0007 | 0.0214 |
| ENSBTAG00000002348 | *SLC4A4* | 76.3719 | 0.7553 | 0.0008 | 0.0228 |
| ENSBTAG00000006676 | *FIBIN* | 164.4235 | 1.0842 | 0.0008 | 0.0229 |
| ENSBTAG00000020573 | *SCUBE2* | 439.5306 | 1.0741 | 0.0008 | 0.0229 |
| ENSBTAG00000018106 | *SPATA18* | 2123.6169 | 0.4709 | 0.0008 | 0.0233 |
| ENSBTAG00000004193 | *VEZF1* | 3237.6561 | 0.3452 | 0.0008 | 0.0235 |
| ENSBTAG00000014137 | *CEP164* | 535.5651 | 0.4047 | 0.0008 | 0.0235 |
| ENSBTAG00000013505 | *IQCA1* | 1792.7415 | 0.4959 | 0.0008 | 0.0235 |
| ENSBTAG00000002594 | *ZNF436* | 1015.2923 | 0.5139 | 0.0008 | 0.0235 |
| ENSBTAG00000012609 | *SCO2* | 1765.9814 | 0.8385 | 0.0008 | 0.0240 |
| ENSBTAG00000020700 | *CLCN6* | 186.3196 | 0.6810 | 0.0008 | 0.0243 |
| ENSBTAG00000016515 | *EFNA5* | 98.6671 | 0.9465 | 0.0008 | 0.0245 |
| ENSBTAG00000007566 | *CEP170* | 547.3655 | 0.3557 | 0.0009 | 0.0245 |
| ENSBTAG00000047587 |  | 339.1117 | 0.9255 | 0.0009 | 0.0245 |
| ENSBTAG00000018509 | *ETNK2* | 113.5602 | 0.6967 | 0.0009 | 0.0246 |
| ENSBTAG00000047254 | *ABCA2* | 2987.8946 | 0.7349 | 0.0009 | 0.0247 |
| ENSBTAG00000022000 | *CACNB2* | 253.5671 | 0.8996 | 0.0009 | 0.0247 |
| ENSBTAG00000010878 |  | 6953.5082 | 0.3854 | 0.0009 | 0.0251 |
| ENSBTAG00000011059 | *CDKN2C* | 436.4977 | 0.5917 | 0.0009 | 0.0252 |
| ENSBTAG00000033708 |  | 10.5390 | 1.2200 | 0.0009 | 0.0252 |
| ENSBTAG00000019964 | *GAS6* | 4097.9221 | 0.3801 | 0.0009 | 0.0253 |
| ENSBTAG00000024476 | *CKS1B* | 135.1306 | 0.6100 | 0.0009 | 0.0258 |
| ENSBTAG00000008409 | *MYC* | 837.7016 | 0.9584 | 0.0009 | 0.0262 |
| ENSBTAG00000031682 | *ZNF484* | 446.4758 | 0.3815 | 0.0010 | 0.0267 |
| ENSBTAG00000012854 | *GSDMB* | 298.6461 | 0.8426 | 0.0010 | 0.0275 |
| ENSBTAG00000011569 | *AADAC* | 147.8056 | 1.0139 | 0.0010 | 0.0275 |
| ENSBTAG00000008280 | *HNF4G* | 119.1320 | 0.6599 | 0.0011 | 0.0287 |
| ENSBTAG00000011822 | *C1orf228* | 357.3051 | 0.6868 | 0.0011 | 0.0295 |
| ENSBTAG00000005937 | *TTLL9* | 1207.9892 | 0.3959 | 0.0011 | 0.0301 |
| ENSBTAG00000000875 | *AZI1* | 565.9811 | 0.4475 | 0.0011 | 0.0301 |
| ENSBTAG00000005812 | *TP73* | 989.2334 | 0.6831 | 0.0011 | 0.0301 |
| ENSBTAG00000021304 | *TP53BP1* | 3071.6495 | 0.1966 | 0.0012 | 0.0306 |
| ENSBTAG00000019726 | *ANKEF1* | 35.8240 | 0.9364 | 0.0012 | 0.0306 |
| ENSBTAG00000019807 | *COL27A1* | 645.6168 | 1.0073 | 0.0012 | 0.0306 |
| ENSBTAG00000018903 | *ACD* | 439.6531 | 0.4710 | 0.0012 | 0.0306 |
| ENSBTAG00000016275 | *AMDHD1* | 51.4141 | 0.8856 | 0.0012 | 0.0307 |
| ENSBTAG00000006752 | *PFKFB4* | 111.7791 | 0.9578 | 0.0012 | 0.0307 |
| ENSBTAG00000010617 | *KRTCAP2* | 962.0026 | 0.2924 | 0.0012 | 0.0308 |
| ENSBTAG00000012182 | *DIRAS3* | 13.9223 | 1.1906 | 0.0012 | 0.0311 |
| ENSBTAG00000034586 |  | 880.2843 | 0.8821 | 0.0012 | 0.0318 |
| ENSBTAG00000001360 | *RPS12* | 2780.8692 | 0.3069 | 0.0012 | 0.0322 |
| ENSBTAG00000013509 | *TTC16* | 343.3503 | 0.5287 | 0.0012 | 0.0322 |
| ENSBTAG000000233380 | *bta-mir-2900* | 582.3346 | 0.5122 | 0.0013 | 0.0324 |
| ENSBTAG00000030686 | *RAVER2* | 120.1495 | 0.7693 | 0.0013 | 0.0327 |
| ENSBTAG00000020616 | *DGKH* | 848.4816 | 0.3186 | 0.0013 | 0.0334 |
| ENSBTAG00000030166 | *BTBD17* | 13.2042 | 1.1819 | 0.0013 | 0.0334 |
| ENSBTAG00000023776 | *C9orf116* | 1936.9042 | 0.3650 | 0.0013 | 0.0340 |
| ENSBTAG00000001104 | *CMTM4* | 512.2059 | 0.5702 | 0.0013 | 0.0340 |
| ENSBTAG00000013836 |  | 134.4951 | 0.7676 | 0.0014 | 0.0341 |
| ENSBTAG00000010820 | *WNT11* | 66.6152 | 1.1060 | 0.0014 | 0.0344 |
| ENSBTAG00000021984 | *C21orf58* | 1257.8935 | 0.5216 | 0.0014 | 0.0353 |
| ENSBTAG00000025494 | *PAQR8* | 418.1022 | 0.8010 | 0.0014 | 0.0353 |
| ENSBTAG00000033217 | *TPM3* | 5205.4846 | 0.4425 | 0.0015 | 0.0361 |
| ENSBTAG00000046333 |  | 65.6315 | 0.9599 | 0.0015 | 0.0361 |
| ENSBTAG00000013806 | *EFHB* | 661.9608 | 0.5952 | 0.0015 | 0.0362 |
| ENSBTAG00000008557 |  | 10716.7260 | 0.1466 | 0.0015 | 0.0362 |
| ENSBTAG00000016170 | *KCNJ11* | 294.3764 | 0.7372 | 0.0015 | 0.0364 |
| ENSBTAG00000015285 | *RPS8* | 32107.8008 | 0.1979 | 0.0015 | 0.0364 |
| ENSBTAG00000001188 | *ROM1* | 792.5963 | 0.5813 | 0.0015 | 0.0364 |
| ENSBTAG00000021601 | *PARD6B* | 360.0487 | 0.4375 | 0.0015 | 0.0367 |
| ENSBTAG00000020040 | *LPCAT4* | 5714.3588 | 0.4153 | 0.0015 | 0.0367 |
| ENSBTAG00000039035 | *HSPA6* | 137.3376 | 1.0482 | 0.0015 | 0.0372 |
| ENSBTAG00000000271 | *MNS1* | 1515.4230 | 0.4374 | 0.0016 | 0.0380 |
| ENSBTAG00000008576 | *PLEKHD1* | 1015.3417 | 0.9184 | 0.0016 | 0.0380 |
| ENSBTAG00000008597 | *SLC45A4* | 350.2214 | 0.6613 | 0.0016 | 0.0384 |
| ENSBTAG00000002317 | *PTN* | 1793.8361 | 0.7749 | 0.0016 | 0.0384 |
| ENSBTAG00000030453 | *ZC3H14* | 777.7153 | 0.4251 | 0.0016 | 0.0385 |
| ENSBTAG00000013638 | *IQCH* | 577.3766 | 0.5694 | 0.0016 | 0.0386 |
| ENSBTAG00000048156 |  | 50.3570 | 1.1241 | 0.0016 | 0.0387 |
| ENSBTAG00000035710 | *ZBBX* | 1567.8659 | 0.7161 | 0.0016 | 0.0389 |
| ENSBTAG00000006947 | *ROPN1* | 3288.3256 | 0.4893 | 0.0017 | 0.0393 |
| ENSBTAG00000020009 | *CCDC81* | 356.5061 | 0.6143 | 0.0017 | 0.0393 |
| ENSBTAG00000007888 | *EFR3B* | 239.2746 | 0.8888 | 0.0017 | 0.0393 |
| ENSBTAG00000002455 | *KLHL8* | 267.0531 | 0.5424 | 0.0017 | 0.0394 |
| ENSBTAG00000024688 | *PHIP* | 2469.0526 | 0.3179 | 0.0017 | 0.0394 |
| ENSBTAG00000024878 | *ANKRD31* | 97.2371 | 0.9603 | 0.0017 | 0.0402 |
| ENSBTAG00000045744 | *NKRF* | 146.3517 | 0.6512 | 0.0017 | 0.0403 |
| ENSBTAG00000012577 | *UVSSA* | 149.6179 | 0.7226 | 0.0018 | 0.0407 |
| ENSBTAG00000046664 |  | 556.9323 | 0.4673 | 0.0018 | 0.0408 |
| ENSBTAG00000019244 | *P2RX6* | 401.2864 | 0.6930 | 0.0018 | 0.0408 |
| ENSBTAG00000001483 | *SRGAP2* | 515.9717 | 0.3786 | 0.0018 | 0.0408 |
| ENSBTAG00000024662 | *CXorf30* | 1652.6499 | 0.5729 | 0.0018 | 0.0412 |
| ENSBTAG00000034657 |  | 17.8147 | 1.1411 | 0.0018 | 0.0412 |
| ENSBTAG00000038604 | *H2B* | 25.9633 | 1.1457 | 0.0018 | 0.0419 |
| ENSBTAG00000030977 | *ULK2* | 1881.7229 | 0.5330 | 0.0019 | 0.0425 |
| ENSBTAG00000027181 | *LAMA3* | 4302.6966 | 0.8450 | 0.0019 | 0.0434 |
| ENSBTAG00000013698 | *TTC25* | 1673.8922 | 0.3525 | 0.0019 | 0.0438 |
| ENSBTAG00000019197 | *FAM208B* | 1769.0714 | 0.2933 | 0.0020 | 0.0442 |
| ENSBTAG00000003033 | *GADD45G* | 179.0522 | 0.8682 | 0.0020 | 0.0446 |
| ENSBTAG00000021273 | *SEMA4B* | 1428.4224 | 0.4726 | 0.0020 | 0.0448 |
| ENSBTAG00000044158 | *LDLRAD3* | 197.0585 | 0.6648 | 0.0020 | 0.0450 |
| ENSBTAG00000007580 | *ZSWIM2* | 79.7093 | 0.6671 | 0.0020 | 0.0450 |
| ENSBTAG00000021775 | *SLC4A9* | 94.0179 | 0.7110 | 0.0021 | 0.0454 |
| ENSBTAG00000008932 | *SHISA7* | 126.0000 | 0.9784 | 0.0021 | 0.0458 |
| ENSBTAG00000009123 | *ING5* | 251.7541 | 0.4113 | 0.0021 | 0.0460 |
| ENSBTAG00000013866 | *RPS27* | 1156.5439 | 0.2634 | 0.0021 | 0.0461 |
| ENSBTAG00000006592 | *SGSM1* | 642.4764 | 0.5561 | 0.0021 | 0.0464 |
| ENSBTAG00000003329 | *FST* | 67.5472 | 0.9181 | 0.0021 | 0.0466 |
| ENSBTAG00000003819 | *STK36* | 792.9418 | 0.3578 | 0.0022 | 0.0471 |
| ENSBTAG00000023745 | *BANP* | 265.5067 | 0.5174 | 0.0022 | 0.0471 |
| ENSBTAG00000013917 | *SRCAP* | 3905.2209 | 0.1958 | 0.0022 | 0.0475 |
| ENSBTAG00000006227 | *IDH3A* | 724.3699 | 0.3085 | 0.0022 | 0.0476 |
| ENSBTAG00000011106 | *PACRG* | 929.0104 | 0.6289 | 0.0023 | 0.0486 |
| ENSBTAG00000021292 | *ANKFN1* | 405.6114 | 0.6108 | 0.0023 | 0.0487 |
| ENSBTAG00000026247 | *PKHD1L1* | 202.2297 | 0.8987 | 0.0023 | 0.0491 |
| ENSBTAG00000007510 | *PXMP2* | 870.0049 | 0.2889 | 0.0023 | 0.0492 |
| ENSBTAG00000046245 |  | 161.1588 | 0.9288 | 0.0023 | 0.0496 |
| ENSBTAG00000008853 | *HNRNPF* | 9688.6570 | 0.1522 | 0.0023 | 0.0497 |
