# Supplementary material for: Size of the Ovulatory Follicle Dictates Spatial Differences in the Oviductal Transcriptome in Cattle
Source: PLoS One. 2015 Dec 23;10(12):e0145321. doi: 10.1371/journal.pone.0145321 (PMC4689418; doi:10.1371/journal.pone.0145321)
Supplement: S3 Table — (DOCX) [file pone.0145321.s005.docx]

**S3 Table. Bio-project, Bio-sample, Experiment and Run accession numbers of the Raw reads resulted from the RNAseq of ampulla and isthmus samples in the SRA data base.**

| **Region** | **Bio-project** | **Sample** | **Bio-sample** | **Experiment number** | **Run number** |
| --- | --- | --- | --- | --- | --- |
| Ampulla | PRJNA268916 | 617 LF/LCL | SAMN02903326 | SRX646336 | SRR1507226 |
|  |  | 79 LF/LCL | SAMN02903327 | SRX792716 | SRR1688430 |
|  |  | 856 SF/SCL | SAMN02903328 | SRX793271 | SRR1688453 |
|  |  | 489215 SF/SCL | SAMN02903329 | SRX793486 | SRR1688642 |
|  |  | 97 SF/SCL | SAMN02903330 | SRX793512 | SRR1688686 |
| Isthmus | PRJNA269103 | 617 LF/LCL | SAMN03249359 | SRX796310 | SRR1693485 |
|  |  | 79 LF/LCL | SAMN03249358 | SRX794694 | SRR1693483 |
|  |  | 527 LF/LCL | SAMN03249381 | SRX796312 | SRR1693489 |
|  |  | 856 SF/SCL | SAMN03249383 | SRX796428 | SRR1693623 |
|  |  | 489215 SF/SCL | SAMN03249384 | SRX796431 | SRR1693625 |
|  |  | 97 SF/SCL | SAMN03249382 | SRX796423 | SRR1693620 |
