# Supplementary material for: Size of the Ovulatory Follicle Dictates Spatial Differences in the Oviductal Transcriptome in Cattle
Source: PLoS One. 2015 Dec 23;10(12):e0145321. doi: 10.1371/journal.pone.0145321 (PMC4689418; doi:10.1371/journal.pone.0145321)
Supplement: S4 Table — Validation of RNAseq gene expression data by qPCR. qPCR data was analyzed using the same RNAseq animals (n = 5) and using 7 animals for each group (n = 14). (DOCX) [file pone.0145321.s006.docx]

**S4 Table. Log2 Fold change and *P value* of ampulla gene expression in LF/LCL and SF/SCL animals.** Validation of RNAseq gene expression data by qPCR. qPCR data was analyzed using the same RNAseq animals (n=5) and using 7 animals for each group (n=14).

| **Ensembl Id** | **Gene Symbol** | **RNAseq (n=6)** | | **qPCR (n=5)** | | **qPCR (n=14)** | |
| --- | --- | --- | --- | --- | --- | --- | --- |
|  |  | **log2 Fold Change** | ***P* value** | **log2 Fold Change** | ***P* value** | **log2 Fold Change** | ***P* value** |
| ENSBTAG00000011034 | ANGPT2 | 0.896 | 0.078 | 0.798 | 0.454 | 0.031 | 0.309 |
| ENSBTAG00000021811 | ANGPT4 | -0.418 | 0.255 | -0.849 | 0.178 | -0.151 | 0.359 |
| ENSBTAG00000003217 | CADM3 | -2.518 | ˂ 0.001 | -1.355 | 0.091 | -0.949 | 0.109 |
| ENSBTAG00000006161 | C-MET | -0.019 | 0.090 | -0.105 | 0.053 | -0.409 | 0.021 |
| ENSBTAG00000006367 | CTGF | -0.353 | 0.221 | -0.008 | 0.497 | -0.228 | 0.167 |
| ENSBTAG00000017135 | CTSS | -0.693 | 0.026 | -1.184 | 0.059 | -0.311 | 0.185 |
| ENSBTAG00000001060 | CXCR4 | -0.824 | ˂ 0.001 | -1.591 | 0.001 | -3.392 | ˂ 0.001 |
| ENSBTAG00000008096 | EDN1 | -0.010 | NA | -0.005 | 0.496 | -0.498 | 0.209 |
| ENSBTAG00000007159 | ESR1 | -0.547 | 0.006 | -0.713 | 0.082 | -0.221 | 0.146 |
| ENSBTAG00000004498 | ESR2 | -1.147 | NA | -1.425 | 0.040 | -0.283 | 0.400 |
| ENSBTAG00000005745 | HPSE | -1.667 | ˂ 0.001 | -1.020 | 0.260 | -0.401 | 0.062 |
| ENSBTAG00000025441 | HSPA1A | 0.939 | ˂ 0.001 | 0.869 | 0.199 | 0.340 | 0.264 |
| ENSBTAG00000005973 | OVGP1 | 0.065 | 0.679 | 0.990 | 0.318 | 0.986 | 0.172 |
| ENSBTAG00000006065 | PCNA | -0.101 | 0.375 | -0.310 | 0.287 | -0.609 | 0.120 |
| ENSBTAG00000034827 | PDGF | -0.405 | 0.263 | -0.529 | 0.249 | -0.388 | 0.122 |
| ENSBTAG00000024648 | PGR | 0.413 | 0.098 | 0.438 | 0.260 | 0.270 | 0.214 |
| ENSBTAG00000010843 | PGRMC2 | -0.052 | 0.755 | -0.143 | 0.249 | -0.090 | 0.153 |
| ENSBTAG00000003454 | RGS20 | -1.420 | 0.078 | -1.795 | 0.125 | -0.441 | 0.247 |
| ENSBTAG00000005359 | TGFB2 | 0.830 | 0.021 | 0.014 | 0.491 | 0.219 | 0.159 |
| ENSBTAG00000012004 | TGFB3 | 0.535 | 0.333 | 0.380 | 0.233 | 0.501 | 0.131 |
| ENSBTAG00000018035 | TGFBR1 | 0.099 | 0.570 | 0.993 | 0.274 | 0.757 | 0.185 |
| ENSBTAG00000019832 | TGFBR2 | -0.261 | NA | -0.706 | 0.115 | -1.199 | 0.206 |
| ENSBTAG00000021879 | VCL | -0.491 | 0.001 | -0.172 | 0.015 | -2.426 | 0.003 |
