# Supplementary material for: Size of the Ovulatory Follicle Dictates Spatial Differences in the Oviductal Transcriptome in Cattle
Source: PLoS One. 2015 Dec 23;10(12):e0145321. doi: 10.1371/journal.pone.0145321 (PMC4689418; doi:10.1371/journal.pone.0145321)
Supplement: S5 Table — (DOCX) [file pone.0145321.s007.docx]

**S5 Table. Differentially expressed genes in isthmus samples (p-adjusted < 0.1) detected by RNAseq.** Respective expression profiles and Log2fold-changes for both treatments LF/LCL and SF/SCL.

| **ENSemBLe ID** | **Gene Symbol** | **Base Mean** | **log2 Fold Change** | ***P* value** | ***P* adj** |
| --- | --- | --- | --- | --- | --- |
| Upregulated LF/LCL |  |  |  |  |  |
| ENSBTAG00000019628 | *EDAR* | 59.1746 | -3.1946 | 0.0000 | 0.0000 |
| ENSBTAG00000000066 | *LRRN4* | 149.1370 | -3.1512 | 0.0000 | 0.0000 |
| ENSBTAG00000032687 |  | 28.2513 | -2.1422 | 0.0000 | 0.0000 |
| ENSBTAG00000047990 |  | 14.8548 | -2.0917 | 0.0000 | 0.0001 |
| ENSBTAG00000004555 | *LRP2* | 7139.6003 | -2.0401 | 0.0000 | 0.0000 |
| ENSBTAG00000003349 | *JAKMIP2* | 160.7114 | -2.0368 | 0.0000 | 0.0000 |
| ENSBTAG00000018287 | *SLC25A48* | 27.2121 | -2.0320 | 0.0000 | 0.0001 |
| ENSBTAG00000021795 | *HNF1A* | 14.5770 | -1.9992 | 0.0000 | 0.0003 |
| ENSBTAG00000007273 | *TF* | 93.6616 | -1.9715 | 0.0000 | 0.0000 |
| ENSBTAG00000012393 | *AGT* | 10600.0419 | -1.8683 | 0.0000 | 0.0000 |
| ENSBTAG00000018253 | *CHRNA1* | 46.6438 | -1.8420 | 0.0000 | 0.0009 |
| ENSBTAG00000009294 | *DEGS2* | 73.4685 | -1.8261 | 0.0000 | 0.0002 |
| ENSBTAG00000019293 | *EGR3* | 14.4243 | -1.8172 | 0.0000 | 0.0005 |
| ENSBTAG00000006961 | *NLRP13* | 27.8191 | -1.7513 | 0.0000 | 0.0026 |
| ENSBTAG00000011634 | *ACPP* | 428.9096 | -1.7414 | 0.0000 | 0.0011 |
| ENSBTAG00000023270 | *CDH19* | 64.5477 | -1.7235 | 0.0000 | 0.0010 |
| ENSBTAG00000019249 | *HS3ST6* | 210.3190 | -1.7006 | 0.0000 | 0.0008 |
| ENSBTAG00000046350 | *PKP1* | 344.3445 | -1.6926 | 0.0000 | 0.0000 |
| ENSBTAG00000005941 | *TRAV8-1* | 16.3965 | -1.6797 | 0.0000 | 0.0035 |
| ENSBTAG00000015462 | *BNC1* | 49.7435 | -1.6683 | 0.0000 | 0.0000 |
| ENSBTAG00000018481 |  | 18.2064 | -1.6503 | 0.0001 | 0.0048 |
| ENSBTAG00000039483 | *DSG3* | 69.4841 | -1.6436 | 0.0000 | 0.0026 |
| ENSBTAG00000000376 | *P2RX3* | 85.4910 | -1.6389 | 0.0001 | 0.0048 |
| ENSBTAG00000046158 | *CFB* | 1948.6683 | -1.5982 | 0.0000 | 0.0002 |
| ENSBTAG00000048170 | *PCDHA11* | 19.6034 | -1.5733 | 0.0000 | 0.0013 |
| ENSBTAG00000007635 | *PLCL1* | 351.4433 | -1.5654 | 0.0000 | 0.0000 |
| ENSBTAG00000010024 | *MMP24* | 27.4037 | -1.5637 | 0.0000 | 0.0035 |
| ENSBTAG00000017561 | *HHIPL2* | 99.5027 | -1.5622 | 0.0000 | 0.0002 |
| ENSBTAG00000000169 | *ASZ1* | 8.4262 | -1.5585 | 0.0001 | 0.0088 |
| ENSBTAG00000044119 | *KSR2* | 268.1367 | -1.5520 | 0.0000 | 0.0002 |
| ENSBTAG00000009093 | *GAL3ST2* | 176.9975 | -1.5327 | 0.0001 | 0.0078 |
| ENSBTAG00000017056 | *PKLR* | 26.6185 | -1.5285 | 0.0000 | 0.0014 |
| ENSBTAG00000020793 | *KCNA4* | 18.3596 | -1.5280 | 0.0001 | 0.0078 |
| ENSBTAG00000010245 | *SPRY3* | 102.1367 | -1.5199 | 0.0003 | 0.0132 |
| ENSBTAG00000017793 | *KLRG2* | 30.8096 | -1.5191 | 0.0000 | 0.0029 |
| ENSBTAG00000013689 | *MCTP2* | 183.4138 | -1.5186 | 0.0000 | 0.0002 |
| ENSBTAG00000021906 | *COL6A5* | 64.0030 | -1.5133 | 0.0001 | 0.0063 |
| ENSBTAG00000000011 | *TDH* | 67.8576 | -1.5014 | 0.0000 | 0.0032 |
| ENSBTAG00000046649 | *SGPP2* | 124.4971 | -1.4898 | 0.0001 | 0.0067 |
| ENSBTAG00000017508 | *CYSLTR2* | 44.7548 | -1.4838 | 0.0000 | 0.0033 |
| ENSBTAG00000015307 | *FBN2* | 1125.1300 | -1.4664 | 0.0000 | 0.0008 |
| ENSBTAG00000019385 | *SCN3A* | 54.3116 | -1.4648 | 0.0003 | 0.0158 |
| ENSBTAG00000010103 | *TRIM9* | 305.4035 | -1.4639 | 0.0001 | 0.0078 |
| ENSBTAG00000004680 | *SLC13A5* | 892.3268 | -1.4623 | 0.0003 | 0.0158 |
| ENSBTAG00000014470 | *ALDH1L1* | 32.4247 | -1.4617 | 0.0004 | 0.0169 |
| ENSBTAG00000012503 | *MGAT3* | 520.1896 | -1.4604 | 0.0001 | 0.0050 |
| ENSBTAG00000014906 | *VCAN* | 1705.8621 | -1.4454 | 0.0000 | 0.0000 |
| ENSBTAG00000007378 | *CLIP4* | 238.6427 | -1.4439 | 0.0000 | 0.0002 |
| ENSBTAG00000046193 |  | 115.4764 | -1.4173 | 0.0000 | 0.0006 |
| ENSBTAG00000030286 | *GABRA1* | 7.0293 | -1.4150 | 0.0006 | 0.0221 |
| ENSBTAG00000015485 | *MGC134132* | 958.9890 | -1.4103 | 0.0000 | 0.0000 |
| ENSBTAG00000037971 |  | 13.8773 | -1.4095 | 0.0006 | 0.0236 |
| ENSBTAG00000012837 | *COL6A6* | 103.4770 | -1.3950 | 0.0003 | 0.0156 |
| ENSBTAG00000046058 | *RNASE11* | 35.3678 | -1.3923 | 0.0008 | 0.0283 |
| ENSBTAG00000021310 | *COL4A4* | 177.4830 | -1.3814 | 0.0005 | 0.0204 |
| ENSBTAG00000017069 | *FAM198B* | 355.7556 | -1.3796 | 0.0001 | 0.0067 |
| ENSBTAG00000009642 | *AKAP5* | 350.7083 | -1.3785 | 0.0000 | 0.0000 |
| ENSBTAG00000008380 | *ITGA11* | 197.0774 | -1.3741 | 0.0001 | 0.0054 |
| ENSBTAG00000009637 | *SLC12A2* | 1311.1467 | -1.3708 | 0.0000 | 0.0001 |
| ENSBTAG00000010522 |  | 15.0008 | -1.3646 | 0.0010 | 0.0312 |
| ENSBTAG00000021829 | *GM2A* | 5480.7558 | -1.3633 | 0.0000 | 0.0024 |
| ENSBTAG00000021066 | *CAPN11* | 11.0901 | -1.3612 | 0.0009 | 0.0290 |
| ENSBTAG00000014225 | *SLC5A9* | 555.2315 | -1.3553 | 0.0000 | 0.0002 |
| ENSBTAG00000008556 | *POU2F2* | 37.5462 | -1.3492 | 0.0005 | 0.0207 |
| ENSBTAG00000037686 | *SRPX2* | 48.9065 | -1.3484 | 0.0002 | 0.0107 |
| ENSBTAG00000001728 | *IGSF10* | 1678.5445 | -1.3473 | 0.0000 | 0.0002 |
| ENSBTAG00000001206 | *LCN15* | 34.3117 | -1.3440 | 0.0006 | 0.0221 |
| ENSBTAG00000033197 | *EPDR1* | 51.4914 | -1.3438 | 0.0000 | 0.0005 |
| ENSBTAG00000025210 | *COL4A2* | 12154.7381 | -1.3379 | 0.0000 | 0.0000 |
| ENSBTAG00000001051 | *OSCAR* | 39.6620 | -1.3319 | 0.0006 | 0.0221 |
| ENSBTAG00000046409 | *EGR2* | 15.0043 | -1.3318 | 0.0010 | 0.0320 |
| ENSBTAG00000033726 | *GRIP1* | 255.7079 | -1.3290 | 0.0000 | 0.0034 |
| ENSBTAG00000011651 | *HECTD2* | 180.0809 | -1.3285 | 0.0000 | 0.0001 |
| ENSBTAG00000010719 | *ANGPTL1* | 250.5856 | -1.3275 | 0.0005 | 0.0197 |
| ENSBTAG00000045986 | *MIA2* | 97.3066 | -1.3255 | 0.0012 | 0.0355 |
| ENSBTAG00000003751 | *MACC1* | 20.5862 | -1.3154 | 0.0016 | 0.0433 |
| ENSBTAG00000015926 | *ABLIM2* | 539.0715 | -1.3120 | 0.0000 | 0.0000 |
| ENSBTAG00000046476 | *IGSF1* | 322.5467 | -1.3085 | 0.0001 | 0.0054 |
| ENSBTAG00000038849 | *TRDN* | 5.1073 | -1.3029 | 0.0018 | 0.0465 |
| ENSBTAG00000045704 | *IGSF11* | 132.4502 | -1.3007 | 0.0005 | 0.0204 |
| ENSBTAG00000004884 | *SLC4A5* | 13.1203 | -1.2969 | 0.0018 | 0.0480 |
| ENSBTAG00000004574 | *UGT8* | 78.6907 | -1.2949 | 0.0008 | 0.0273 |
| ENSBTAG00000010501 | *SELV* | 20.0054 | -1.2889 | 0.0006 | 0.0221 |
| ENSBTAG00000001321 | *IL1B* | 12.0340 | -1.2834 | 0.0015 | 0.0429 |
| ENSBTAG00000021938 | *ARHGAP44* | 1135.9490 | -1.2763 | 0.0000 | 0.0008 |
| ENSBTAG00000003625 | *VSTM2A* | 6.4410 | -1.2648 | 0.0012 | 0.0368 |
| ENSBTAG00000009800 | *MSI1* | 38.6894 | -1.2576 | 0.0003 | 0.0141 |
| ENSBTAG00000007680 | *THSD7A* | 341.6018 | -1.2562 | 0.0013 | 0.0388 |
| ENSBTAG00000000707 | *WISP1* | 31.7954 | -1.2553 | 0.0010 | 0.0312 |
| ENSBTAG00000046924 | *SEMA3G* | 515.7532 | -1.2507 | 0.0002 | 0.0116 |
| ENSBTAG00000014456 | *ERN2* | 1437.2063 | -1.2507 | 0.0005 | 0.0197 |
| ENSBTAG00000020352 | *PAQR5* | 27.8635 | -1.2493 | 0.0005 | 0.0204 |
| ENSBTAG00000006110 | *MCTP1* | 70.5753 | -1.2382 | 0.0001 | 0.0065 |
| ENSBTAG00000007602 | *ITGA8* | 3180.4557 | -1.2373 | 0.0000 | 0.0000 |
| ENSBTAG00000010877 | *ARMC12* | 161.8132 | -1.2358 | 0.0001 | 0.0053 |
| ENSBTAG00000011864 | *RGMB* | 1860.8478 | -1.2291 | 0.0000 | 0.0031 |
| ENSBTAG00000013210 | *ADAMTS4* | 227.3293 | -1.2288 | 0.0000 | 0.0012 |
| ENSBTAG00000020979 | *NGFR* | 59.1092 | -1.2245 | 0.0002 | 0.0103 |
| ENSBTAG00000009260 | *GPM6B* | 945.8118 | -1.2166 | 0.0000 | 0.0004 |
| ENSBTAG00000013439 | *ARHGEF26* | 598.9214 | -1.2087 | 0.0000 | 0.0001 |
| ENSBTAG00000014548 | *FAM129A* | 2930.0453 | -1.2083 | 0.0003 | 0.0132 |
| ENSBTAG00000021699 | *RORB* | 272.2293 | -1.2078 | 0.0014 | 0.0402 |
| ENSBTAG00000019694 | *ADAM23* | 52.6462 | -1.2063 | 0.0004 | 0.0181 |
| ENSBTAG00000022886 | *RYR2* | 310.3003 | -1.1981 | 0.0000 | 0.0007 |
| ENSBTAG00000013662 | *COL8A1* | 565.0094 | -1.1980 | 0.0001 | 0.0054 |
| ENSBTAG00000023369 | *GRIN2D* | 26.5981 | -1.1912 | 0.0020 | 0.0498 |
| ENSBTAG00000016913 | *AVIL* | 78.8297 | -1.1877 | 0.0007 | 0.0241 |
| ENSBTAG00000011833 | *GRIA3* | 368.5308 | -1.1871 | 0.0001 | 0.0077 |
| ENSBTAG00000008160 | *MBOAT2* | 1579.0915 | -1.1854 | 0.0001 | 0.0079 |
| ENSBTAG00000021957 | *LTBP2* | 3548.5392 | -1.1832 | 0.0000 | 0.0000 |
| ENSBTAG00000017882 | *LRRC3* | 272.2474 | -1.1739 | 0.0001 | 0.0057 |
| ENSBTAG00000008338 | *PLCB1* | 552.2250 | -1.1697 | 0.0000 | 0.0010 |
| ENSBTAG00000013942 | *DAGLA* | 673.5407 | -1.1610 | 0.0005 | 0.0216 |
| ENSBTAG00000021904 | *KIF26A* | 382.0237 | -1.1558 | 0.0000 | 0.0028 |
| ENSBTAG00000030173 | *QRICH2* | 139.9512 | -1.1553 | 0.0003 | 0.0142 |
| ENSBTAG00000000520 | *ATP2B3* | 85.5717 | -1.1479 | 0.0003 | 0.0150 |
| ENSBTAG00000001219 |  | 839.3549 | -1.1400 | 0.0001 | 0.0082 |
| ENSBTAG00000040296 | *GPR21* | 45.8713 | -1.1351 | 0.0006 | 0.0227 |
| ENSBTAG00000048067 | *C17orf96* | 56.3148 | -1.1233 | 0.0008 | 0.0265 |
| ENSBTAG00000006703 | *PTGDR* | 50.0895 | -1.1231 | 0.0002 | 0.0103 |
| ENSBTAG00000000061 | *PCDH7* | 863.3215 | -1.1185 | 0.0001 | 0.0083 |
| ENSBTAG00000009230 | *FBLN7* | 81.3381 | -1.1144 | 0.0015 | 0.0420 |
| ENSBTAG00000011752 | *SYNM* | 13433.4009 | -1.1143 | 0.0002 | 0.0095 |
| ENSBTAG00000011121 | *CLCN4* | 656.2222 | -1.1141 | 0.0000 | 0.0002 |
| ENSBTAG00000044126 | *SNTB1* | 1450.6636 | -1.1114 | 0.0000 | 0.0000 |
| ENSBTAG00000002299 | *SEL1L3* | 3289.6737 | -1.1031 | 0.0003 | 0.0159 |
| ENSBTAG00000014665 | *ADAMTS2* | 1031.7482 | -1.1006 | 0.0000 | 0.0003 |
| ENSBTAG00000015226 | *BRSK1* | 281.4101 | -1.0813 | 0.0009 | 0.0299 |
| ENSBTAG00000013760 | *COL4A6* | 903.6287 | -1.0777 | 0.0007 | 0.0243 |
| ENSBTAG00000008272 | *EBF3* | 45.6544 | -1.0748 | 0.0008 | 0.0281 |
| ENSBTAG00000011082 | *IGF-I* | 1277.0415 | -1.0744 | 0.0004 | 0.0163 |
| ENSBTAG00000003130 | *CHRNA3* | 609.3571 | -1.0692 | 0.0002 | 0.0127 |
| ENSBTAG00000012849 | *COL4A1* | 18875.1905 | -1.0553 | 0.0002 | 0.0092 |
| ENSBTAG00000007705 | *COLEC12* | 1454.4519 | -1.0553 | 0.0000 | 0.0021 |
| ENSBTAG00000011847 | *ASPN* | 2579.2481 | -1.0535 | 0.0000 | 0.0009 |
| ENSBTAG00000011966 | *LAMC1* | 6033.7051 | -1.0484 | 0.0006 | 0.0225 |
| ENSBTAG00000011766 | *C7* | 3322.5213 | -1.0474 | 0.0000 | 0.0005 |
| ENSBTAG00000006688 | *RPS6KL1* | 79.1439 | -1.0346 | 0.0000 | 0.0028 |
| ENSBTAG00000002092 | *PI16* | 4160.2184 | -1.0321 | 0.0000 | 0.0002 |
| ENSBTAG00000013103 | *COL1A1* | 68421.5894 | -1.0309 | 0.0000 | 0.0009 |
| ENSBTAG00000025345 | *ARL10* | 82.0886 | -1.0302 | 0.0007 | 0.0252 |
| ENSBTAG00000027020 | *COL5A2* | 7523.9675 | -1.0276 | 0.0000 | 0.0000 |
| ENSBTAG00000014824 | *MMP14* | 3990.5060 | -1.0257 | 0.0002 | 0.0127 |
| ENSBTAG00000004231 | *GPM6A* | 249.7212 | -1.0220 | 0.0004 | 0.0169 |
| ENSBTAG00000021466 | *COL3A1* | 114537.5802 | -1.0136 | 0.0000 | 0.0000 |
| ENSBTAG00000004017 | *PLEKHG2* | 864.5345 | -1.0118 | 0.0000 | 0.0006 |
| ENSBTAG00000019674 | *CLIP3* | 665.2771 | -1.0111 | 0.0000 | 0.0000 |
| ENSBTAG00000022244 |  | 46.9680 | -1.0098 | 0.0016 | 0.0445 |
| ENSBTAG00000004952 | *MFSD4* | 220.6352 | -1.0078 | 0.0004 | 0.0181 |
| ENSBTAG00000003069 | *MAN1C1* | 536.6834 | -1.0072 | 0.0000 | 0.0006 |
| ENSBTAG00000019294 | *ABCC9* | 933.9334 | -1.0017 | 0.0010 | 0.0312 |
| ENSBTAG00000008300 | *FN1* | 13362.8297 | -0.9995 | 0.0007 | 0.0262 |
| ENSBTAG00000006326 | *ALDH1L2* | 359.7095 | -0.9988 | 0.0005 | 0.0207 |
| ENSBTAG00000007753 | *KIFC2* | 404.5943 | -0.9971 | 0.0000 | 0.0031 |
| ENSBTAG00000017086 | *GRB10* | 807.7420 | -0.9898 | 0.0000 | 0.0009 |
| ENSBTAG00000011454 | *FKBP10* | 1029.7140 | -0.9892 | 0.0000 | 0.0002 |
| ENSBTAG00000008291 | *PROCR* | 260.5112 | -0.9842 | 0.0007 | 0.0240 |
| ENSBTAG00000047613 | *HTRA3* | 1461.5444 | -0.9833 | 0.0008 | 0.0285 |
| ENSBTAG00000019421 | *DACT1* | 442.0101 | -0.9816 | 0.0001 | 0.0065 |
| ENSBTAG00000043975 | *MYCT1* | 186.5456 | -0.9757 | 0.0000 | 0.0002 |
| ENSBTAG00000015405 | *DCHS1* | 2063.7540 | -0.9752 | 0.0001 | 0.0048 |
| ENSBTAG00000001599 | *SV2A* | 323.2768 | -0.9712 | 0.0001 | 0.0060 |
| ENSBTAG00000013176 | *SMOC2* | 571.4027 | -0.9700 | 0.0003 | 0.0140 |
| ENSBTAG00000011882 | *SLC24A3* | 1002.4633 | -0.9679 | 0.0000 | 0.0000 |
| ENSBTAG00000010179 | *COL5A3* | 1022.3213 | -0.9670 | 0.0005 | 0.0204 |
| ENSBTAG00000004571 | *PRPF4* | 3189.8675 | -0.9660 | 0.0005 | 0.0201 |
| ENSBTAG00000013674 | *EDNRA* | 777.7496 | -0.9608 | 0.0003 | 0.0127 |
| ENSBTAG00000000483 | *HYAL1* | 332.6538 | -0.9592 | 0.0000 | 0.0038 |
| ENSBTAG00000011324 | *EMILIN1* | 18704.0233 | -0.9557 | 0.0000 | 0.0023 |
| ENSBTAG00000046879 |  | 200.6018 | -0.9511 | 0.0009 | 0.0305 |
| ENSBTAG00000012994 | *LOX* | 659.1987 | -0.9501 | 0.0003 | 0.0128 |
| ENSBTAG00000013300 | *KCNMA1* | 4749.2785 | -0.9460 | 0.0000 | 0.0007 |
| ENSBTAG00000002626 | *ARHGAP6* | 604.6730 | -0.9448 | 0.0008 | 0.0268 |
| ENSBTAG00000024560 | *MGC151592* | 241.0746 | -0.9424 | 0.0000 | 0.0006 |
| ENSBTAG00000016197 | *PHLDB1* | 1788.9330 | -0.9343 | 0.0000 | 0.0029 |
| ENSBTAG00000032152 | *TMPRSS6* | 134.8077 | -0.9318 | 0.0000 | 0.0004 |
| ENSBTAG00000001745 | *LUM* | 6074.3388 | -0.9253 | 0.0000 | 0.0040 |
| ENSBTAG00000007214 | *ELL2* | 657.0030 | -0.9204 | 0.0000 | 0.0027 |
| ENSBTAG00000037937 |  | 237.6732 | -0.9181 | 0.0002 | 0.0106 |
| ENSBTAG00000020342 | *MYOC* | 629.6237 | -0.8986 | 0.0005 | 0.0204 |
| ENSBTAG00000010161 | *CCL21* | 487.0782 | -0.8981 | 0.0013 | 0.0394 |
| ENSBTAG00000017258 | *ACSL3* | 3368.2042 | -0.8969 | 0.0000 | 0.0000 |
| ENSBTAG00000006029 | *OGDH* | 9873.7062 | -0.8955 | 0.0007 | 0.0256 |
| ENSBTAG00000014567 | *MYLK* | 59135.8104 | -0.8861 | 0.0000 | 0.0002 |
| ENSBTAG00000020313 | *FNBP1* | 5586.4608 | -0.8847 | 0.0000 | 0.0019 |
| ENSBTAG00000001274 | *PPM1L* | 114.4339 | -0.8817 | 0.0009 | 0.0304 |
| ENSBTAG00000019839 | *LTBP1* | 10086.9724 | -0.8787 | 0.0001 | 0.0048 |
| ENSBTAG00000013366 | *NRIP3* | 111.6989 | -0.8767 | 0.0002 | 0.0122 |
| ENSBTAG00000007415 | *SLC7A8* | 318.7245 | -0.8766 | 0.0006 | 0.0221 |
| ENSBTAG00000048237 | *FGF9* | 74.1884 | -0.8759 | 0.0004 | 0.0160 |
| ENSBTAG00000008997 | *ENG* | 1219.3669 | -0.8695 | 0.0000 | 0.0009 |
| ENSBTAG00000012441 | *SMAD7* | 171.8805 | -0.8690 | 0.0012 | 0.0352 |
| ENSBTAG00000017808 | *ARSJ* | 543.9303 | -0.8665 | 0.0001 | 0.0079 |
| ENSBTAG00000015646 | *RASSF3* | 263.6037 | -0.8658 | 0.0002 | 0.0106 |
| ENSBTAG00000016707 | *FKBP9* | 7373.5713 | -0.8657 | 0.0000 | 0.0010 |
| ENSBTAG00000010379 | *CRTC1* | 334.0471 | -0.8645 | 0.0000 | 0.0001 |
| ENSBTAG00000019382 | *PKDCC* | 1722.7340 | -0.8598 | 0.0010 | 0.0312 |
| ENSBTAG00000004337 | *PDE1B* | 204.7215 | -0.8597 | 0.0009 | 0.0299 |
| ENSBTAG00000004840 | *C1S* | 10601.0366 | -0.8547 | 0.0003 | 0.0151 |
| ENSBTAG00000010472 | *TNFAIP8L3* | 217.4814 | -0.8502 | 0.0017 | 0.0457 |
| ENSBTAG00000045868 | *ZNF135* | 131.7828 | -0.8446 | 0.0008 | 0.0286 |
| ENSBTAG00000007141 | *GULP1* | 205.3255 | -0.8434 | 0.0016 | 0.0431 |
| ENSBTAG00000009917 | *PIP5K1B* | 164.6375 | -0.8336 | 0.0019 | 0.0493 |
| ENSBTAG00000020854 | *BCL6B* | 222.2746 | -0.8312 | 0.0002 | 0.0098 |
| ENSBTAG00000002052 | *PLOD1* | 4672.0403 | -0.8306 | 0.0014 | 0.0408 |
| ENSBTAG00000012252 | *MOCOS* | 3219.2837 | -0.8267 | 0.0010 | 0.0322 |
| ENSBTAG00000011403 | *RUSC2* | 863.9682 | -0.8259 | 0.0001 | 0.0088 |
| ENSBTAG00000015549 | *PCDH18* | 993.0946 | -0.8257 | 0.0006 | 0.0234 |
| ENSBTAG00000004612 | *SORCS3* | 368.7831 | -0.8197 | 0.0007 | 0.0243 |
| ENSBTAG00000046176 | *SPEG* | 2078.4718 | -0.8188 | 0.0015 | 0.0428 |
| ENSBTAG00000030247 | *NSMF* | 844.5642 | -0.8158 | 0.0006 | 0.0234 |
| ENSBTAG00000010793 | *CCDC80* | 11835.1650 | -0.8106 | 0.0010 | 0.0312 |
| ENSBTAG00000001077 | *OSBPL5* | 1886.6635 | -0.8084 | 0.0000 | 0.0002 |
| ENSBTAG00000017071 | *C1QTNF3* | 957.5916 | -0.8035 | 0.0001 | 0.0058 |
| ENSBTAG00000015991 | *E-CADHERIN* | 3868.7743 | -0.7879 | 0.0000 | 0.0000 |
| ENSBTAG00000031277 | *FOXL2* | 149.5685 | -0.7862 | 0.0019 | 0.0490 |
| ENSBTAG00000003199 | *SLC35C1* | 373.9324 | -0.7826 | 0.0017 | 0.0465 |
| ENSBTAG00000012088 | *FBLN1* | 20227.6810 | -0.7826 | 0.0003 | 0.0157 |
| ENSBTAG00000013347 | *DMPK* | 7718.2365 | -0.7708 | 0.0010 | 0.0312 |
| ENSBTAG00000005724 | *SYTL4* | 522.7055 | -0.7663 | 0.0001 | 0.0074 |
| ENSBTAG00000021830 | *ENPP1* | 2379.4641 | -0.7642 | 0.0000 | 0.0032 |
| ENSBTAG000000103891 | *FAM47E-STBD1* | 353.4136 | -0.7635 | 0.0008 | 0.0262 |
| ENSBTAG00000002451 | *TTC39A* | 868.7552 | -0.7624 | 0.0014 | 0.0404 |
| ENSBTAG00000001209 | *PHLDB2* | 2910.8550 | -0.7520 | 0.0000 | 0.0037 |
| ENSBTAG00000003219 | *FBXL7* | 441.1153 | -0.7504 | 0.0007 | 0.0240 |
| ENSBTAG00000004291 | *SEPT6* | 897.2747 | -0.7464 | 0.0005 | 0.0198 |
| ENSBTAG00000015844 | *TFPI2* | 4302.0169 | -0.7398 | 0.0015 | 0.0428 |
| ENSBTAG00000013953 | *CALD1* | 12844.3863 | -0.7313 | 0.0002 | 0.0090 |
| ENSBTAG00000006161 | *MET* | 1372.1189 | -0.7300 | 0.0000 | 0.0002 |
| ENSBTAG00000020455 | *ITPR1* | 6126.7174 | -0.7231 | 0.0000 | 0.0038 |
| ENSBTAG00000006686 | *NPNT* | 504.5204 | -0.7231 | 0.0003 | 0.0156 |
| ENSBTAG00000010050 | *COL16A1* | 4977.2619 | -0.7198 | 0.0014 | 0.0406 |
| ENSBTAG00000019269 | *COL6A2* | 23355.5194 | -0.7137 | 0.0011 | 0.0340 |
| ENSBTAG00000005633 | *RGNEF* | 1294.1358 | -0.7117 | 0.0009 | 0.0309 |
| ENSBTAG00000019375 | *SCARF2* | 450.3587 | -0.7103 | 0.0014 | 0.0404 |
| ENSBTAG00000019024 | *JAZF1* | 1779.0993 | -0.7065 | 0.0019 | 0.0493 |
| ENSBTAG00000012777 | *SRF* | 2978.5624 | -0.7011 | 0.0003 | 0.0127 |
| ENSBTAG00000020199 | *F2R* | 545.7639 | -0.7002 | 0.0018 | 0.0466 |
| ENSBTAG00000003835 | *BMP4* | 534.8739 | -0.6924 | 0.0017 | 0.0465 |
| ENSBTAG00000023907 | *COL18A1* | 6878.1470 | -0.6879 | 0.0000 | 0.0007 |
| ENSBTAG00000034827 | *PDGFD* | 196.9771 | -0.6818 | 0.0005 | 0.0204 |
| ENSBTAG00000014376 | *ANKRD13A* | 2992.2928 | -0.6764 | 0.0001 | 0.0053 |
| ENSBTAG00000037649 | *VIPR2* | 1856.1644 | -0.6729 | 0.0004 | 0.0185 |
| ENSBTAG00000010462 | *ROBO2* | 412.3655 | -0.6714 | 0.0010 | 0.0318 |
| ENSBTAG00000009181 | *INPP5A* | 3020.9740 | -0.6686 | 0.0000 | 0.0011 |
| ENSBTAG00000019473 | *IGLON5* | 267.1810 | -0.6496 | 0.0012 | 0.0361 |
| ENSBTAG00000017958 | *AHI1* | 1563.0007 | -0.6477 | 0.0000 | 0.0001 |
| ENSBTAG00000000900 | *LRRC8C* | 331.4635 | -0.6416 | 0.0010 | 0.0325 |
| ENSBTAG00000005668 | *SLC39A8* | 361.3316 | -0.6328 | 0.0015 | 0.0420 |
| ENSBTAG00000032859 |  | 291.4352 | -0.6306 | 0.0010 | 0.0314 |
| ENSBTAG00000044017 | *MSRB3* | 1242.7115 | -0.6248 | 0.0000 | 0.0024 |
| ENSBTAG00000001361 | *NMNAT1* | 369.9392 | -0.6235 | 0.0001 | 0.0058 |
| ENSBTAG00000004893 | *ACOX3* | 808.2175 | -0.6083 | 0.0001 | 0.0065 |
| ENSBTAG00000037757 | *EBF4* | 328.2417 | -0.6012 | 0.0018 | 0.0479 |
| ENSBTAG00000008411 | *PLEKHO2* | 971.2244 | -0.5992 | 0.0002 | 0.0100 |
| ENSBTAG00000009835 | *CACNA1G* | 1311.3867 | -0.5908 | 0.0001 | 0.0074 |
| ENSBTAG00000011913 | *CKAP4* | 2147.5625 | -0.5857 | 0.0007 | 0.0259 |
| ENSBTAG00000016714 | *RGS12* | 284.4980 | -0.5823 | 0.0014 | 0.0403 |
| ENSBTAG00000015584 | *BMI1* | 577.5099 | -0.5786 | 0.0005 | 0.0203 |
| ENSBTAG00000005617 | *SLC41A3* | 319.6904 | -0.5742 | 0.0018 | 0.0465 |
| ENSBTAG00000009470 | *CLIC4* | 6486.7368 | -0.5695 | 0.0005 | 0.0204 |
| ENSBTAG00000010529 | *FZD6* | 2286.9283 | -0.5676 | 0.0003 | 0.0141 |
| ENSBTAG00000011190 | *FLNA* | 155519.9688 | -0.5620 | 0.0018 | 0.0465 |
| ENSBTAG00000032051 |  | 1905.8507 | -0.5604 | 0.0000 | 0.0027 |
| ENSBTAG00000001509 | *ELK3* | 1606.8569 | -0.5223 | 0.0009 | 0.0300 |
| ENSBTAG00000005865 | *MAPK6* | 2190.7011 | -0.5156 | 0.0002 | 0.0122 |
| ENSBTAG00000016813 | *SH3D19* | 3234.5838 | -0.5109 | 0.0001 | 0.0061 |
| ENSBTAG00000010971 | *MGC142792* | 1298.6388 | -0.4937 | 0.0003 | 0.0159 |
| ENSBTAG00000009346 | *MAPKAPK5* | 1093.1976 | -0.4841 | 0.0015 | 0.0420 |
| ENSBTAG00000009061 | *FAR1* | 2025.9671 | -0.4839 | 0.0000 | 0.0039 |
| ENSBTAG00000010230 | *CAPN1* | 4497.8706 | -0.4693 | 0.0008 | 0.0265 |
| ENSBTAG00000018310 | *SETD3* | 3299.9906 | -0.4675 | 0.0007 | 0.0259 |
| ENSBTAG00000010534 | *M-RIP* | 8299.6067 | -0.4641 | 0.0012 | 0.0358 |
| ENSBTAG00000006740 | *SCYL1* | 1917.3259 | -0.4331 | 0.0014 | 0.0408 |
|  |  |  |  |  |  |
| Upregulated SF/SCL |  |  |  |  |  |
| ENSBTAG00000009127 | *TSPYL4* | 8971.2831 | 0.3691 | 0.0015 | 0.0429 |
| ENSBTAG00000037516 |  | 11663.9451 | 0.3733 | 0.0019 | 0.0488 |
| ENSBTAG00000010885 | *TSPYL1* | 7928.1365 | 0.3756 | 0.0012 | 0.0367 |
| ENSBTAG00000031747 | *HMGN4* | 1781.2975 | 0.3881 | 0.0010 | 0.0327 |
| ENSBTAG00000013782 | *DNAJB2* | 1700.3813 | 0.3919 | 0.0017 | 0.0465 |
| ENSBTAG00000011285 | *TRMT1L* | 2347.5559 | 0.3958 | 0.0010 | 0.0312 |
| ENSBTAG00000017245 | *COPG2* | 2181.9871 | 0.3996 | 0.0020 | 0.0498 |
| ENSBTAG00000017713 | *KTN1* | 6496.8749 | 0.4122 | 0.0006 | 0.0228 |
| ENSBTAG00000030632 | *ALG10* | 1443.3463 | 0.4205 | 0.0005 | 0.0210 |
| ENSBTAG00000015591 | *SQSTM1* | 10562.8755 | 0.4239 | 0.0018 | 0.0465 |
| ENSBTAG00000013069 | *IQCB1* | 1048.8267 | 0.4245 | 0.0019 | 0.0486 |
| ENSBTAG00000030881 | *TMEM260* | 2271.3801 | 0.4344 | 0.0016 | 0.0432 |
| ENSBTAG00000006101 | *PSMD4* | 4132.2681 | 0.4636 | 0.0006 | 0.0237 |
| ENSBTAG00000010091 | *ARL6* | 1063.3913 | 0.4679 | 0.0016 | 0.0437 |
| ENSBTAG00000017395 | *LASS5* | 2059.5262 | 0.4710 | 0.0010 | 0.0314 |
| ENSBTAG00000021103 | *SLC35F5* | 3746.8995 | 0.4715 | 0.0003 | 0.0150 |
| ENSBTAG00000023963 | *RHBDD1* | 707.4584 | 0.4801 | 0.0013 | 0.0385 |
| ENSBTAG00000030956 | *ZSCAN21* | 2198.1761 | 0.4812 | 0.0004 | 0.0185 |
| ENSBTAG00000020040 | *LPCAT4* | 3454.0385 | 0.4881 | 0.0015 | 0.0428 |
| ENSBTAG00000015718 | *CASP8* | 1434.7169 | 0.4957 | 0.0015 | 0.0415 |
| ENSBTAG00000008884 | *MAPRE3* | 1829.9397 | 0.4963 | 0.0016 | 0.0447 |
| ENSBTAG00000016470 | *NADSYN1* | 661.9386 | 0.5014 | 0.0014 | 0.0404 |
| ENSBTAG00000006213 | *IFT27* | 613.2310 | 0.5027 | 0.0005 | 0.0194 |
| ENSBTAG00000016328 | *SFPQ* | 6228.4676 | 0.5112 | 0.0003 | 0.0145 |
| ENSBTAG00000027317 | *RNF114* | 4618.7482 | 0.5120 | 0.0001 | 0.0056 |
| ENSBTAG00000008863 | *TMEM87A* | 4773.2518 | 0.5122 | 0.0001 | 0.0088 |
| ENSBTAG00000019951 | *PCYOX1L* | 494.2812 | 0.5133 | 0.0004 | 0.0187 |
| ENSBTAG00000021808 | *CACNB3* | 1704.6484 | 0.5162 | 0.0011 | 0.0350 |
| ENSBTAG00000021482 | *APH1A* | 2659.7675 | 0.5176 | 0.0001 | 0.0052 |
| ENSBTAG00000013270 | *TTC19* | 1198.6457 | 0.5194 | 0.0014 | 0.0404 |
| ENSBTAG00000010595 | *WDR92* | 908.1866 | 0.5209 | 0.0005 | 0.0203 |
| ENSBTAG00000003840 | *GUCY1B1* | 3235.1863 | 0.5210 | 0.0004 | 0.0187 |
| ENSBTAG00000008913 | *TMEM98* | 2015.3488 | 0.5362 | 0.0018 | 0.0471 |
| ENSBTAG00000000495 | *HAVCR2* | 827.2449 | 0.5419 | 0.0008 | 0.0268 |
| ENSBTAG00000031464 |  | 2087.6212 | 0.5434 | 0.0018 | 0.0479 |
| ENSBTAG00000016874 | *DNAJB1* | 4442.9694 | 0.5617 | 0.0001 | 0.0077 |
| ENSBTAG00000018555 | *SFXN5* | 355.9909 | 0.5635 | 0.0006 | 0.0237 |
| ENSBTAG00000003397 | *CTBP2* | 4482.9007 | 0.5657 | 0.0003 | 0.0131 |
| ENSBTAG00000007271 | *TCTN2* | 1049.9218 | 0.5721 | 0.0013 | 0.0397 |
| ENSBTAG00000015109 | *TOB2* | 5086.9733 | 0.5735 | 0.0000 | 0.0034 |
| ENSBTAG00000002743 | *OSGEP* | 865.2625 | 0.5742 | 0.0004 | 0.0187 |
| ENSBTAG00000018588 | *TMBIM6* | 44965.7908 | 0.5743 | 0.0004 | 0.0179 |
| ENSBTAG00000006886 | *FAM117A* | 1472.2738 | 0.5770 | 0.0003 | 0.0150 |
| ENSBTAG00000014373 | *CETN3* | 1507.3304 | 0.5808 | 0.0011 | 0.0337 |
| ENSBTAG00000000182 | *SPINT2* | 17670.2402 | 0.5847 | 0.0000 | 0.0018 |
| ENSBTAG00000022382 | *TMEM218* | 889.5813 | 0.5857 | 0.0013 | 0.0377 |
| ENSBTAG00000026993 | *CGI-119* | 4275.9924 | 0.5860 | 0.0015 | 0.0420 |
| ENSBTAG00000033464 | *SPA17* | 3396.1662 | 0.5883 | 0.0001 | 0.0062 |
| ENSBTAG00000003882 | *EMC3* | 2016.1011 | 0.5891 | 0.0003 | 0.0143 |
| ENSBTAG00000019836 | *GSKIP* | 3721.6299 | 0.5901 | 0.0004 | 0.0179 |
| ENSBTAG00000034396 | *PIGW* | 892.9394 | 0.5937 | 0.0001 | 0.0046 |
| ENSBTAG00000007700 | *PHYH* | 2130.8323 | 0.5939 | 0.0004 | 0.0190 |
| ENSBTAG00000016969 | *ORC3* | 1821.3708 | 0.5990 | 0.0008 | 0.0279 |
| ENSBTAG00000011911 | *NCOA7* | 1299.2751 | 0.5995 | 0.0006 | 0.0216 |
| ENSBTAG00000006270 | *HSP90AA1* | 54140.4973 | 0.5997 | 0.0000 | 0.0005 |
| ENSBTAG00000010359 | *THG1L* | 1412.1983 | 0.6008 | 0.0018 | 0.0480 |
| ENSBTAG00000013726 | *RNPEP* | 2436.9280 | 0.6017 | 0.0020 | 0.0498 |
| ENSBTAG00000000655 | *MIPOL1* | 306.3993 | 0.6048 | 0.0002 | 0.0098 |
| ENSBTAG00000013010 | *CCDC126* | 703.7956 | 0.6076 | 0.0012 | 0.0352 |
| ENSBTAG00000018868 | *METTL9* | 10379.6902 | 0.6077 | 0.0004 | 0.0188 |
| ENSBTAG00000027803 |  | 3896.8749 | 0.6078 | 0.0002 | 0.0106 |
| ENSBTAG00000013579 | *TMEM66* | 8624.7814 | 0.6079 | 0.0007 | 0.0262 |
| ENSBTAG00000010756 | *ST7* | 1045.0923 | 0.6105 | 0.0004 | 0.0185 |
| ENSBTAG00000048314 |  | 2688.9102 | 0.6109 | 0.0008 | 0.0262 |
| ENSBTAG00000007480 | *CIRBP* | 9250.8084 | 0.6135 | 0.0010 | 0.0312 |
| ENSBTAG00000015955 | *ERGIC1* | 9338.6033 | 0.6156 | 0.0001 | 0.0063 |
| ENSBTAG00000016164 | *LMBRD1* | 2699.6713 | 0.6181 | 0.0005 | 0.0194 |
| ENSBTAG00000008072 | *FUSIP1* | 2062.5290 | 0.6261 | 0.0004 | 0.0160 |
| ENSBTAG00000032862 | *DYDC2* | 1522.0290 | 0.6307 | 0.0017 | 0.0456 |
| ENSBTAG00000003109 | *ITM2B* | 45715.1325 | 0.6343 | 0.0008 | 0.0281 |
| ENSBTAG00000039593 | *FAM98C* | 232.1464 | 0.6344 | 0.0015 | 0.0416 |
| ENSBTAG00000013162 | *HSPA8* | 65001.9159 | 0.6354 | 0.0002 | 0.0107 |
| ENSBTAG00000017564 | *CGN* | 3514.3679 | 0.6360 | 0.0010 | 0.0314 |
| ENSBTAG00000014354 | *FXYD6* | 3921.5462 | 0.6367 | 0.0014 | 0.0408 |
| ENSBTAG00000020169 | *CEPT1* | 2320.1558 | 0.6431 | 0.0000 | 0.0006 |
| ENSBTAG00000010191 | *PAK1* | 1851.9700 | 0.6434 | 0.0006 | 0.0234 |
| ENSBTAG00000009415 | *NUFIP1* | 409.1283 | 0.6463 | 0.0002 | 0.0107 |
| ENSBTAG00000010156 | *TPT1* | 1715.2623 | 0.6517 | 0.0010 | 0.0327 |
| ENSBTAG00000011765 | *GABARAPL1* | 6198.1936 | 0.6527 | 0.0001 | 0.0043 |
| ENSBTAG00000010007 | *MAPK13* | 737.1941 | 0.6545 | 0.0015 | 0.0420 |
| ENSBTAG00000047051 |  | 8263.9015 | 0.6594 | 0.0016 | 0.0429 |
| ENSBTAG00000010559 | *DHRS7B* | 916.8598 | 0.6616 | 0.0001 | 0.0079 |
| ENSBTAG00000008028 | *CHN1* | 1217.4682 | 0.6646 | 0.0011 | 0.0341 |
| ENSBTAG00000003027 | *EMX2* | 3955.5672 | 0.6676 | 0.0007 | 0.0258 |
| ENSBTAG00000000365 | *TDP2* | 1125.4545 | 0.6676 | 0.0004 | 0.0174 |
| ENSBTAG00000008931 | *CREG1* | 2938.1821 | 0.6706 | 0.0001 | 0.0079 |
| ENSBTAG00000013885 | *ADSS* | 3666.1652 | 0.6710 | 0.0001 | 0.0050 |
| ENSBTAG00000016349 | *TEAD2* | 1785.3389 | 0.6717 | 0.0002 | 0.0095 |
| ENSBTAG00000002651 | *PKP2* | 3371.3401 | 0.6735 | 0.0018 | 0.0465 |
| ENSBTAG00000019110 | *PNRC1* | 3300.0736 | 0.6850 | 0.0000 | 0.0001 |
| ENSBTAG00000012451 | *HLA-DMB* | 1050.6045 | 0.6880 | 0.0019 | 0.0487 |
| ENSBTAG00000013136 | *EFCAB11* | 446.4855 | 0.6924 | 0.0008 | 0.0271 |
| ENSBTAG00000015202 | *ATP5S* | 591.9338 | 0.6992 | 0.0002 | 0.0105 |
| ENSBTAG00000008952 | *RAP1GAP* | 646.0954 | 0.6997 | 0.0001 | 0.0053 |
| ENSBTAG00000014750 | *EPB41L4A* | 874.6856 | 0.7013 | 0.0013 | 0.0387 |
| ENSBTAG00000000240 | *AKAP7* | 351.2231 | 0.7019 | 0.0007 | 0.0257 |
| ENSBTAG00000013926 | *FCGRT* | 5339.5229 | 0.7024 | 0.0002 | 0.0118 |
| ENSBTAG00000005761 | *DEDD2* | 865.6068 | 0.7219 | 0.0000 | 0.0011 |
| ENSBTAG00000004855 | *PRDX6* | 17534.4320 | 0.7225 | 0.0000 | 0.0000 |
| ENSBTAG00000010548 | *LRRC6* | 820.5675 | 0.7230 | 0.0002 | 0.0088 |
| ENSBTAG00000008331 | *TMEM54* | 155.4099 | 0.7269 | 0.0019 | 0.0487 |
| ENSBTAG00000005635 | *TTC39C* | 1019.5714 | 0.7310 | 0.0000 | 0.0014 |
| ENSBTAG00000013414 | *BLVRA* | 1560.6650 | 0.7338 | 0.0003 | 0.0147 |
| ENSBTAG00000019448 | *CLDN7* | 4497.1358 | 0.7421 | 0.0002 | 0.0095 |
| ENSBTAG00000006287 | *NEDD9* | 3736.9159 | 0.7431 | 0.0000 | 0.0029 |
| ENSBTAG00000009191 | *TUSC3* | 2455.4904 | 0.7477 | 0.0000 | 0.0000 |
| ENSBTAG00000045794 | *PL-5283* | 6482.9130 | 0.7487 | 0.0000 | 0.0001 |
| ENSBTAG00000048229 | *TPT1* | 94432.2770 | 0.7488 | 0.0000 | 0.0000 |
| ENSBTAG00000020884 | *CASP13* | 1271.7370 | 0.7492 | 0.0000 | 0.0000 |
| ENSBTAG00000010368 | *TPST2* | 2384.1078 | 0.7501 | 0.0006 | 0.0237 |
| ENSBTAG00000048125 | *ENTPD2* | 922.8463 | 0.7543 | 0.0002 | 0.0090 |
| ENSBTAG00000004597 | *PIM2* | 906.8101 | 0.7591 | 0.0007 | 0.0243 |
| ENSBTAG00000019246 | *SC5DL* | 5182.3539 | 0.7697 | 0.0001 | 0.0065 |
| ENSBTAG00000006441 | *ATP5F1* | 6156.4041 | 0.7760 | 0.0000 | 0.0033 |
| ENSBTAG00000019017 | *IFITM2* | 257.5971 | 0.7772 | 0.0017 | 0.0451 |
| ENSBTAG00000006474 | *EPCAM* | 14296.4275 | 0.7774 | 0.0004 | 0.0175 |
| ENSBTAG00000021378 | *S100A13* | 1081.9740 | 0.7775 | 0.0000 | 0.0002 |
| ENSBTAG00000016615 | *ABHD6* | 484.9987 | 0.7867 | 0.0010 | 0.0314 |
| ENSBTAG00000020600 | *RHOD* | 255.2307 | 0.7898 | 0.0013 | 0.0385 |
| ENSBTAG00000020559 | *STMND1* | 545.4000 | 0.7930 | 0.0014 | 0.0404 |
| ENSBTAG00000005675 | *SLC44A4* | 2076.4033 | 0.7960 | 0.0006 | 0.0224 |
| ENSBTAG00000005012 | *HSPH1* | 9505.3327 | 0.7983 | 0.0016 | 0.0432 |
| ENSBTAG00000036349 | *CCDC160* | 255.5375 | 0.7995 | 0.0002 | 0.0098 |
| ENSBTAG00000009494 | *CBLC* | 452.4875 | 0.8004 | 0.0004 | 0.0185 |
| ENSBTAG00000006561 | *RASGEF1B* | 1228.2018 | 0.8047 | 0.0000 | 0.0033 |
| ENSBTAG00000036127 | *AS3MT* | 840.8875 | 0.8087 | 0.0003 | 0.0159 |
| ENSBTAG00000002670 | *C28H10ORF10* | 115.2259 | 0.8101 | 0.0020 | 0.0498 |
| ENSBTAG00000018349 | *IFI30* | 734.4304 | 0.8103 | 0.0009 | 0.0301 |
| ENSBTAG00000001889 | *C3orf14* | 197.4641 | 0.8143 | 0.0002 | 0.0127 |
| ENSBTAG00000018663 | *GPRC5C* | 1464.8924 | 0.8185 | 0.0000 | 0.0012 |
| ENSBTAG00000008541 | *MGST1* | 4420.9863 | 0.8214 | 0.0007 | 0.0245 |
| ENSBTAG00000048010 | *NUDT14* | 727.3259 | 0.8218 | 0.0017 | 0.0451 |
| ENSBTAG00000021899 | *PDE9A* | 398.4837 | 0.8219 | 0.0000 | 0.0005 |
| ENSBTAG00000021614 | *C20ORF13* | 1171.1133 | 0.8240 | 0.0000 | 0.0001 |
| ENSBTAG00000017752 | *DYNLRB2* | 1661.3068 | 0.8253 | 0.0006 | 0.0230 |
| ENSBTAG00000006928 | *OAT* | 5699.5771 | 0.8288 | 0.0010 | 0.0314 |
| ENSBTAG00000005217 |  | 108.5337 | 0.8322 | 0.0005 | 0.0215 |
| ENSBTAG00000039335 | *ARRDC2* | 842.7947 | 0.8322 | 0.0001 | 0.0048 |
| ENSBTAG00000018706 | *PCTP* | 703.9001 | 0.8331 | 0.0002 | 0.0101 |
| ENSBTAG00000003539 | *ROPN1L* | 1798.8726 | 0.8368 | 0.0004 | 0.0185 |
| ENSBTAG00000011600 | *MAP3K8* | 304.2190 | 0.8369 | 0.0018 | 0.0471 |
| ENSBTAG00000015426 | *PDLIM4* | 2581.6409 | 0.8393 | 0.0003 | 0.0132 |
| ENSBTAG00000003440 | *FRRS1* | 667.1296 | 0.8499 | 0.0002 | 0.0096 |
| ENSBTAG00000009289 | *MS4A8B* | 7601.6576 | 0.8500 | 0.0010 | 0.0319 |
| ENSBTAG00000047229 | *CRIP1* | 4273.0535 | 0.8545 | 0.0001 | 0.0062 |
| ENSBTAG00000021779 | *MGST2* | 2263.6058 | 0.8601 | 0.0000 | 0.0000 |
| ENSBTAG00000000489 | *WDR54* | 650.5325 | 0.8649 | 0.0000 | 0.0004 |
| ENSBTAG00000009839 | *GSTK1* | 936.5198 | 0.8734 | 0.0000 | 0.0000 |
| ENSBTAG00000025778 | *EMC9* | 194.0840 | 0.8785 | 0.0006 | 0.0221 |
| ENSBTAG00000016926 | *C13H20ORF85* | 847.9479 | 0.8785 | 0.0000 | 0.0018 |
| ENSBTAG00000014191 | *QSOX1* | 6908.7678 | 0.8829 | 0.0011 | 0.0337 |
| ENSBTAG00000007578 | *KIAA1598* | 1966.2377 | 0.8939 | 0.0001 | 0.0082 |
| ENSBTAG00000007534 | *GGH* | 340.4083 | 0.9035 | 0.0002 | 0.0107 |
| ENSBTAG00000019822 | *TPPP3* | 14026.3218 | 0.9080 | 0.0000 | 0.0015 |
| ENSBTAG00000016265 | *DNAJA1* | 960.6004 | 0.9093 | 0.0012 | 0.0368 |
| ENSBTAG00000002117 | *KIF18A* | 44.5179 | 0.9125 | 0.0014 | 0.0404 |
| ENSBTAG00000034586 |  | 943.6283 | 0.9126 | 0.0001 | 0.0058 |
| ENSBTAG00000017051 | *ACP6* | 847.2684 | 0.9130 | 0.0006 | 0.0237 |
| ENSBTAG00000019015 | *IFITM3* | 3421.5023 | 0.9169 | 0.0000 | 0.0001 |
| ENSBTAG00000014685 | *HPRT1* | 2491.4740 | 0.9227 | 0.0000 | 0.0014 |
| ENSBTAG00000014204 | *S100A16* | 3448.4389 | 0.9230 | 0.0001 | 0.0079 |
| ENSBTAG00000007895 | *SLC20A1* | 3631.2274 | 0.9264 | 0.0010 | 0.0327 |
| ENSBTAG00000000559 | *PLTP* | 3955.3613 | 0.9288 | 0.0003 | 0.0132 |
| ENSBTAG00000016092 | *SPATS2L* | 3542.7389 | 0.9293 | 0.0001 | 0.0054 |
| ENSBTAG00000000569 | *HES1* | 946.7079 | 0.9336 | 0.0019 | 0.0488 |
| ENSBTAG00000016683 | *BIKBA* | 4353.8359 | 0.9492 | 0.0000 | 0.0001 |
| ENSBTAG00000000140 | *EPHX1* | 3188.4590 | 0.9503 | 0.0000 | 0.0002 |
| ENSBTAG00000015908 | *MBOAT7* | 993.7549 | 0.9540 | 0.0003 | 0.0159 |
| ENSBTAG00000005018 | *NFAM1* | 153.2377 | 0.9553 | 0.0005 | 0.0215 |
| ENSBTAG00000011377 | *KCNMB3* | 236.3980 | 0.9568 | 0.0019 | 0.0487 |
| ENSBTAG00000031750 | *PLAC8* | 502.0872 | 0.9593 | 0.0000 | 0.0001 |
| ENSBTAG00000018417 | *PSMF1* | 2288.0148 | 0.9632 | 0.0001 | 0.0050 |
| ENSBTAG00000012854 | *GSDMB* | 108.2667 | 0.9676 | 0.0007 | 0.0243 |
| ENSBTAG00000011382 | *SLC9A3R1* | 5450.8450 | 0.9677 | 0.0005 | 0.0207 |
| ENSBTAG00000046971 |  | 2803.9650 | 0.9712 | 0.0007 | 0.0241 |
| ENSBTAG00000002069 | *BOLA* | 7068.2390 | 0.9832 | 0.0001 | 0.0053 |
| ENSBTAG00000008900 | *CELA1* | 56.4337 | 0.9869 | 0.0010 | 0.0312 |
| ENSBTAG00000047379 |  | 82.3461 | 0.9890 | 0.0001 | 0.0075 |
| ENSBTAG00000007502 | *CKMT1* | 1293.4579 | 0.9916 | 0.0001 | 0.0055 |
| ENSBTAG00000004004 | *CSDC2* | 501.4896 | 0.9926 | 0.0000 | 0.0023 |
| ENSBTAG00000014830 | *PPP1R32* | 1485.5518 | 0.9935 | 0.0003 | 0.0144 |
| ENSBTAG00000000641 | *SKAP1* | 1114.8397 | 0.9943 | 0.0000 | 0.0026 |
| ENSBTAG00000002508 | *GNG3* | 112.8344 | 0.9969 | 0.0000 | 0.0023 |
| ENSBTAG00000001710 | *CMTM8* | 809.3521 | 0.9979 | 0.0000 | 0.0031 |
| ENSBTAG00000020062 | *TMC4* | 3573.2683 | 1.0064 | 0.0000 | 0.0040 |
| ENSBTAG00000016041 | *B3GNT8* | 799.9294 | 1.0065 | 0.0000 | 0.0003 |
| ENSBTAG00000007436 | *GGT6* | 185.5903 | 1.0102 | 0.0001 | 0.0050 |
| ENSBTAG00000001244 | *PLAT* | 2586.7793 | 1.0156 | 0.0000 | 0.0003 |
| ENSBTAG00000005709 | *ABHD3* | 472.6446 | 1.0197 | 0.0015 | 0.0423 |
| ENSBTAG00000004976 | *CDCA7L* | 5294.3069 | 1.0208 | 0.0011 | 0.0344 |
| ENSBTAG00000006947 | *ROPN1* | 1948.9914 | 1.0258 | 0.0001 | 0.0046 |
| ENSBTAG00000023659 | *MT2* | 766.9749 | 1.0270 | 0.0001 | 0.0063 |
| ENSBTAG00000013026 | *SNTN* | 288.6225 | 1.0306 | 0.0008 | 0.0268 |
| ENSBTAG00000005574 | *CLU* | 26595.1427 | 1.0339 | 0.0000 | 0.0001 |
| ENSBTAG00000025441 | *HSPA1A* | 15968.4859 | 1.0356 | 0.0013 | 0.0388 |
| ENSBTAG00000021582 | *HCAP-G* | 82.4196 | 1.0377 | 0.0006 | 0.0234 |
| ENSBTAG00000046014 | *GPR146* | 207.6648 | 1.0395 | 0.0016 | 0.0440 |
| ENSBTAG00000002522 | *SYT5* | 2082.3001 | 1.0462 | 0.0001 | 0.0050 |
| ENSBTAG00000000046 | *SURF2* | 459.2607 | 1.0554 | 0.0003 | 0.0157 |
| ENSBTAG00000003692 | *UCP2* | 3426.5302 | 1.0593 | 0.0000 | 0.0000 |
| ENSBTAG00000000668 | *SLC22A5* | 1433.4318 | 1.0627 | 0.0006 | 0.0239 |
| ENSBTAG00000008538 | *DNAI1* | 884.1469 | 1.0659 | 0.0003 | 0.0139 |
| ENSBTAG00000031246 | *CCNI2* | 141.3677 | 1.0671 | 0.0000 | 0.0011 |
| ENSBTAG00000012980 | *RRAGB* | 1064.5975 | 1.0690 | 0.0000 | 0.0000 |
| ENSBTAG00000001694 | *TYRO3* | 1373.4000 | 1.0726 | 0.0018 | 0.0474 |
| ENSBTAG00000004296 | *MORN5* | 539.7186 | 1.0795 | 0.0000 | 0.0000 |
| ENSBTAG00000019722 | *GUCY2C* | 70.6892 | 1.0873 | 0.0002 | 0.0124 |
| ENSBTAG00000009384 | *AGTRAP* | 1472.2809 | 1.0933 | 0.0000 | 0.0000 |
| ENSBTAG00000001163 | *H2AFY2* | 1107.6521 | 1.0967 | 0.0005 | 0.0194 |
| ENSBTAG00000014972 | *PTGER4* | 1509.0215 | 1.0977 | 0.0000 | 0.0000 |
| ENSBTAG00000039090 | *MAGEH1* | 3487.6814 | 1.1001 | 0.0000 | 0.0001 |
| ENSBTAG00000018232 | *STOML3* | 270.0906 | 1.1048 | 0.0014 | 0.0401 |
| ENSBTAG00000010682 | *DDR1* | 9912.1699 | 1.1126 | 0.0017 | 0.0451 |
| ENSBTAG00000018216 | *SKA1* | 210.2769 | 1.1157 | 0.0000 | 0.0003 |
| ENSBTAG00000004136 | *NFE2L3* | 1933.0836 | 1.1166 | 0.0000 | 0.0001 |
| ENSBTAG00000014670 | *BEX5* | 1031.5713 | 1.1228 | 0.0000 | 0.0027 |
| ENSBTAG00000005638 | *CABYR* | 98.1355 | 1.1246 | 0.0004 | 0.0169 |
| ENSBTAG00000026236 | *AZGP1* | 15505.7731 | 1.1251 | 0.0002 | 0.0099 |
| ENSBTAG00000019927 | *CYB5R1* | 1749.3826 | 1.1344 | 0.0000 | 0.0000 |
| ENSBTAG00000047902 |  | 61.8927 | 1.1355 | 0.0017 | 0.0452 |
| ENSBTAG00000020605 | *SMTNL2* | 1181.7788 | 1.1407 | 0.0000 | 0.0000 |
| ENSBTAG00000046862 | *MARCKSL1* | 1703.3994 | 1.1442 | 0.0005 | 0.0214 |
| ENSBTAG00000014611 | *SORL1* | 7375.2224 | 1.1448 | 0.0013 | 0.0388 |
| ENSBTAG00000016165 | *KRT7* | 2829.0361 | 1.1571 | 0.0015 | 0.0420 |
| ENSBTAG00000015094 | *VNN1* | 96.0309 | 1.1639 | 0.0014 | 0.0406 |
| ENSBTAG00000018206 | *NIPAL2* | 1917.7184 | 1.1639 | 0.0001 | 0.0083 |
| ENSBTAG00000019552 | *PGRMC1* | 10397.0257 | 1.1695 | 0.0000 | 0.0007 |
| ENSBTAG00000017040 | *LY6E* | 29288.3862 | 1.1745 | 0.0000 | 0.0035 |
| ENSBTAG00000016048 | *CPT1B* | 1493.0364 | 1.1850 | 0.0007 | 0.0262 |
| ENSBTAG00000027181 | *LAMA3* | 3491.3837 | 1.1934 | 0.0012 | 0.0375 |
| ENSBTAG00000033565 |  | 795.6130 | 1.2028 | 0.0007 | 0.0257 |
| ENSBTAG00000046548 | *SIAT7B* | 948.9424 | 1.2179 | 0.0001 | 0.0066 |
| ENSBTAG00000002863 | *ACAA2* | 4492.7851 | 1.2226 | 0.0000 | 0.0025 |
| ENSBTAG00000005699 | *SCML4* | 20.8000 | 1.2232 | 0.0011 | 0.0337 |
| ENSBTAG00000011582 | *SERINC2* | 1615.6839 | 1.2306 | 0.0000 | 0.0000 |
| ENSBTAG000000312097 | *bta-mir-2457* | 119.1509 | 1.2322 | 0.0000 | 0.0000 |
| ENSBTAG00000001331 | *FAM194A* | 151.5604 | 1.2379 | 0.0016 | 0.0429 |
| ENSBTAG00000018245 | *SLC1A3* | 115.0846 | 1.2412 | 0.0018 | 0.0468 |
| ENSBTAG00000015685 | *KIF12* | 89.9016 | 1.2426 | 0.0014 | 0.0408 |
| ENSBTAG00000014760 | *FOXE1* | 36.9345 | 1.2508 | 0.0014 | 0.0405 |
| ENSBTAG00000013488 | *PPP1R14D* | 78.0111 | 1.2539 | 0.0000 | 0.0004 |
| ENSBTAG00000014534 | *EEF1A1* | 18363.1936 | 1.2552 | 0.0006 | 0.0237 |
| ENSBTAG00000046266 | *TNFSF9* | 56.2606 | 1.2571 | 0.0005 | 0.0207 |
| ENSBTAG00000008686 | *SLC46A3* | 824.5629 | 1.2623 | 0.0005 | 0.0204 |
| ENSBTAG00000003791 | *LPAR3* | 2515.2786 | 1.2724 | 0.0006 | 0.0234 |
| ENSBTAG00000016741 | *TOX2* | 89.4176 | 1.2749 | 0.0016 | 0.0432 |
| ENSBTAG00000047268 | *WT1* | 3563.9203 | 1.2784 | 0.0019 | 0.0488 |
| ENSBTAG00000003568 | *CLDN10* | 2646.7918 | 1.2788 | 0.0000 | 0.0000 |
| ENSBTAG00000014863 | *GPC* | 1038.7944 | 1.2876 | 0.0001 | 0.0062 |
| ENSBTAG00000015313 | *CEACAM19* | 32.1830 | 1.2890 | 0.0001 | 0.0088 |
| ENSBTAG00000047482 | *PRRT4* | 45.8048 | 1.2895 | 0.0015 | 0.0428 |
| ENSBTAG00000011423 | *SERPINI2* | 77.1757 | 1.2904 | 0.0009 | 0.0289 |
| ENSBTAG00000007353 | *DLX4* | 46.4679 | 1.2938 | 0.0003 | 0.0138 |
| ENSBTAG00000000188 | *HS3ST5* | 86.7561 | 1.2952 | 0.0001 | 0.0083 |
| ENSBTAG00000006868 | *OTUD7A* | 38.9157 | 1.3011 | 0.0016 | 0.0437 |
| ENSBTAG00000030932 | *IFI44L* | 742.8152 | 1.3034 | 0.0013 | 0.0391 |
| ENSBTAG00000030921 | *FAM3B* | 519.6148 | 1.3066 | 0.0014 | 0.0404 |
| ENSBTAG00000007958 | *TNS4* | 89.0911 | 1.3156 | 0.0015 | 0.0419 |
| ENSBTAG00000017242 | *FADS6* | 56.2379 | 1.3160 | 0.0003 | 0.0150 |
| ENSBTAG00000018240 | *CYP2S1* | 178.0851 | 1.3162 | 0.0009 | 0.0291 |
| ENSBTAG00000005150 | *HMGB3* | 157.5078 | 1.3197 | 0.0000 | 0.0000 |
| ENSBTAG00000017715 | *HOMER2* | 617.3526 | 1.3202 | 0.0001 | 0.0062 |
| ENSBTAG00000014721 | *MUC4* | 248.7328 | 1.3277 | 0.0004 | 0.0180 |
| ENSBTAG00000016210 | *LYPD2* | 15.2193 | 1.3500 | 0.0006 | 0.0237 |
| ENSBTAG00000006446 |  | 121.2263 | 1.3512 | 0.0012 | 0.0359 |
| ENSBTAG00000021887 | *DPYS* | 259.9287 | 1.3522 | 0.0009 | 0.0299 |
| ENSBTAG00000047650 | *NXPH4* | 7.2818 | 1.3598 | 0.0011 | 0.0341 |
| ENSBTAG00000037555 | *FAM183A* | 2189.9031 | 1.3625 | 0.0000 | 0.0001 |
| ENSBTAG00000003152 | *IFI27* | 1652.2709 | 1.3720 | 0.0007 | 0.0262 |
| ENSBTAG00000007554 | *IFI6* | 8737.6893 | 1.3738 | 0.0010 | 0.0312 |
| ENSBTAG00000021390 | *TMEM89* | 11.6236 | 1.3768 | 0.0009 | 0.0290 |
| ENSBTAG00000007581 | *ADORA2B* | 668.8230 | 1.3850 | 0.0000 | 0.0013 |
| ENSBTAG00000001595 | *MT1E* | 109.1510 | 1.3879 | 0.0001 | 0.0071 |
| ENSBTAG00000012234 | *RHBG* | 13.1763 | 1.3909 | 0.0005 | 0.0196 |
| ENSBTAG00000006081 | *PPM1J* | 111.8110 | 1.3912 | 0.0008 | 0.0268 |
| ENSBTAG00000012215 | *CPNE7* | 60.3918 | 1.3982 | 0.0004 | 0.0175 |
| ENSBTAG00000010316 | *ICAM5* | 46.1072 | 1.3987 | 0.0008 | 0.0272 |
| ENSBTAG00000013406 | *CSRP2* | 1657.1469 | 1.4047 | 0.0000 | 0.0000 |
| ENSBTAG00000038974 | *STMN3* | 146.4192 | 1.4052 | 0.0007 | 0.0262 |
| ENSBTAG00000002065 | *TFAP2C* | 117.3205 | 1.4094 | 0.0007 | 0.0244 |
| ENSBTAG00000021993 | *SPDEF* | 5750.7737 | 1.4165 | 0.0000 | 0.0007 |
| ENSBTAG00000015562 | *PLBD1* | 2011.6593 | 1.4371 | 0.0002 | 0.0093 |
| ENSBTAG00000046017 | *POPDC3* | 658.9998 | 1.4401 | 0.0000 | 0.0003 |
| ENSBTAG00000004594 | *MMP7* | 39.2508 | 1.4532 | 0.0005 | 0.0197 |
| ENSBTAG00000031711 |  | 324.9679 | 1.4564 | 0.0000 | 0.0000 |
| ENSBTAG00000019277 | *KCNH3* | 985.1293 | 1.4778 | 0.0003 | 0.0137 |
| ENSBTAG00000002181 | *KHDRBS3* | 500.5878 | 1.4780 | 0.0001 | 0.0048 |
| ENSBTAG00000007148 | *F2* | 454.0236 | 1.4875 | 0.0001 | 0.0060 |
| ENSBTAG00000025101 | *ASGR2* | 97.1014 | 1.4969 | 0.0002 | 0.0120 |
| ENSBTAG00000038706 | *MT1E* | 49.6815 | 1.5106 | 0.0000 | 0.0027 |
| ENSBTAG00000001388 | *NMB* | 3425.7936 | 1.5108 | 0.0000 | 0.0000 |
| ENSBTAG00000001255 | *CRYBB3* | 21.1571 | 1.5120 | 0.0003 | 0.0140 |
| ENSBTAG00000031631 | *FAM132A* | 42.0764 | 1.5444 | 0.0002 | 0.0109 |
| ENSBTAG00000013124 | *LRTM1* | 25.7486 | 1.5492 | 0.0001 | 0.0074 |
| ENSBTAG00000018989 | *GRM3* | 34.3159 | 1.5505 | 0.0002 | 0.0098 |
| ENSBTAG00000012742 | *SLC22A18* | 488.8165 | 1.5525 | 0.0000 | 0.0000 |
| ENSBTAG00000001851 |  | 41.2830 | 1.5627 | 0.0002 | 0.0093 |
| ENSBTAG00000009837 | *ANKS4B* | 85.8202 | 1.5642 | 0.0001 | 0.0078 |
| ENSBTAG00000023784 | *TMEM88B* | 249.0948 | 1.5671 | 0.0001 | 0.0083 |
| ENSBTAG00000001558 | *PATL2* | 38.4956 | 1.5684 | 0.0002 | 0.0093 |
| ENSBTAG00000000533 | *ZNF684* | 63.4107 | 1.5841 | 0.0000 | 0.0010 |
| ENSBTAG00000014102 | *WDR77* | 3868.0216 | 1.5938 | 0.0001 | 0.0054 |
| ENSBTAG00000002075 | *MME* | 3266.7911 | 1.6132 | 0.0001 | 0.0041 |
| ENSBTAG00000006280 | *RBFOX3* | 119.7817 | 1.6585 | 0.0000 | 0.0036 |
| ENSBTAG00000001785 | *TGM3* | 48.1331 | 1.6793 | 0.0000 | 0.0019 |
| ENSBTAG00000004777 | *S100B* | 13710.4501 | 1.7360 | 0.0000 | 0.0009 |
| ENSBTAG00000033220 | *C3H1orf189* | 849.4682 | 1.7437 | 0.0000 | 0.0000 |
| ENSBTAG00000005261 | *FCGBP* | 3301.8386 | 1.7684 | 0.0000 | 0.0000 |
| ENSBTAG00000018703 | *OSTN* | 715.4376 | 1.7712 | 0.0000 | 0.0000 |
| ENSBTAG00000000177 | *MSLN* | 6923.0864 | 1.7904 | 0.0000 | 0.0017 |
| ENSBTAG00000047225 | *BCL2L14* | 76.6531 | 1.8303 | 0.0000 | 0.0013 |
| ENSBTAG00000025718 | *HMGCLL1* | 17.3766 | 1.8404 | 0.0000 | 0.0009 |
| ENSBTAG00000005996 | *LRRC18* | 100.4409 | 1.8650 | 0.0000 | 0.0002 |
| ENSBTAG00000012185 | *CLEC4E* | 63.3652 | 1.8690 | 0.0000 | 0.0001 |
| ENSBTAG00000015345 | *BNIPL* | 374.4034 | 1.9795 | 0.0000 | 0.0003 |
| ENSBTAG00000031704 | *MGC133804* | 43.2403 | 2.3016 | 0.0000 | 0.0000 |
