# Supplementary material for: Size of the Ovulatory Follicle Dictates Spatial Differences in the Oviductal Transcriptome in Cattle
Source: PLoS One. 2015 Dec 23;10(12):e0145321. doi: 10.1371/journal.pone.0145321 (PMC4689418; doi:10.1371/journal.pone.0145321)
Supplement: S6 Table — Validation of RNAseq gene expression data by qPCR. qPCR data was analyzed using the same RNAseq animals (n = 6) and using 7 animals for each group (n = 14). (DOCX) [file pone.0145321.s008.docx]

**S6 Table. Log2 Fold change and *P value* of isthmus gene expression in LF/LCL and SF/SCL animals.** Validation of RNAseq gene expression data by qPCR. qPCR data was analyzed using the same RNAseq animals (n=6) and using 7 animals for each group (n=14).

| **Ensembl Id** | **Gene Symbol** | **RNAseq (n=6)** | | **qPCR (n=6)** | | **qPCR (n=14)** | |
| --- | --- | --- | --- | --- | --- | --- | --- |
|  |  | **log2 Fold Change** | ***P* value** | **log2 Fold Change** | ***P* value** | **log2 Fold Change** | ***P* value** |
| ENSBTAG00000011034 | ANGPT2 | -0.374 | 0.627 | -0.505 | 0.338 | -0.395 | 0.292 |
| ENSBTAG00000021811 | ANGPT4 | -0.398 | 0.709 | -0.619 | 0.332 | -0.107 | 0.444 |
| ENSBTAG00000003217 | CADM3 | 0.706 | NA | 0.208 | 0.447 | 0.0602 | 0.475 |
| ENSBTAG00000006161 | C-MET | -0.730 | ˂ 0.001 | -0.591 | 0.001 | -0.375 | 0.023 |
| ENSBTAG00000006367 | CTGF | 0.168 | 0.923 | 0.368 | 0.471 | 0.001 | 0.499 |
| ENSBTAG00000017135 | CTSS | 0.302 | 0.312 | 0.399 | 0.226 | 0.579 | 0.026 |
| ENSBTAG00000001060 | CXCR4 | -0.298 | 0.390 | -1.753 | 0.001 | -4.029 | ˂ 0.001 |
| ENSBTAG00000008096 | EDN1 | 0.132 | 0.925 | 0.186 | 0.385 | 1.075 | 0.012 |
| ENSBTAG00000007159 | ESR1 | 0.284 | NA | 0.414 | 0.168 | 0.038 | 0.448 |
| ENSBTAG00000004498 | ESR2 | -0.323 | NA | -1.190 | 0.205 | -0.416 | 0.249 |
| ENSBTAG00000005745 | HPSE | -1.017 | 0.183 | -0.689 | 0.246 | -1.901 | 0.224 |
| ENSBTAG00000025441 | HSPA1A | 1.035 | 0.038 | 0.319 | 0.405 | 0.847 | 0.290 |
| ENSBTAG00000005973 | OVGP1 | 0.597 | NA | 4.042 | 0.196 | 0.413 | 0.400 |
| ENSBTAG00000006065 | PCNA | 0.326 | 0.821 | 0.327 | 0.286 | 0.325 | 0.181 |
| ENSBTAG00000034827 | PDGF | -0.681 | ˂ 0.001 | -0.192 | 0.004 | -0.186 | 0.023 |
| ENSBTAG00000024648 | PGR | 0.347 | NA | 0.018 | 0.475 | 0.024 | 0.460 |
| ENSBTAG00000010843 | PGRMC2 | 0.136 | NA | 1.142 | 0.038 | 0.361 | 0.165 |
| ENSBTAG00000003454 | RGS20 | 0.368 | 0.833 | 2.356 | 0.147 | 0.404 | 0.068 |
| ENSBTAG00000005359 | TGFB2 | -0.539 | 0.181 | -0.723 | 0.099 | -0.654 | 0.018 |
| ENSBTAG00000012004 | TGFB3 | -0.727 | 0.081 | -0.770 | 0.088 | -0.667 | 0.065 |
| ENSBTAG00000018035 | TGFBR1 | -0.360 | 0.807 | -0.300 | 0.002 | -0.685 | 0.495 |
| ENSBTAG00000019832 | TGFBR2 | -0.276 | 0.341 | -0.246 | 0.521 | -0.674 | 0.814 |
| ENSBTAG00000021879 | VCL | -0.281 | 0.411 | -0.218 | 0.025 | -0.335 | 0.021 |
