# Supplementary material for: Size of the Ovulatory Follicle Dictates Spatial Differences in the Oviductal Transcriptome in Cattle
Source: PLoS One. 2015 Dec 23;10(12):e0145321. doi: 10.1371/journal.pone.0145321 (PMC4689418; doi:10.1371/journal.pone.0145321)
Supplement: S7 Table — Gene ontology analysis is performed with DAVID tools (http://david.abcc.ncifcrf.gov/tools.jsp). The enrichment p-values are corrected by Benjamini's methods. GO categories are presented according to their biological process, cellular component and molecular function. (DOCX) [file pone.0145321.s009.docx]

**S7 Table. Gene ontologies (GO category) of mRNA transcripts differentially expressed in day 4 Ampulla samples of the LF/LCL group.** Gene ontology analysis is performed with DAVID tools (http://david.abcc.ncifcrf.gov/tools.jsp). The enrichment p-values are corrected by Benjamini's methods. GO categories are presented according to their biological process, cellular component and molecular function.

| **Enriched process** | **Category** | **Term** | **Genes** | **Fold Enrichment** | **FDR** | ***P* Value** |
| --- | --- | --- | --- | --- | --- | --- |
| Immune cell activation | Biological Process | GO:0046635~positive regulation of alpha-beta T cell activation | *CD3E, IL2RG , SASH3* | 1.16 | 3.46 | 0.027 |
|  | Biological Process | GO:0050863~regulation of T cell activation | *BCL10, CD3E, CENPF , IL2RG , SASH3* | 4.59 | 3.05 | 0.023 |
|  | Biological Process | GO:0046634~regulation of alpha-beta T cell activation | *CD3E, IL2RG , SASH3* | 1.08 | 3.82 | 0.030 |
|  | Biological Process | GO:0051249~regulation of lymphocyte activation | *BCL10, CD3E, CENPF , IL2RG , SASH3* | 3.80 | 4.87 | 0.042 |
|  | Biological Process | GO:0050870~positive regulation of T cell activation | *BCL10, CD3E, IL2RG , SASH3* | 4.72 | 5.66 | 0.052 |
|  | Biological Process | GO:0002694~regulation of leukocyte activation | *BCL10, CD3E, CENPF , IL2RG , SASH3* | 3.40 | 6.12 | 0.058 |
| Vacuole and lysosome | Cellular Component | GO:0005773~vacuole | *ACP2, BCL10, GJA1, CTSS, PRDX6, C1orf85, FUCA1, CTSZ, GBA* | 4.72 | 0.70 | 0.000 |
|  | Cellular Component | GO:0005764~lysosome | *ACP2, BCL10, GJA1, CTSS, PRDX6, C1orf85, FUCA1, CTSZ, GBA* | 5.66 | 0.20 | 0.000 |
|  | Cellular Component | GO:0000323~lytic vacuole | *ACP2, BCL10, GJA1, CTSS, PRDX6, C1orf85, FUCA1, CTSZ, GBA* | 5.66 | 0.20 | 0.000 |
| Homeostasis | Biological Process | GO:0019725~cellular homeostasis | *MT3, GLRX5, PRDX6, NXN, NAB2, CAV1, SELT, PLN, SH3BGRL3, CD52* | 2.69 | 1.70 | 0.012 |
|  | Biological Process | GO:0042592~homeostatic process | *GLRX5, PRDX6, NAB2, SASH3, PLN, SELT, MT3, LCAT, NXN, CD7, CAV1, CD52, SH3BGRL3* | 2.13 | 2.53 | 0.018 |
|  | Biological Process | GO:0045454~cell redox homeostasis | *GLRX5, PRDX6, NXN, SELT, SH3BGRL3* | 5.45 | 1.83 | 0.013 |
|  | Molecular function | GO:0016667~oxidoreductase activity, acting on sulfur group of donors | *GLRX5, NXN, MSRB3 ,SH3BGRL3* | 1.02 | 8.44 | 0.007 |
| Cytoeskeleton and extracellular matrix | Biological Process | GO:0043297~apical junction assembly | *CTNNA1, VCL* | 5.78 | 4.21 | 0.034 |
|  | Cellular Component | GO:0044421~extracellular region part | *FGA, TWSG1,LOXL1, NTNG2, LAMA4 , OLFML2B, LCAT , MMP23B,LGALS1, CXCL10, SAA3, SPOCK1, SAA1, ANGPTL1, FIGF, LGALS3* | 2.12 | 8.65 | 0.007 |
|  | Cellular Component | GO:0031012~extracellular matrix | *OLFML2B, MMP23B,LGALS1, SPOCK1,LOXL1, NTNG2, LGALS3 , LAMA4* | 2.55 | 3.64 | 0.036 |
|  | Cellular Component | GO:0005615~extracellular space | *LCAT ,LGALS1, SAA3, CXCL10, FGA, TWSG1, SAA1,LOXL1, ANGPTL1 , FIGF* | 2.12 | 4.24 | 0.044 |
|  | Biological Process | GO:0030036~actin cytoskeleton organization | *PFN1, ARPC4, ACTB , ELMO1 , MYH7 ,* Uncharacterized protein (ENSBTAG00000009703), *ACTN1* | 4.13 | 2.07 | 0.015 |
|  | Cellular Component | GO:0005856~cytoskeleton | *MYH7, ELMO1, PBXIP1, CENPF, CTNNA1,* Uncharacterized protein (ENSBTAG00000009703), *CDC42SE1, TUBA1B, PFN1, MAPT , ARPC4, SEPT11, PDLIM7, ACTB, ACTR2, VCL, ACTN1, S100A8* | 1.67 | 4.11 | 0.042 |
|  | Molecular function | GO:0008092~cytoskeletal protein binding | *PFN1, S100B, ARPC4, PLS3 , MYH7,* Uncharacterized protein (ENSBTAG00000009703), *CTNNA1, ACTR2, ACTN1* | 2.43 | 4.63 | 0.045 |
|  | Biological Process | GO:0030029~actin filament-based process | *PFN1, ARPC4, ACTB , ELMO1 , MYH7 ,* Uncharacterized protein (ENSBTAG00000009703), *ACTN1* | 4.03 | 2.25 | 0.016 |
|  | Molecular function | GO:0003779~actin binding | *PFN1, ARPC4, PLS3 , MYH7,* Uncharacterized protein (ENSBTAG00000009703), *CTNNA1, ACTR2, ACTN1* | 3.40 | 2.00 | 0.017 |
|  | Molecular function | GO:0051015~actin filament binding | *MYH7,* Uncharacterized protein (ENSBTAG00000009703), *CTNNA1, ACTN1* | 9.51 | 4.09 | 0.039 |
|  | Cellular Component | GO:0005913~cell-cell adherens junction | *GJA1, CTNNA1, VCL* | 9.44 | 3.84 | 0.039 |
|  | Biological Process | GO:0007043~cell-cell junction assembly | *CTNNA1, VCL* | 3.85 | 5.59 | 0.051 |
|  | Cellular Component | GO:0030016~myofibril | *MYH7,* Uncharacterized protein (ENSBTAG00000009703), *VCL, ACTN1, PDLIM5* | 4.82 | 4.57 | 0.049 |
|  | Cellular Component | GO:0043292~contractile fiber | *MYH7,* Uncharacterized protein (ENSBTAG00000009703), *VCL, ACTN1, PDLIM5* | 4.44 | 5.28 | 0.059 |
|  | Biological Process | GO:0032989~cellular component morphogenesis | *GJA1, S100B, ACTB , MYH7 ,* Uncharacterized protein (ENSBTAG00000009703), *CTNNA1, CXCR4, NTNG2* | 3.21 | 2.87 | 0.021 |
|  | Biological Process | GO:0030030~cell projection organization | *GJA1, S100B, CXCR4, VCL, NTNG2 , PAK1* | 3.04 | 5.26 | 0.046 |
|  | Biological Process | GO:0044057~regulation of system process | *GJA1, S100B, CAMK2A, MYH7 ,* Uncharacterized protein (ENSBTAG00000009703), *CAV1, ANXA6, ACTN1, PLN* | 5.20 | 1.28 | 0.000 |
| Plasma lipoprotein | Cellular Component | GO:0034364~high-density lipoprotein particle | *LCAT , SAA3, SAA1* | 1.06 | 3.21 | 0.031 |
|  | Cellular Component | GO:0032994~protein-lipid complex | *LCAT , SAA3, SAA1* | 8.50 | 4.46 | 0.047 |
|  | Cellular Component | GO:0034358~plasma lipoprotein particle | *LCAT , SAA3, SAA1* | 8.50 | 4.46 | 0.047 |
|  | Cellular Component | GO:0044421~extracellular region part | *FGA, TWSG1,LOXL1, NTNG2, LAMA4 , OLFML2B, LCAT , MMP23B,LGALS1, CXCL10, SAA3, SPOCK1, SAA1, ANGPTL1, FIGF, LGALS3* | 2.12 | 8.65 | 0.007 |
|  | Cellular Component | GO:0005615~extracellular space | *LCAT ,LGALS1, SAA3, CXCL10, FGA, TWSG1, SAA1,LOXL1, ANGPTL1 , FIGF* | 2.12 | 4.24 | 0.044 |
| GTP binding | Molecular function | GO:0005525~GTP binding | *GNAI2, RIT1, SEPT11, GEM , MRAS,RASEF,* Uncharacterized protein (ENSBTAG00000037510), *GIMAP7, GBP4, TUBA1B, RASL11B* | 2.56 | 1.31 | 0.010 |
|  | Molecular function | GO:0032561~guanyl ribonucleotide binding | *GNAI2, RIT1, SEPT11, GEM , MRAS,RASEF,* Uncharacterized protein (ENSBTAG00000037510), *GIMAP7, GBP4, TUBA1B, RASL11B* | 2.50 | 1.51 | 0.012 |
|  | Molecular function | GO:0019001~guanyl nucleotide binding | *GNAI2, RIT1, SEPT11, GEM , MRAS,RASEF,* Uncharacterized protein (ENSBTAG00000037510), *GIMAP7, GBP4, TUBA1B, RASL11B* | 2.47 | 1.61 | 0.013 |
