# Supplementary material for: Size of the Ovulatory Follicle Dictates Spatial Differences in the Oviductal Transcriptome in Cattle
Source: PLoS One. 2015 Dec 23;10(12):e0145321. doi: 10.1371/journal.pone.0145321 (PMC4689418; doi:10.1371/journal.pone.0145321)
Supplement: S8 Table — Gene ontology analysis is performed with DAVID tools (http://david.abcc.ncifcrf.gov/tools.jsp). The enrichment p-values are corrected by Benjamini's methods. GO categories are presented according to their biological process, cellular component and molecular function. (DOCX) [file pone.0145321.s010.docx]

**S8 Table. Gene ontologies (GO category) of mRNA transcripts differentially expressed in day 4 Ampulla samples of the SF/SCL group.** Gene ontology analysis is performed with DAVID tools (http://david.abcc.ncifcrf.gov/tools.jsp). The enrichment p-values are corrected by Benjamini's methods. GO categories are presented according to their biological process, cellular component and molecular function.

| **Enriched process** | **Category** | **Term** | **Genes** | **Fold Enrichment** | **FDR** | ***P* Value** |
| --- | --- | --- | --- | --- | --- | --- |
| Cell development, doferenciatoin and motility | Biological Process | GO:0006928~cell motion | *PAX6 , ABI2, EDNRB, IL16, EFNA5, KDR, TGFB2, EFNB1* | 3.86 | 6.73 | 0.004 |
|  | Biological Process | GO:0007411~axon guidance | *PAX6 , EFNA5, TGFB2, EFNB1* | 9.52 | 11.86 | 0.008 |
|  | Biological Process | GO:0007409~axonogenesis | *PAX6 , EFNA5, TGFB2, EFNB1* | 5.10 | 4.92 | 0.043 |
|  | Cellular Component | GO:0043005~neuron projection | *ABI2, SNAP25, TGFB2, SYT5, GRM3 , FBXO2* | 5.11 | 6.97 | 0.006 |
|  | Biological Process | GO:0031175~neuron projection development | *PAX6 , ABI2, EFNA5, TGFB2, EFNB1* | 4.89 | 2.52 | 0.018 |
|  | Biological Process | GO:0048812~neuron projection morphogenesis | *PAX6 , EFNA5, TGFB2, EFNB1* | 4.84 | 5.39 | 0.048 |
|  | Biological Process | GO:0016477~cell migration | *PAX6 , ABI2, EDNRB, IL16, KDR, EFNB1* | 3.76 | 2.82 | 0.021 |
|  | Biological Process | GO:0051674~localization of cell | *PAX6 , ABI2, EDNRB, IL16, KDR, EFNB1* | 3.46 | 3.67 | 0.029 |
|  | Biological Process | GO:0048870~cell motility | *PAX6 , ABI2, EDNRB, IL16, KDR, EFNB1* | 3.46 | 3.67 | 0.029 |
|  | Biological Process | GO:0030855~epithelial cell differentiation | *PAX6 , GATA6, KDR, VEZF1* | 6.49 | 3.03 | 0.023 |
|  | Cellular Component | GO:0042995~cell projection | *CTNNA2, ABI2, SNAP25, SPRY4, SNTN, TGFB2, SYT5, TTLL9 , GRM3 , FBXO2* | 3.09 | 5.37 | 0.005 |
|  | Biological Process | GO:0032101~regulation of response to external stimulus | *IL16, KDR, Thrombin-F2, 5'-nucleotidase, CD276* | 5.25 | 2.04 | 0.015 |
| Voltage-gated channel activity | Molecular function | GO:0022843~voltage-gated cation channel activity | *KCNRG, KCTD1,KCNJ11 , CACNB2, KCNQ4, KCNA5, KCNAB1* | 5.32 | 2.45 | 0.002 |
|  | Molecular function | GO:0005249~voltage-gated potassium channel activity | *KCNRG, KCTD1,KCNJ11 , KCNQ4, KCNA5, KCNAB1* | 6.02 | 3.86 | 0.003 |
|  | Molecular function | GO:0022843~voltage-gated cation channel activity | *KCNRG, KCTD1,KCNJ11 , CACNB2, KCNQ4, KCNA5, KCNAB1* | 4.25 | 7.32 | 0.006 |
|  | Molecular function | GO:0005249~voltage-gated potassium channel activity | *KCNRG, KCTD1,KCNJ11 , CACNB2, KCNQ4, KCNA5, KCNAB1* | 4.25 | 7.32 | 0.006 |
|  | Molecular function | GO:0005267~potassium channel activity | *KCNRG, KCTD1,KCNJ11 , KCNQ4, KCNA5, KCNAB1* | 4.71 | 1.06 | 0.009 |
|  | Biological Process | GO:0006813~potassium ion transport | *KCNRG, KCTD1, KCNJ11, KCNQ4, KCNA5, KCNAB1* | 4.20 | 1.92 | 0.014 |
|  | Cellular Component | GO:0034703~cation channel complex | *KCNRG, KCTD1, CACNB2, KCNQ4, KCNA5* | 5.00 | 1.86 | 0.017 |
|  | Biological Process | GO:0030001~metal ion transport | *KCNRG, SLC4A4, KCTD1, KCNJ11, SLC38A11, CACNB2, SCO2, KCNQ4, KCNA5, KCNAB1* | 2.51 | 2.41 | 0.018 |
|  | Biological Process | GO:0006811~ion transport | *KCNRG, SLC4A9, KCTD1,* Gene ID ENSBTAG00000021287, *SCO2, KCNA5, KCNQ4, SLC4A4, FXYD6, KCNJ11, LASP1, SLC38A11, CACNB2, KCNAB1* | 1.96 | 3.23 | 0.025 |
|  | Biological Process | GO:0015672~monovalent inorganic cation transport | *KCNRG, SLC4A4, KCTD1, KCNJ11, SLC38A11, KCNQ4, KCNA5, KCNAB1* | 2.75 | 3.32 | 0.026 |
|  | Molecular function | GO:0005261~cation channel activity | *KCNRG, KCTD1,KCNJ11 , CACNB2, KCNQ4, KCNA5, KCNAB1* | 3.07 | 2.89 | 0.026 |
|  | Cellular Component | GO:0008076~voltage-gated potassium channel complex | *KCNRG, KCTD1, KCNQ4, KCNA5* | 5.52 | 3.46 | 0.035 |
|  | Cellular Component | GO:0034705~potassium channel complex | *KCNRG, KCTD1, KCNQ4, KCNA5* | 5.52 | 3.46 | 0.035 |
|  | Cellular Component | GO:0005886~plasma membrane | *KCNRG, CDH23, EDNRB ,USP2 , KCTD1, SPRY4,* 5'-nucleotidase (ENSBTAG00000011330), *CD276, CTNNA2, SLC4A4, HPN, SNAP25, KCNJ11 , LASP1, CDC42EP1, DGKH, ABI2, CXADR, KCNQ4, KCNA5, TIAM1, KRT19, KDR , CACNB2, GRM3* | 1.45 | 4.31 | 0.046 |
|  | Molecular function | GO:0031420~alkali metal ion binding | *SLC4A4,KCNJ11 , SLC38A11 , KCNA5, KCNAB1* | 4.82 | 2.25 | 0.019 |
| Ephrin receptor binding | Molecular function | GO:0046875~ephrin receptor binding | *EFNB1, TIAM1* | 7.23 | 3.01 | 0.027 |
| Adherens junction | Cellular Component | GO:0005912~adherens junction | *CTNNA2, ABI2, CXADR, LASP1* | 5.02 | 4.19 | 0.044 |
