# Supplementary material for: Size of the Ovulatory Follicle Dictates Spatial Differences in the Oviductal Transcriptome in Cattle
Source: PLoS One. 2015 Dec 23;10(12):e0145321. doi: 10.1371/journal.pone.0145321 (PMC4689418; doi:10.1371/journal.pone.0145321)
Supplement: S9 Table — Gene ontology analysis is performed with DAVID tools (http://david.abcc.ncifcrf.gov/tools.jsp). The enrichment p-values are corrected by Benjamini's methods. GO categories are presented according to their biological process, cellular component and molecular function. (DOCX) [file pone.0145321.s011.docx]

**S9 Table. Gene ontologies (GO category) of mRNA transcripts differentially expressed in day 4 Isthmus samples of the LF/LCL group.** Gene ontology analysis is performed with DAVID tools (http://david.abcc.ncifcrf.gov/tools.jsp). The enrichment p-values are corrected by Benjamini's methods. GO categories are presented according to their biological process, cellular component and molecular function.

| **Enriched process** | **Category** | **Term** | **Genes** | **Fold Enrichment** | **FDR** | ***P* Value** |
| --- | --- | --- | --- | --- | --- | --- |
| Extracellular matrix | Biological Process | GO:0007155~cell adhesion | *E-CADHERIN, MGC142792, VCAN, AGT, COL3A1, LAMC1 , EPDR1 , PCDH18, COL18A1, DSG3, CDH19, FN1, ITGA11,* | 3.46 | 0.52 | 0.000 |
|  | Biological Process | GO:0022610~biological adhesion | *E-CADHERIN , MGC142792, VCAN, AGT , COL3A1, LAMC1, EPDR1, PCDH18, COL18A1, DSG3, CDH19, FN1, ITGA11* | 3.46 | 0.52 | 0.000 |
|  | Biological Process | GO:0007160~cell-matrix adhesion | *EPDR1, MGC142792, FN1, AGT, COL3A1* | 11.30 | 1.44 | 0.001 |
|  | Biological Process | GO:0031589~cell-substrate adhesion | *EPDR1, MGC142792, FN1, AGT, COL3A1* | 10.17 | 2.14 | 0.001 |
|  | Biological Process | GO:0043588~skin development | *COL1A1, NGFR, ADAMTS2, COL3A1* | 23.24 | 0.92 | 0.001 |
|  | Biological Process | GO:0048146~positive regulation of fibroblast proliferation | *BMI1, NGFR, IGF-I* | 22.18 | 11.34 | 0.008 |
|  | Molecular function | GO:0030247~polysaccharide binding | *VCAN, FN1, CCDC80, ENPP1, BMP4* | 5.86 | 12.46 | 0.010 |
|  | Molecular function | GO:0001871~pattern binding | *VCAN, FN1, CCDC80, ENPP1, BMP4* | 5.86 | 12.46 | 0.010 |
|  | Cellular Component | GO:0005581~collagen | *COL4A2, COL1A1, LOX, COL3A1, COL4A1, COL4A6, LUM* | 30.10 | 0.00 | 0.000 |
|  | Biological Process | GO:0030199~collagen fibril organization | *COL1A1, ADAMTS2, LOX, COL3A1* | 23.24 | 0.92 | 0.001 |
|  | Cellular Component | GO:0005583~fibrillar collagen | *COL1A1, COL3A1, LUM* | 38.70 | 2.82 | 0.002 |
|  | Molecular function | GO:0019838~growth factor binding | *COL1A1, NGFR, WISP1, LTBP2, COL3A1* | 7.82 | 4.67 | 0.004 |
|  | Molecular function | GO:0005539~glycosaminoglycan binding | *VCAN, FN1, CCDC80, BMP4* | 5.70 | 35.11 | 0.032 |
|  | Molecular function | GO:0004222~metalloendopeptidase activity | *ADAM23, ADAMTS2, MMP14, ADAMTS4, MMP24* | 4.75 | 23.86 | 0.020 |
|  | Biological Process | GO:0010810~regulation of cell-substrate adhesion | *COL1A1, COL8A1, CCDC80* | 8.72 | 52.19 | 0.045 |
|  | Cellular Component | GO:0031012~extracellular matrix | *COL4A2, VCAN, ADAMTS4, ASPN, CCDC80, LAMC1 , COL3A1, COL4A6, FBLN1, COL6A2, LTBP1, COL18A1 , COL1A1, SMOC2, MMP14, ADAMTS2, MMP24, FN1, LOX, COL4A1, LUM, BMP4* | 9.57 | 0.00 | 0.000 |
|  | Cellular Component | GO:0005578~proteinaceous extracellular matrix | *COL4A2, VCAN, ADAMTS4, ASPN, CCDC80, COL3A1, COL4A6, FBLN1, LTBP1, COL1A1, SMOC2, ADAMTS2, MMP14, MMP24, FN1, LOX, COL4A1, LUM, BMP4* | 9.02 | 0.00 | 0.000 |
|  | Cellular Component | GO:0044420~extracellular matrix part | *COL4A2, COL1A1, FN1, LOX, CCDC80, COL3A1, COL4A1, FBLN1, COL4A6, LUM* | 14.07 | 0.00 | 0.000 |
|  | Molecular function | GO:0005201~extracellular matrix structural constituent | *COL4A2, COL18A1 , COL1A1, COL3A1, COL4A1, COL4A6* | 22.78 | 0.01 | 0.000 |
|  | Biological Process | GO:0043062~extracellular structure organization | *COL1A1, CHRNA1, ADAMTS2, AGT , LOX, CCDC80, COL3A1, FBLN1* | 8.91 | 0.04 | 0.000 |
|  | Biological Process | GO:0030198~extracellular matrix organization | *COL1A1, ADAMTS2, AGT , LOX, CCDC80, COL3A1, FBLN1* | 9.99 | 0.10 | 0.000 |
|  | Cellular Component | GO:0044421~extracellular region part | *MYOC, ADAMTS4, AGT , CCDC80, COL4A6, LTBP1, COL18A1 , IL1B , CCL21 , MMP14, MMP24, FN1, COL4A2, VCAN, ASPN, IGF-I, COL3A1, LAMC1 , FBLN1, COL6A2, COL1A1, SMOC2, ADAMTS2, LOX, ANGPTL1 , COL4A1, LUM, BMP4* | 5.08 | 0.00 | 0.000 |
|  | Cellular Component | GO:0005576~extracellular region | *MYOC, TFPI2 , TF, ADAMTS4, AGT , CCDC80, COL4A6, LTBP1, EPDR1 , COL18A1 , PI16, IL1B, CCL21 , MMP14, CST9L, C7, MMP24, WISP1, FN1, HYAL1 , SRPX2 , COL4A2, VCAN, ASPN, IGF-I, LAMC1 , COL3A1, FBLN1, COL6A2, COL1A1, ACPP , ADAMTS2, SMOC2, LOX, ANGPTL1 , COL4A1, LUM, BMP4* | 3.30 | 0.00 | 0.000 |
|  | Cellular Component | GO:0005604~basement membrane | *COL4A2, FN1, CCDC80, COL4A1, FBLN1* | 10.75 | 1.30 | 0.001 |
|  | Cellular Component | GO:0044459~plasma membrane part | *TF, E-CADHERIN , MGC142792, SLC12A2, KCNA4, CACNA1G, ROBO2, F2R, CHRNA3, GABRA1 , CHRNA1, KCNMA1, C7, SV2A, FN1, ITGA11* | 1.73 | 36.23 | 0.036 |
|  | Biological Process | GO:0003006~reproductive developmental process | *HNF1A, PTGDR, FOXL2, AGT , BMP4* | 4.24 | 37.87 | 0.029 |
|  | Biological Process | GO:0030324~lung development | *ADAMTS2, MMP14, LOX, BMP4* | 6.78 | 28.05 | 0.020 |
|  | Biological Process | GO:0060541~respiratory system development | *ADAMTS2, MMP14, LOX, BMP4* | 6.64 | 29.38 | 0.022 |
|  | Biological Process | GO:0008544~epidermis development | *COL1A1, NGFR, ADAMTS2, COL3A1* | 6.64 | 29.38 | 0.022 |
|  | Cellular Component | GO:0005886~plasma membrane | *TF, E-CADHERIN , MGC142792, SLC12A2, NGFR, EDNRA , KCNA4, CACNA1G, ROBO2, COL6A2, CALD1, F2R, CAPN1, PCDH18, CHRNA3, GABRA1 , CHRNA1, KCNMA1, DSG3, PTGDR, SV2A, C7 , FN1, ITGA11* | 1.57 | 24.30 | 0.023 |
|  | Biological Process | GO:0007398~ectoderm development | *COL1A1, NGFR, ADAMTS2, COL3A1* | 6.26 | 33.45 | 0.025 |
|  | Cellular Component | GO:0045177~apical part of cell | *KCNMA1, E-CADHERIN , SLC12A2, FN1* | 5.16 | 39.97 | 0.041 |
|  | Molecular function | GO:0016504~peptidase activator activity | *FN1, FBLN1* | 39.87 | 48.35 | 0.049 |
|  | Molecular function | GO:0004175~endopeptidase activity | *CAPN11, CAPN1, ADAM23, TMPRSS6, ADAMTS2, MMP14, C1S, ADAMTS4, MMP24, NRIP3* | 2.56 | 18.68 | 0.016 |
|  | Biological Process | GO:0007167~enzyme linked receptor protein signaling pathway | *PPM1L, GRB10, ANGPTL1 , COL3A1, C-MET, BMP4* | 3.54 | 34.74 | 0.026 |
|  | Molecular function | GO:0030246~carbohydrate binding | *VCAN, FN1, COLEC12, CCDC80, ENPP1, BMP4* | 3.23 | 39.25 | 0.037 |
|  | Biological Process | GO:0031328~positive regulation of cellular biosynthetic process | *F2R, HNF1A, IL1B , FOXL2, FAM129A, AGT , IGF-I, BMP4* | 2.53 | 45.28 | 0.037 |
|  | Biological Process | GO:0051604~protein maturation | *ADAMTS2, MMP14, C1S, C7* | 5.92 | 37.62 | 0.029 |
|  | Biological Process | GO:0016485~protein processing | *ADAMTS2, MMP14, C1S, C7* | 6.26 | 33.45 | 0.025 |
|  | Biological Process | GO:0051605~protein maturation by peptide bond cleavage | *ADAMTS2, MMP14, C1S, C7* | 8.56 | 16.00 | 0.011 |
|  | Biological Process | GO:0009891~positive regulation of biosynthetic process | *F2R, HNF1A, IL1B , FOXL2, FAM129A, AGT , IGF-I, BMP4* | 2.50 | 47.08 | 0.039 |
|  | Biological Process | GO:0001568~blood vessel development | *ELK3, COL1A1, AGT , LOX, COL3A1, BMP4* | 4.14 | 20.66 | 0.014 |
|  | Biological Process | GO:0001944~vasculature development | *ELK3, COL1A1, AGT , LOX, COL3A1, BMP4* | 4.03 | 22.56 | 0.016 |
| Morphogenesis and cell proliferation | Biological Process | GO:0048754~branching morphogenesis of a tube | *MMP14, AGT , IGF-I, BMP4* | 10.85 | 8.59 | 0.006 |
|  | Biological Process | GO:0035295~tube development | *ADAMTS2, MMP14, AGT , LOX, IGF-I, BMP4* | 4.98 | 10.26 | 0.007 |
|  | Biological Process | GO:0030323~respiratory tube development | *ADAMTS2, MMP14, LOX, BMP4* | 6.78 | 28.05 | 0.020 |
|  | Biological Process | GO:0035239~tube morphogenesis | *MMP14, AGT , IGF-I, BMP4* | 6.03 | 36.22 | 0.028 |
|  | Biological Process | GO:0001763~morphogenesis of a branching structure | *MMP14, AGT , IGF-I, BMP4* | 9.30 | 12.95 | 0.009 |
|  | Biological Process | GO:0048729~tissue morphogenesis | *HNF1A, COL1A1, NGFR, AGT , BMP4* | 5.08 | 22.94 | 0.016 |
|  | Biological Process | GO:0008283~cell proliferation | *COL8A1, AGT , IGF-I, PDGFD, C-MET, BMP4* | 4.40 | 16.54 | 0.011 |
|  | Biological Process | GO:0051450~myoblast proliferation | *IGF-I, C-MET* | 40.67 | 54.23 | 0.048 |
|  | Biological Process | GO:0048145~regulation of fibroblast proliferation | *BMI1, NGFR, IGF-I* | 15.25 | 22.40 | 0.016 |
|  | Biological Process | GO:0010941~regulation of cell death | *F2R, IL1B , KCNMA1, E-CADHERIN , NGFR, FOXL2, AGT , IGF-I, BMP4* | 2.45 | 37.65 | 0.029 |
|  | Biological Process | GO:0001649~osteoblast differentiation | *COL1A1, IGF-I, BMP4* | 12.20 | 32.34 | 0.024 |
|  | Biological Process | GO:0042692~muscle cell differentiation | *CHRNA1, AGT , IGF-I, BMP4* | 5.33 | 46.07 | 0.038 |
|  | Biological Process | GO:0051145~smooth muscle cell differentiation | *AGT, BMP4* | 40.67 | 54.23 | 0.048 |
| Homeostasis | Biological Process | GO:0042592~homeostatic process | *F2R, SELV, HNF1A, CHRNA1, IL1B , KCNMA1, TF, SV2A, IGF-I, CACNA1G, ITPR1, BMP4* | 2.77 | 5.73 | 0.004 |
|  | Biological Process | GO:0048878~chemical homeostasis | *F2R, HNF1A, CHRNA1, KCNMA1, TF, SV2A, IGF-I, CACNA1G, ITPR1* | 3.36 | 7.72 | 0.005 |
|  | Biological Process | GO:0006873~cellular ion homeostasis | *F2R, CHRNA1, KCNMA1, TF, SV2A, CACNA1G, ITPR1* | 3.77 | 15.04 | 0.010 |
|  | Biological Process | GO:0055082~cellular chemical homeostasis | *F2R, CHRNA1, KCNMA1, TF, SV2A, CACNA1G, ITPR1* | 3.72 | 15.91 | 0.011 |
|  | Biological Process | GO:0019725~cellular homeostasis | *F2R, SELV, CHRNA1, KCNMA1, TF, SV2A, CACNA1G, ITPR1* | 3.03 | 22.41 | 0.016 |
|  | Biological Process | GO:0050801~ion homeostasis | *F2R, CHRNA1, KCNMA1, TF, SV2A, CACNA1G, ITPR1* | 3.39 | 23.34 | 0.017 |
|  | Biological Process | GO:0030003~cellular cation homeostasis | *F2R, KCNMA1, TF, SV2A, ITPR1* | 4.11 | 40.88 | 0.032 |
|  | Biological Process | GO:0006875~cellular metal ion homeostasis | *F2R, KCNMA1, SV2A, ITPR1* | 5.01 | 51.64 | 0.045 |
|  | Biological Process | GO:0055065~metal ion homeostasis | *F2R, KCNMA1, SV2A, ITPR1* | 4.78 | 55.71 | 0.050 |
| Ion transport | Molecular function | GO:0005509~calcium ion binding | *CAPN11, LTBP2, EPDR1 , LTBP1, NPNT, DSG3, MMP14, CDH19, MMP24, FKBP9 , FKBP10, FBLN7, MYLK, E-CADHERIN , VCAN, ITPR1, FBLN1, CAPN1, PCDH18, KCNMA1, ACTN3, PLCB1, SMOC2, C1S, PLCL1, COLEC12* | 3.93 | 0.00 | 0.000 |
|  | Molecular function | GO:0005216~ion channel activity | *CHRNA3, GABRA1 , CHRNA1, CLCN4, KCNMA1, GRIN2D, CLIC4, P2RX3, GRIA3, KCNA4, CACNA1G, ITPR1* | 3.57 | 0.64 | 0.000 |
|  | Molecular function | GO:0043167~ion binding | *CAPN11, MOCOS, TF, PLOD1, MGC142792, RGNEF, ADAMTS4, BNC1, KCNA4, LTBP2, ENPP1, LTBP1, BMI1, EPDR1 , GABRA1 , NPNT, CLIC4, DSG3, MMP14, CDH19, NMNAT1, MMP24, TRIM9, FKBP9 , JAZF1, FKBP10, PKLR, PPM1L, FBLN7, ADAM23, MYLK, E-CADHERIN , VCAN, ITPR1, FBLN1, PCDH18, CAPN1, KCNMA1, PLCB1, ACTN3, ADAMTS2, SMOC2, C1S, PLCL1, LOX, COLEC12, RORB* | 1.54 | 1.22 | 0.001 |
|  | Molecular function | GO:0022836~gated channel activity | *CHRNA3, GABRA1 , CHRNA1, CLCN4, KCNMA1, GRIN2D, CLIC4, GRIA3, KCNA4, CACNA1G, ITPR1* | 4.30 | 0.29 | 0.000 |
|  | Molecular function | GO:0022838~substrate specific channel activity | *CHRNA3, GABRA1 , CHRNA1, CLCN4, KCNMA1, GRIN2D, CLIC4, P2RX3, GRIA3, KCNA4, CACNA1G, ITPR1* | 3.53 | 0.70 | 0.001 |
|  | Cellular Component | GO:0034702~ion channel complex | *CHRNA3, GABRA1 , CHRNA1, KCNMA1, CLIC4, KCNA4, CACNA1G* | 5.59 | 1.73 | 0.001 |
|  | Molecular function | GO:0022803~passive transmembrane transporter activity | *CHRNA3, GABRA1 , CHRNA1, CLCN4, KCNMA1, GRIN2D, CLIC4, P2RX3, GRIA3, KCNA4, CACNA1G, ITPR1* | 3.48 | 0.79 | 0.001 |
|  | Molecular function | GO:0015267~channel activity | *CHRNA3, GABRA1 , CHRNA1, CLCN4, KCNMA1, GRIN2D, CLIC4, P2RX3, GRIA3, KCNA4, CACNA1G, ITPR1* | 3.48 | 0.79 | 0.001 |
|  | Biological Process | GO:0006811~ion transport | *CLCN4, TF, SLC12A2, GRIA3, KCNA4, CACNA1G, ITPR1, F2R, CHRNA3, GABRA1 , CHRNA1, GRIN2D, CLIC4, KCNMA1, P2RX3, ATP2B3* | 2.55 | 2.00 | 0.001 |
|  | Molecular function | GO:0046872~metal ion binding | *CAPN11, MOCOS, TF, PLOD1, MGC142792, RGNEF, ADAMTS4, BNC1, KCNA4, LTBP2, ENPP1, LTBP1, BMI1, EPDR1 , NPNT, DSG3, MMP14, CDH19, NMNAT1, MMP24, TRIM9, FKBP9 , JAZF1, FKBP10, PKLR, PPM1L, FBLN7, ADAM23, MYLK, E-CADHERIN , VCAN, ITPR1, FBLN1, PCDH18, CAPN1, KCNMA1, PLCB1, ACTN3, ADAMTS2, SMOC2, C1S, PLCL1, LOX, COLEC12, RORB* | 1.50 | 2.76 | 0.002 |
|  | Molecular function | GO:0043169~cation binding | *CAPN11, MOCOS, TF, PLOD1, MGC142792, RGNEF, ADAMTS4, BNC1, KCNA4, LTBP2, ENPP1, LTBP1, BMI1, EPDR1 , NPNT, DSG3, MMP14, CDH19, NMNAT1, MMP24, TRIM9, FKBP9 , JAZF1, FKBP10, PKLR, PPM1L, FBLN7, ADAM23, MYLK, E-CADHERIN , VCAN, ITPR1, FBLN1, PCDH18, CAPN1, KCNMA1, PLCB1, ACTN3, ADAMTS2, SMOC2, C1S, PLCL1, LOX, COLEC12, RORB* | 1.49 | 3.50 | 0.003 |
|  | Molecular function | GO:0022834~ligand-gated channel activity | *CHRNA3, GABRA1 , CHRNA1, GRIN2D, GRIA3, ITPR1* | 5.44 | 6.03 | 0.005 |
|  | Molecular function | GO:0015276~ligand-gated ion channel activity | *CHRNA3, GABRA1 , CHRNA1, GRIN2D, GRIA3, ITPR1* | 5.44 | 6.03 | 0.005 |
|  | Biological Process | GO:0006821~chloride transport | *GABRA1 , CLCN4, CLIC4, SLC12A2* | 10.17 | 10.22 | 0.007 |
|  | Molecular function | GO:0005230~extracellular ligand-gated ion channel activity | *CHRNA3, GABRA1 , CHRNA1, GRIN2D, GRIA3* | 6.54 | 8.69 | 0.007 |
|  | Biological Process | GO:0015698~inorganic anion transport | *GABRA1 , CLCN4, CLIC4, SLC12A2* | 6.78 | 28.05 | 0.020 |
|  | Molecular function | GO:0046873~metal ion transmembrane transporter activity | *CHRNA3, CHRNA1, KCNMA1, KCNA4, ATP2B3, CACNA1G, ITPR1* | 2.88 | 36.47 | 0.034 |
|  | Biological Process | GO:0015674~di-, tri-valent inorganic cation transport | *F2R, TF, ATP2B3, CACNA1G, ITPR1* | 3.91 | 45.95 | 0.038 |
| Muscle contraction | Biological Process | GO:0006936~muscle contraction | *CHRNA1, KCNMA1, ACTN3, AGT , CALD1* | 9.92 | 2.35 | 0.001 |
|  | Biological Process | GO:0003012~muscle system process | *CHRNA1, KCNMA1, ACTN3, AGT , IGF-I, CALD1* | 10.17 | 0.44 | 0.000 |
