# Supplementary material for: Size of the Ovulatory Follicle Dictates Spatial Differences in the Oviductal Transcriptome in Cattle
Source: PLoS One. 2015 Dec 23;10(12):e0145321. doi: 10.1371/journal.pone.0145321 (PMC4689418; doi:10.1371/journal.pone.0145321)
Supplement: S10 Table — Gene ontology analysis is performed with DAVID tools (http://david.abcc.ncifcrf.gov/tools.jsp). The enrichment p-values are corrected by Benjamini's methods. GO categories are presented according to their biological process, cellular component and molecular function. (DOCX) [file pone.0145321.s012.docx]

**S10 Table. Gene ontologies (GO category) of mRNA transcripts differentially expressed in day 4 Isthmus samples of the SF/SCL group.** Gene ontology analysis is performed with DAVID tools (http://david.abcc.ncifcrf.gov/tools.jsp). The enrichment p-values are corrected by Benjamini's methods. GO categories are presented according to their biological process, cellular component and molecular function.

| **Enriched process** | **Category** | **Term** | **Genes** | **Fold Enrichment** | **FDR** | ***P* Value** |
| --- | --- | --- | --- | --- | --- | --- |
| Protein kinase regulator activity | Molecular function | GO:0008603~cAMP-dependent protein kinase regulator activity | *ROPN1, SPA17, ROPN1L, CABYR* | 25.69 | 0.53 | 0.000 |
|  | Molecular function | GO:0019207~kinase regulator activity | *ROPN1, S100B, SPA17, ROPN1L, CABYR* | 6.30 | 9.83 | 0.008 |
|  | Molecular function | GO:0019887~protein kinase regulator activity | *ROPN1, SPA17, ROPN1L, CABYR* | 5.97 | 31.72 | 0.028 |
| Apoptosis | Biological Process | GO:0008219~cell death | *TMBIM6, BNIPL, WDR92, CLU, TNS4, ITM2B, CASP8, CASP13, IFI6* | 2.68 | 25.38 | 0.018 |
|  | Biological Process | GO:0016265~death | *TMBIM6, BNIPL, WDR92, CLU, TNS4, ITM2B, CASP8, CASP13, IFI6* | 2.62 | 28.26 | 0.021 |
|  | Biological Process | GO:0012501~programmed cell death | *TMBIM6, BNIPL, WDR92, TNS4, ITM2B, CASP8, CASP13, IFI6* | 2.63 | 39.56 | 0.031 |
|  | Biological Process | GO:0006915~apoptosis | *TMBIM6, BNIPL, WDR92, TNS4, ITM2B, CASP8, CASP13, IFI6* | 2.69 | 36.58 | 0.029 |
| Protein folding | Biological Process | GO:0051085~chaperone mediated protein folding requiring cofactor | *HSPA8, DNAJB1, HSPH1* | 17.24 | 17.68 | 0.012 |
|  | Biological Process | GO:0051084~'de novo' posttranslational protein folding | *HSPA8, DNAJB1, HSPH1* | 15.80 | 20.65 | 0.015 |
|  | Biological Process | GO:0006458~'de novo' protein folding | *HSPA8, DNAJB1, HSPH1* | 15.80 | 20.65 | 0.015 |
| Cellular metabolism | Biological Process | GO:0006575~cellular amino acid derivative metabolic process | *LY6E, GGT6, GSTK1, PCYOX1L, VNN1, SLC1A3, MGST1* | 5.27 | 3.11 | 0.002 |
|  | Biological Process | GO:0006749~glutathione metabolic process | *GGT6, GSTK1, MGST1* | 15.80 | 20.65 | 0.015 |
|  | Biological Process | GO:0051186~cofactor metabolic process | *GGT6, GSTK1, VNN1, MGST1, BLVRA, NADSYN1* | 3.16 | 47.75 | 0.040 |
|  | Biological Process | GO:0006790~sulfur metabolic process | *GGT6, GSTK1, MGST1, HS3ST5* | 4.86 | 53.88 | 0.048 |
|  | Molecular function | GO:0004364~glutathione transferase activity | *GSTK1, MGST1, MGST2* | 9.63 | 39.72 | 0.038 |
|  | Molecular function | GO:0016670~oxidoreductase activity, acting on sulfur group of donors, oxygen as acceptor | *PCYOX1L, QSOX1* | 42.82 | 46.07 | 0.046 |
| Antigen processing and presentation | Biological Process | GO:0019882~antigen processing and presentation | *BOLA, FCGRT, IFI30, HLA-DMB, AZGP1* | 3.85 | 47.25 | 0.040 |
| Nucleotide biosynthetic process | Biological Process | GO:0006164~purine nucleotide biosynthetic process | *GUCY1B1 , HPRT1, ATP5F1, ATP5S, ADSS,* Uncharacterized protein (ENSBTAG00000005217), *GUCY2C* | 3.24 | 44.62 | 0.037 |
|  | Biological Process | GO:0034404~nucleobase, nucleoside and nucleotide biosynthetic process | *GUCY1B1 , HPRT1, ATP5F1, ATP5S, ADSS,* Uncharacterized protein (ENSBTAG00000005217), *NADSYN1, GUCY2C* | 2.82 | 44.83 | 0.037 |
|  | Biological Process | GO:0034654~nucleobase, nucleoside, nucleotide and nucleic acid biosynthetic process | *GUCY1B1 , HPRT1, ATP5F1, ATP5S, ADSS,* Uncharacterized protein (ENSBTAG00000005217), *NADSYN1, GUCY2C* | 2.82 | 44.83 | 0.037 |
|  | Biological Process | GO:0009165~nucleotide biosynthetic process | *GUCY1B1 , HPRT1, ATP5F1, ATP5S, ADSS,* Uncharacterized protein (ENSBTAG00000005217), *NADSYN1, GUCY2C* | 2.95 | 38.73 | 0.031 |
| Lipid biosynthetic process | Biological Process | GO:0006631~fatty acid metabolic process | *PHYH, FADS6, SC5DL, ACAA2, RNPEP, CPT1B, MGST2* | 3.88 | 13.26 | 0.009 |
|  | Biological Process | GO:0008610~lipid biosynthetic process | *PIGW, MBOAT7, FADS6, SC5DL, CEPT1, RNPEP, CYB5R1, MGST2* | 2.99 | 23.68 | 0.017 |
